# Investigation of pathogenic germline variants in gastric cancer and development of “GasCanBase” database

**DOI:** 10.1002/cnr2.1906

**Published:** 2023-10-22

**Authors:** Mohammad Uzzal Hossain, Ishtiaque Ahammad, Md. Moniruzzaman, Mahbuba Akter Lubna, Arittra Bhattacharjee, Zeshan Mahmud Chowdhury, Istiak Ahmed, Md. Billal Hosen, Shourov Biswas, Keshob Chandra Das, Chaman Ara Keya, Md. Salimullah

**Affiliations:** ^1^ Bioinformatics Division National Institute of Biotechnology Dhaka Bangladesh; ^2^ Molecular Biotechnology Division National Institute of Biotechnology Dhaka Bangladesh; ^3^ Department of Pharmacy Noakhali Science and Technology University Noakhali Bangladesh; ^4^ Department of Clinical Oncology Bangabandhu Sheikh Mujib Medical University Dhaka Bangladesh; ^5^ Department of Biochemistry and Microbiology North South University Dhaka Bangladesh

**Keywords:** bioinformatics, database, GasCanBase, gastric cancer, polymorphism, SNP

## Abstract

**Background:**

Gastric cancer, which is also known as stomach cancer, can be influenced by both germline and somatic mutations. Non‐synonymous Single Nucleotide Polymorphisms (nsSNPs) in germline have long been reported to play a pivotal role in cancer progression.

**Aim:**

The aim of this study is to examine the nsSNP in GC‐associated genes. The study also aims to develop a database with extensive information regarding the nsSNPs in the GC‐associated genes and their impacts.

**Methods and Results:**

A total of 34,588 nsSNPs from 1,493,460 SNPs of the 40 genes were extracted from the available SNP database. Drug binding and energy minimization were examined by molecular docking and YASARA. To validate the existence of the germline CDH1 gene mutation (rs34466743) in the isolated blood DNA of gastric cancer (GC) patients, polymerase chain reaction (PCR) and DNA sequencing were performed. According to the results of the gene network analysis, 17 genes may interact with other types of cancer. A total of 11,363 nsSNPs were detected within the 40 GC genes. Among these, 474 nsSNPs were predicted to be damaging and 40 to be the most damaging. The SNPs in domain regions were thought to be strong candidates that alter protein functions. Our findings proposed that most of the selected nsSNPs were within the domains or motif regions. Free Energy Deviation calculation of protein structure pointed toward noteworthy changes in the structure of each protein that can demolish its natural function. Subsequently, drug binding confirmed the structural variation and the ineffectiveness of the drug against the mutant model in individuals with these germline variants. Furthermore, in vitro analysis of the rs34466743 germline variant from the CDH1 gene confirmed the strength and robustness of the pipeline that could expand the somatic alteration for causing cancer. In addition, a comprehensive gastric cancer polymorphism database named “GasCanBase” was developed to make data available to researchers.

**Conclusion:**

The findings of this study and the “GasCanBase” database may greatly contribute to our understanding of molecular epidemiology and the development of precise therapeutics for gastric cancer. GasCanBase is available at: https://www.gascanbase.com/.

## 1 INTRODUCTION

Identification of genetic risk factors is one of the focal areas in the study of various complex diseases.^1^, ^2^ A substantial number of recent studies identified single have nucleotide polymorphisms (SNPs) as the most abundant class of genetic variations. SNP is a change in the single base of the DNA sequence. A typical human genome possesses 4.1–5.0 million genetic variations compared to the reference human genome with >99.9% of the variations being SNPs or short indels.^3^ SNPs are found both in non‐coding and coding regions of DNA at a frequency of one in every 100–300 base pairs.^4^, ^5^, ^6^, ^7^, ^8^ Despite being more frequently occurring in the non‐coding regions, synonymous SNPs and non‐synonymous SNPs (nsSNPs) usually do not exert any significant impact on the structure and function of proteins.^9^, ^10^ However, nsSNPs in the coding regions have drawn major attention as they have the capability of altering amino acid residues.^11^ They can affect translation, post‐translational modifications, protein–protein interactions and many other biological processes.^4^, ^12^, ^13^ They can alter protein functions by inactivating active sites of enzymes, deflecting the folding patterns, changing the solubility and stability, and so on.^14^, ^15^, ^16^, ^17^ Moreover, nsSNPs have a significant impact on the functional diversity of coded proteins and are found to promote pathogenesis in various types of cancer,^18^, ^19^ sickle‐cell anemia,^20^ β‐thalassemia,^21^ cystic fibrosis,^22^ Alzheimer's disease, and so forth.^23^

Gastric cancer (GC), commonly known as stomach cancer, continues to be one of the deadliest types of cancer in the world, with the third‐highest lethality and the fourth‐highest morbidity rate.^24^, ^25^ The majority of instances of stomach cancer are centered in East Asia, and the incidence of GC is significantly higher (70%) in developing nations than in developed countries.^26^ Despite massive efforts to reduce the mortality and morbidity associated with GC, the rate of survival is still pretty low (29.6%).^27^ Therefore, the necessity to identify the genes that are involved in GC progression is indispensable. In the past few years, a lot of advancements have been made to detect the genes involved in gastric carcinogenesis. The effect of deleterious mutations on the instigation of GC has been well documented by previous studies.^28^, ^29^, ^30^, ^31^ Although several public databases, such as dbSNP, GWAS Central, SwissVar, and so forth are available for searching SNPs, it is still strenuous work to fish out the most deleterious mutation involved. Moreover, the accuracy of the predicted potentiality of the deleterious nsSNPs is also a great challenge.^32^, ^33^, ^34^ Several computational resources have been developed over the years to predict the impact of deleterious nsSNPs in candidate genes based on sequence conservation,^35^ resolved or modeled protein structures,^36^ physicochemical properties of polypeptides, and so forth.^16^, ^37^ In silico approaches were undertaken in numerous studies to identify and evaluate the impact of deleterious nsSNPs in a wide range of genes^38^, ^39^, ^40^, ^41^, ^42^, ^43^, ^44^ using computational algorithms.^45^, ^46^, ^47^, ^48^, ^49^ No functional study by computational algorithms has been conducted so far on GC genes. Therefore, the nsSNPs in GC genes are still being investigated for their detrimental influence on both germline and somatic genetics.

As a result of all of these developments, several in silico databases have been created that focus on specific cancers and cancer‐related genes, including the Human Prostate Gene DataBase (PGDB),^50^ Colon Rectal Cancer Gene Database (CoReCG),^51^ Dragon Database of genes implicated in Esophageal Cancer, (DDEC),^52^ Cervical Cancer gene Database (CCDB),^53^ Renal Cancer Gene Database (RCDB),^54^ Cancer Predisposition Gene database (dbCPG),^55^ and Human Cellular Senescence Gene Database (HCSGD).^56^ However, a database specializing in GC polymorphisms did not exist until now. Database enriched with GC genes that are also interacting with other types of cancer, PCR‐RFLP Primers, Allele‐Specific primers, Domain and Motif association, Restriction enzymes, and Impact of nsSNPs on Drug Binding might drive novel research on GC cancer.

Through the utilization of several systems biology and bioinformatics tools, the current study set out to investigate the nsSNPs in GC‐associated genes. Apart from identifying the most damaging nsSNPs, the structural and functional impact of these nsSNPs was investigated through 3D Modeling, free Energy deviation analysis, drug‐binding evaluation, and so on. DNA sequencing was used to validate and confirm a selected nsSNP to construct a methodological workflow. Finally, a database was developed that contained a plethora of information regarding nsSNPs in the GC‐associated genes and their impacts.

## 2 MATERIALS AND METHODS

### 2.1 Investigation of common cancer genes through network analysis

To extract the most common genes involved in GC, at first, a comprehensive exploration of genes associated with gastric carcinogenesis was conducted. Initially, a total of 40 genes were selected that were implicated in GC based on the highest number of literature resources in the Cancer Genetics Web database (http://www.cancer-genetics.org/). In addition, genes associated with various types of cancer such as prostate, colon, bladder, breast, and lung cancer, were obtained. The interactions between GC and other cancer‐related genes were visualized with the Cytoscape network analysis tool.^57^ Subsequently, only the genes found to be common between prostate, colon, bladder, breast, lung cancer, and GC were selected to probe their interactions with one another. Following this, the GeneMania tool was utilized to reveal the representing group and their interactions with the other genes.

### 2.2 Functional impact analysis of potentially damaging nsSNPs

All the available SNPs of 40 GC‐related genes were from the online database, Online Mendelian Inheritance in Man (OMIM).^58^ The NCBI dbSNP (Database of Single Nucleotide Polymorphism) was used to collect all SNP information (protein accession number and SNP ID) of the 40 genes.^59^ The protein sequences and structures were retrieved from the freely accessible resources Uniprot (Universal Protein Resource)^60^ and RCSB (Research Collaboratory for Structural Bioinformatics) protein databank^61^ respectively. These SNPs were then analyzed to filter out the missense nsSNPs. The rs IDs of each SNP of all the genes were submitted as a query sequence to Sorting Intolerant from Tolerant (SIFT)^62^ where a value of ≤0.05 signified the deleterious effect of nonsynonymous variants on protein function. The SIFT program presumes the functional consequence of an SNP. This web server can distinguish between polymorphisms that are functionally neutral and those that are detrimental using the SWISSPROT, nr, and TrEMBL databases.^63^

In addition, PolyPhen‐2 (Polymorphism Phenotyping v2) was employed to analyze the SNPs in the context of proteins. This bioinformatics tool predicts the possible impact of amino acid substitutions on the structure and function of human proteins using physical consideration which determines how changes in amino acid might have an effect on the local protein structure and interactions and also evolutionary comparative considerations that examine the variant site across different species to check if they are conserved over the evolutionary time.^64^

Five other tools namely, SNP&GO,^65^ PANTHER,^66^ MutPred,^67^ SNAP2,^68^ and P‐MUT^69^ were applied for the characterization and identification of the deleterious/damaged nsSNPs. The support vector machine (SVM) method was used for Polymorphism Database (SNPs) and Gene Ontology (GO) analysis tools, where the Protein Analysis Through Evolutionary Relationships (PANTHER) calculates the subPSEC (substitution position‐specific evolutionary conservation) score for the function of coding nsSNPs.^65^, ^66^ The P‐mut tool uses a variety of sequence information to label mutations and lets neural networks process the prediction in which probability scores higher than 0.5 divulges the disease‐related effect of mutation on the protein function.^69^

### 2.3 Impact of selected nsSNPs on the gene products

#### 2.3.1 Domain mapping of the nsSNPs

The consensus protein sequences were retrieved from the NCBI protein database and then were subjected to ScanProsite (http://prosite.expasy.org/scanprosite/),^70^ Pfam (https://pfam.xfam.org/)^71^ and InterPro (https://www.ebi.ac.uk/interpro/)^72^ databases for domain and motif search. The reported SNP of all the target genes belonged to different domains and motifs.

#### 2.3.2 3D modeling and free energy deviation calculation of the nsSNPs

For structural analysis of both wild‐type and mutants, 3D structures were generated by the MODELLER software (https://toolkit.tuebingen.mpg.de/#/) from HHpred tools of the Max Planck Institute.^73^ The accuracy of the predicted model was evaluated by using the Ramachandran plot generated by the PROCHECK tool.^74^ Both the wild‐type and mutant structures of 40 GC gene products were then subjected to YASARA (Yet Another Scientific Artificial Reality Application (YASARA) where the YASARA force field was used for energy minimization, enabling the optimization of mutant protein damage. Thus precisely calculates and compares free energy between the wild‐type and the mutant.^75^ Among the products of 40 genes used as input to YASARA, the software was able to predict free energy for 30 of them.

#### 2.3.3 Molecular docking of the target nsSNPs with corresponding drugs

Each SNP target was subjected to a homology search using BLASTp against DrugBank 3.0 target collection.^76^ The targets that had a hit in DrugBank were predicted to be capable of binding to approved, experimental, and available drugs. The target SNPs with available drugs in DrugBank were termed as “Druggable” targets. Targets with non‐hit with DrugBank 3.0 were termed as “Novel” targets. Then, the interacting (.pdb files) drugs with targets were retrieved from the DrugBank 3.0 database. Autodock Vina was employed to analyze the binding affinity of druggable targets for their corresponding drugs^77^ following the preparation of both the SNP targets and drug molecules for docking experiments. The grid box parameter for the docking runs was set in a way that incorporated the SNPs found on a specific region of the targets.

### 2.4 Primer design for in vitro nsSNP‐cancer association confirmation

For in vitro confirmation of the association of selected nsSNPs with GC, primers were designed for both allele‐specific PCR and PCR‐RFLP assay. Allele‐specific PCR is effective for SNP genotyping and mutation detection while PCR‐RFLP assay is convenient for detecting any single nucleotide base change at a specific restriction site. First, respective gene sequences of the damaging nsSNP were extracted from NCBI (https://ncbi.nlm.nih.gov). For the designing of allele‐specific primer, primer3plus (http://www.bioinformatics.nl/cgi-bin/primer3plus/primer3plus.cgi)^78^ and NetPrimer (http://www.premierbiosoft.com/netprimer/) web tools were used. Primer for PCR‐RFLP assay was designed after selecting suitable restriction enzymes that can cut at a specific mutation site of the gene sequence. Two web‐based resources, NEBcutter V2.0 (http://www.labtools.us/nebcutter-v2-0/)^79^ and Webcutter (http://rna.lundberg.gu.se/cutter2/) were utilized to select restriction enzymes for PCR‐RFLP. Both types of primers were designed for wild and mutant gene sequences. Out of the 40 GC genes, the potential allele‐specific primers for 33 genes and PCR‐RFLP primers for 4 genes were designed.

### 2.5 Establishing an experimental validation pipeline for identified polymorphisms

#### 2.5.1 Blood sampling and DNA extraction

Peripheral blood was collected from three cancer patients in EDTA containing vacutainer (BD, Oxford, UK) Tube from Bangabandhu Sheikh Mujib Medical University (BSMMU) Hospital (all international, national, and/or institutional guidelines were followed according to the ethics committee of National Institute of Biotechnology [NIB]; ethical approval number: NIBREC2021‐03). 20 mM Tris–HCl (pH 8.0) was used to lyse erythrocytes of the samples by osmotic shock. DNA was then isolated using a Genomic DNA isolation kit (FavorPrep, Favorgen, Taiwan), and the DNA concentration was determined using a Nanodrop spectrophotometer (Thermo Scientific, USA). Finally, acceptable DNA samples (A260/A280 of 1.8–2.0) were diluted to 50 ng/μL and stored at −80°C until used.

#### 2.5.2 Genotyping of gastric cancer

As the goal of the present study is to establish the experimental workflow, we considered only one polymorphism nsSNP (rs34466743) from CDH1. Selected nsSNP of CDH1 gene was detected by allele‐specific polymerase chain reaction method using the following primers 5′GCCTTATGATTCTCTGCTCG‐3′ (wild‐Type forward), 5′GCCTTATGATTCTCTGCTCA‐3′ (mutant Type forward), and 5′‐AACCACCAGCAACGTGATTT‐3′ (reverse). Briefly, the PCR mixture contained 1× PCR master mix (Thermo Fisher Scientific, Waltham, MA, USA), 10 picomoles of each primer, and 50–100 ng of genomic DNA in a total volume of 25 μL. Amplification was accomplished by following thermal cycling conditions: 95°C for 5 min (one cycle); 95°C for 30 s, 57°C for 20 s, and 72°C for 30 s (30 cycles); 72°C for 7 min (one cycle), and hold at 4°C. After resolving the product along with DNA size markers (1 kb + DNA ladder, Invitrogen, USA) in 2% agarose gel and staining the gel with SYBR Safe DNA gel stain (Thermo Fisher Scientific, USA), the amplification product (241 bp) was visualized and documented using Gel documentation (protiensimple, Santa Cara, CA, USA). This experiment is replicated three times.

#### 2.5.3 DNA sequencing

The two samples which exhibited heterozygous genotypes were subjected to sequencing. The PCR products were purified via the PureLink PCR purification kit (Thermo Fisher Scientific, USA). Then the purified amplicons went under Sanger dideoxy method sequencing. Here, ABI 3500 was used with a BigDye Terminator version 3.1 cycle sequencing kit (Applied Biosystems, USA).

### 2.6 Development of “GasCanBase” database

An HTML5 website was developed using the data generated in the preceding steps of this study. All the information has been subdivided into different tabs by using HTML5. The homepage of the GasCanBase briefly describes the background and method behind the construction of the database. Apart from the homepage, the GasCanBase website also contains the following tabs—cancer‐associated genes, exploration of nsSNPs, functional impact, druggability, and the team. An extra tab has also been provided for other researchers to upload their research data related to GC.

## 3 RESULTS

**FIGURE 1:**
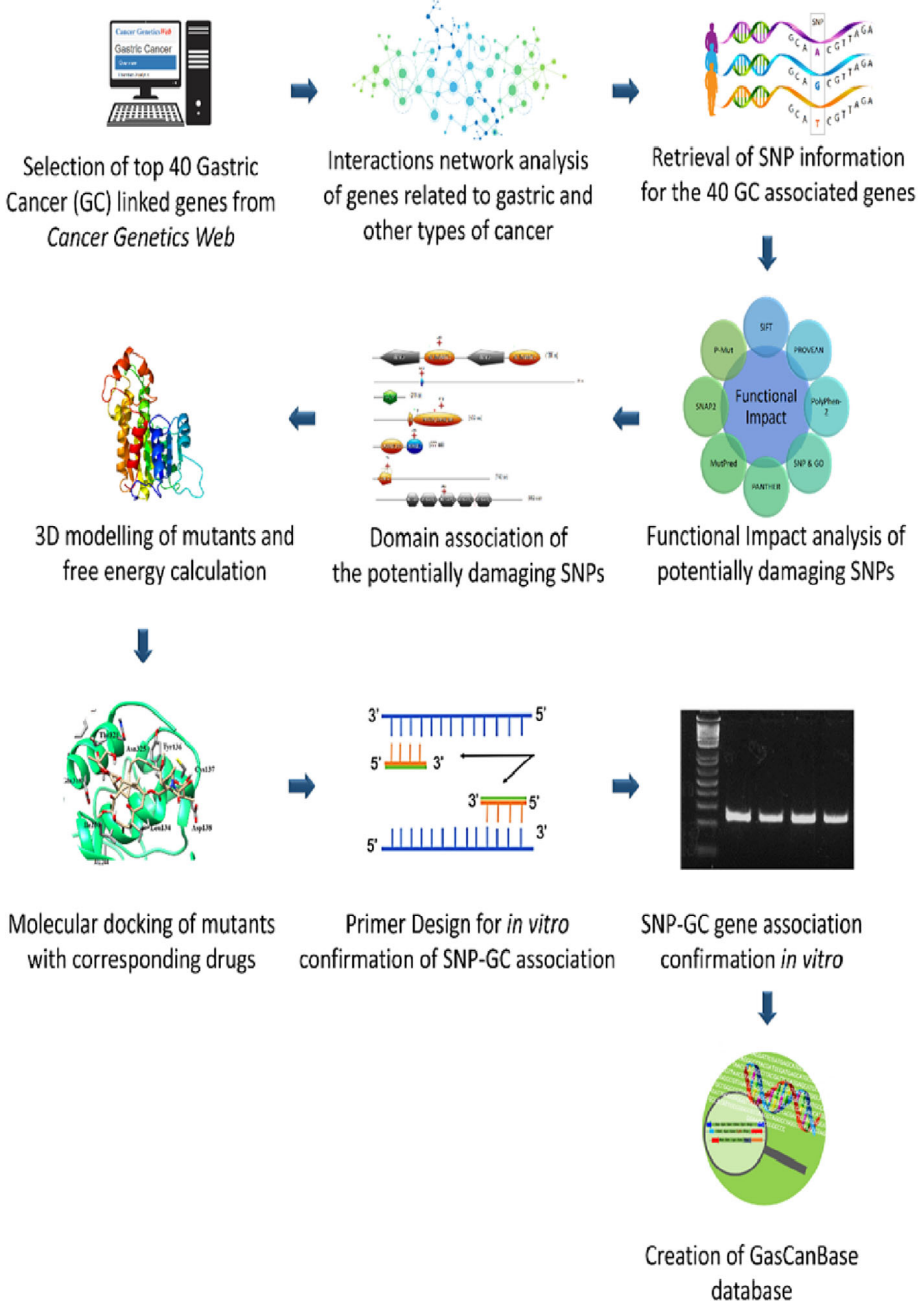
Brief workflow of the study.

The overall workflow of the present study is described in Figure 1.

### 3.1 Exploration of gastric cancer gene interactions network

**FIGURE 2:**
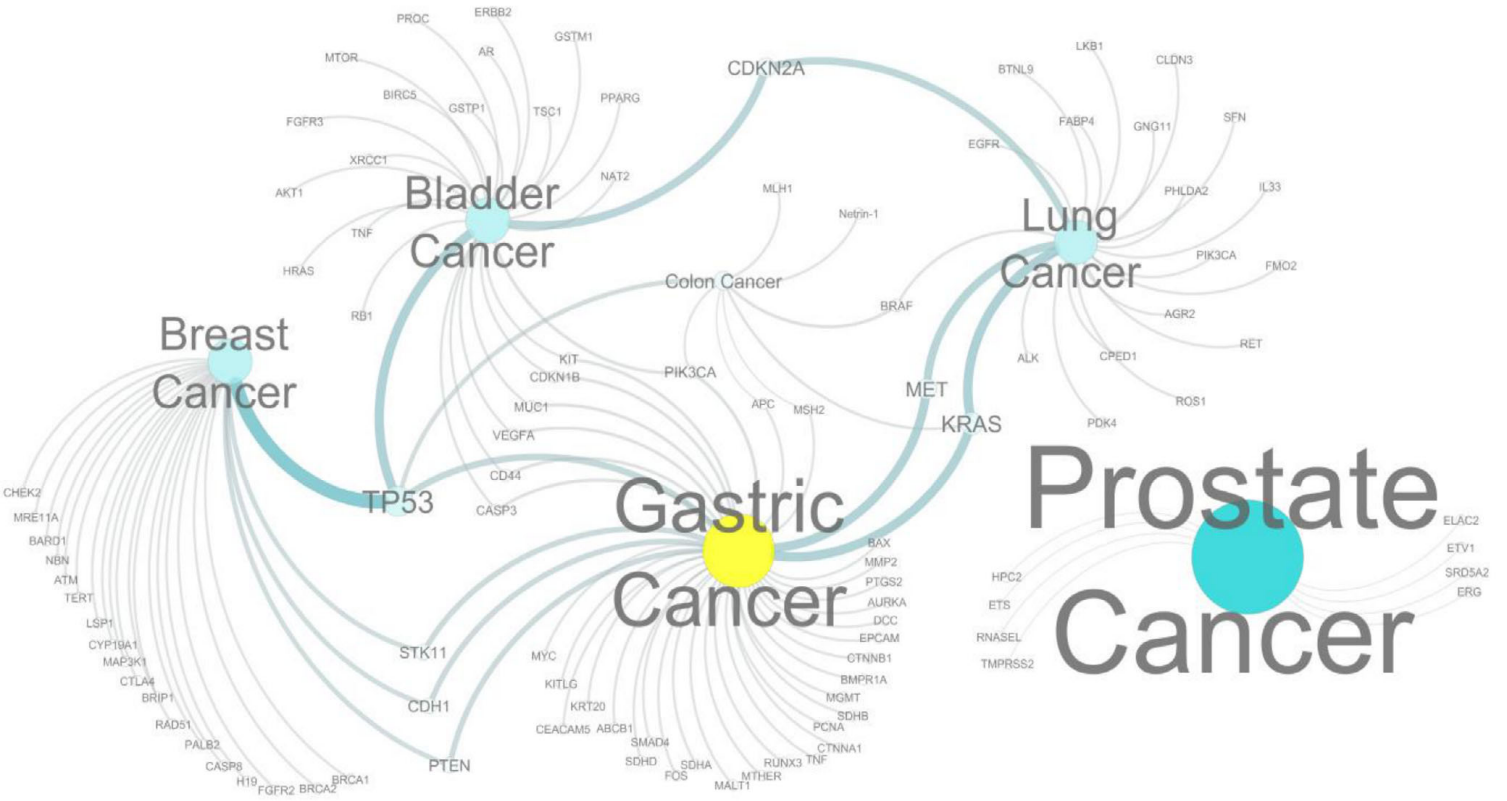
Interactions network of various cancer‐linked genes. The gastric cancer‐associated genes appear to be also associated with breast, bladder, colon, and lung cancer. However, no interactions between gastric and prostate cancer associated genes were recorded.

At first, 40 genes were identified that had an association with gastric carcinogenesis. The genes linked to other cancers such as prostate (8 genes), colon (7 genes), bladder (27 genes), breast (22 genes), and lung cancer (21 genes) were also enlisted (Supplementary Table 1). Afterward, gene interaction networks were analyzed to find out the genes in common between different types of cancers and GC (Figure 2 and Supplementary Table 2). A group of 8 GC‐related genes was found (CASP3, CD44, VEGFA, MUC1, CDKN1B, KIT, PIK3CA, and TP53) which are common with bladder cancer genes. These genes share a common co‐expression and pathway network (Supplementary Figure 1 and Supplementary Table 3). Four genes namely, TP53, STK11, CDH1, and PTEN of GC were also found to be linked with breast cancer (Supplementary Table 2) as well. These four genes interacted with the breast cancer genes in a manner of co‐expression, genetic interactions, and pathway network (Supplementary Figure 2 and Supplementary Table 4). The interaction network revealed that TP53, PIK3CA, APC, MSH2, and KRAS genes are common between GC and colon cancer (Figure 2 and Supplementary Table 2). These genes were observed to interact in a way of co‐expression, genetic interactions, and co‐localization networks (Supplementary Figure 3 and Supplementary Table 5). Only two genes (KRAS and MET) were found to be associated with lung cancer (Figure 2 and Supplementary Table 2). These two GC genes also showed interactions with lung cancer‐related genes in terms of co‐expression, genetic interactions, and pathway network (Supplementary Figure 4 and Supplementary Table 6). Interestingly, it was observed that there was no association between GC genes with prostate cancer genes (Supplementary Table 2). By cross‐referencing with all of the interaction data, 17 GC‐associated genes (CASP3, CD44, VEGFA, MUC1, CDKN1B, KIT, PIK3CA, TP53, STK11, CDH1, PTEN, PIK3CA, APC, MSH2, KRAS, KRAS, and MET) were identified that interacted with the genes related to other types of cancer.

### 3.2 Pinpointing most deleterious nsSNPs of gastric cancer

Most of the SNPs pose no negative consequence on genes' function or physiology. Therefore, it is crucial to uncover whether an nsSNP could affect the protein function and contribute to the disease. The present study is based on identifying nsSNPs of GC‐linked genes which might have a severely deleterious effect on its gene product. Initially, 34 588 nsSNPs were sorted from 1 493 460 SNPs of 40 genes of GC reported in available databases (Supplementary Tables 7–46). Furthermore, 7 tools namely, SIFT, PolyPhen2, PMut, MutPred, SNAP2, SNP&GO, and PANTHER were employed for the selection of nsSNPs with the most deleterious/damaging impact on the respective GC gene (Supplementary Tables 7–46). At the end of this stringent filtering pipeline, only one nsSNP was chosen as the most damaging nsSNP for each gene and assumed to be involved in the alteration of protein function. In total, 11 363 missense nsSNPs were identified located within the 40 GC genes, 474 of whom were predicted to be damaging and 40 to be the most damaging (Supplementary Tables 47–86).

### 3.3 Mutational impact on the gene product

#### 3.3.1 Domain mapping of the nsSNPs

To assess the impact on the gene product of 40 GC genes, the amino acid sequences of the proteins of the corresponding genes were retrieved (Supplementary Text 1). The SNPs in domain regions have been thought to be strong candidates that alter protein functions. It was also investigated whether our identified nsSNPs fell within the domain regions. Our findings suggest that most of the selected nsSNPs were within some domains or motif regions (Supplementary Figure 5). The individual domain IDs, names of the domains, their functions as well and their specific positions within the protein were also addressed (Supplementary Table 87).

#### 3.3.2 Comparative modeling and free energy deviation

BLAST against the Protein Database (PDB) was performed and the structure of the closest related proteins for each protein sequence of corresponding 40 genes was found. The 3D model was built and the quality of each model was checked (Supplementary Figures 6–45). Further, the amino acid (nsSNP) from wild‐type protein sequences was replaced and the quality of the models was assessed. YASARA view mutation tool was used to carry out the mutations (G59S, P62S, L184S, L224P, A276V, L361P, R592H, T595M, and I673T) separately for each of the gene products and it showed a decline in free energy for all the mutant models compared to the wild‐type models (Supplementary Table 88). These results point toward a noteworthy change in the structure of each protein that can demolish its natural function.

#### 3.3.3 Putative effect of nsSNPs on drug binding

Drug binding analysis was also carried out to confirm the structural variation and possible dysfunction of the final product of each 40 GC gene between the wild‐type and mutant model. The drugs against the protein receptor of 40 GC genes were selected using the DrugBank. DrugBank suggested that some drugs were available against the corresponding protein of 10 GC genes (Supplementary Table 89). Thereafter, molecular docking analysis was performed between the suggested drugs and protein receptors of 10 GC genes. Although the same docking area was used for docking runs, eight GC genes were found to interact with varying binding affinities. (Supplementary Table 90). These results confirmed the structural variation and the ineffectiveness of the drugs against the mutant models.

### 3.4 Establishing an experimental validation pipeline

**FIGURE 3:**
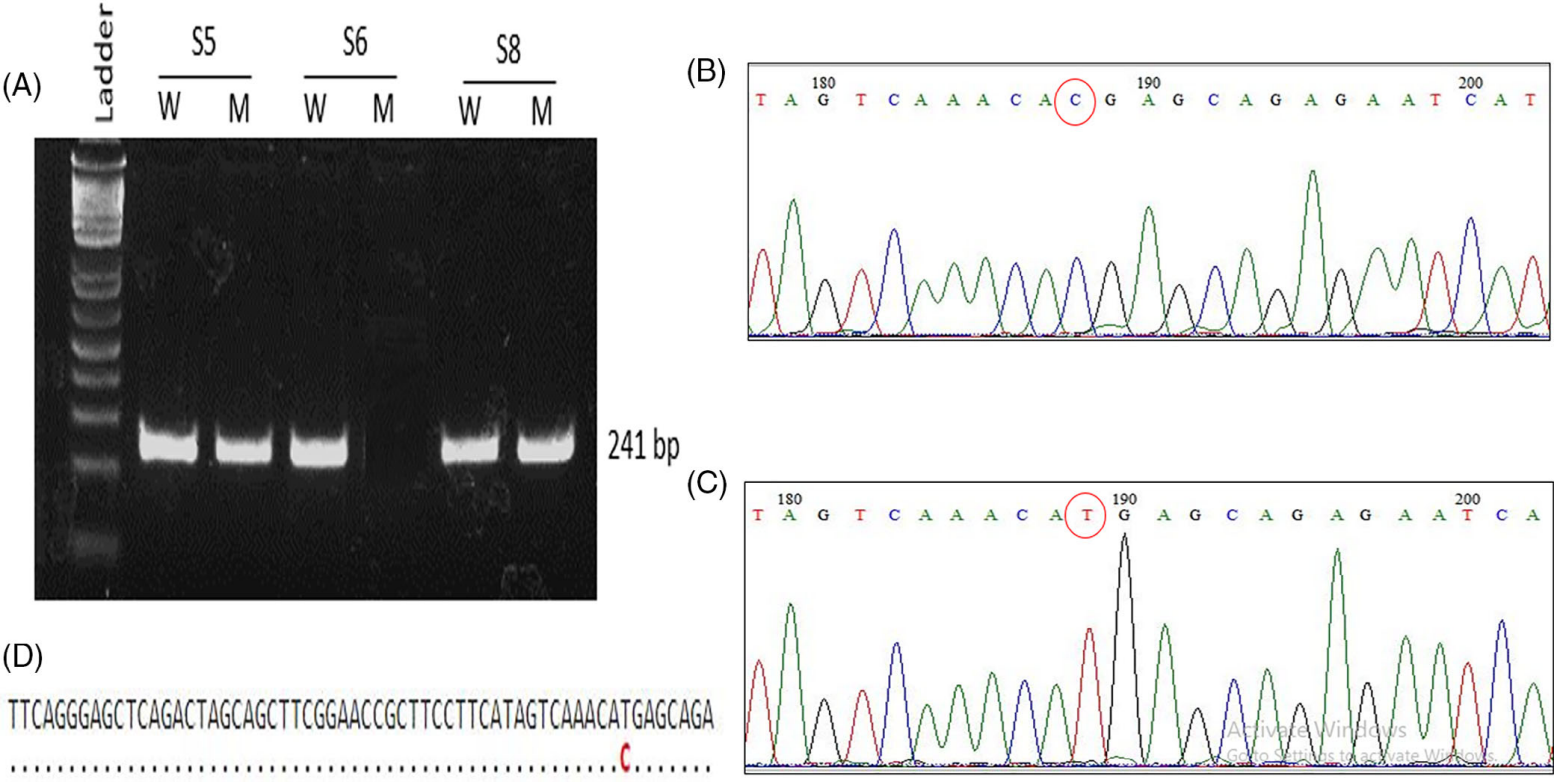
PCR amplification using allele specific primers for rs34466743 of CDH1 gene. (A) These SNPs were screened in different blood samples (S1, S2, and S3) of Gastric Cancer patients. Among the three blood samples, rs34466743 was present in S1 and S3. DNA sequencing reveals the presence of (B) wild type and (C) mutant type nucleotide base. d) DNA sequence of the both wild and mutant type nucleotide base.

The current study also set out to establish a validation pipeline for the identified polymorphisms. Therefore, we selected the CDH1 polymorphism for validation. Later, a primer set was predicted (forward and reverse primer). Allele‐specific primer design and restriction enzyme identification were carried out to facilitate association studies using DNA extraction from human blood samples. The gene sequence (length 500–100 bp) of each gene where the nsSNP was included within this sequence was retrieved (Supplementary Text 2). Small amounts of the genetic material can now be amplified for the identification of mutations in human genes by Polymerase Chain Reaction (PCR). The primers (18–22 bp) were designed for each gene sequence of GC genes meeting all the criteria including primer length, melting temperature, GC content, cross homology, and so forth (Supplementary Tables 5.1–6.15). The similarity was checked for the designed primers against the Human Genome using the BLAST tool. The primer was scrutinized to make sure that there would be no non‐specific amplification. To identify the specific polymorphism (nsSNP) within the respective gene sequence from the PCR product, the most suitable restriction enzymes were also selected (Supplementary Tables 5.1–6.15). However, specific restriction enzymes for each of the 40 GC genes for the specific location of polymorphism were not found. Therefore, allele‐specific primers were also designed through which PCR could confirm the existence of genetic polymorphisms (Supplementary Tables 5.1–6.15). Later, allele‐specific primers (both wild type and mutant) were applied and confirmed the presence of selected nsSNP (rs34466743) from the CDH1 gene in blood samples of cancer patients (Figure 3A). Further, the DNA sequencing also confirmed the mutant allele of the CDH1 gene (Figure 3B–D).

### 3.5 The GasCanBase web interface

**FIGURE 4:**
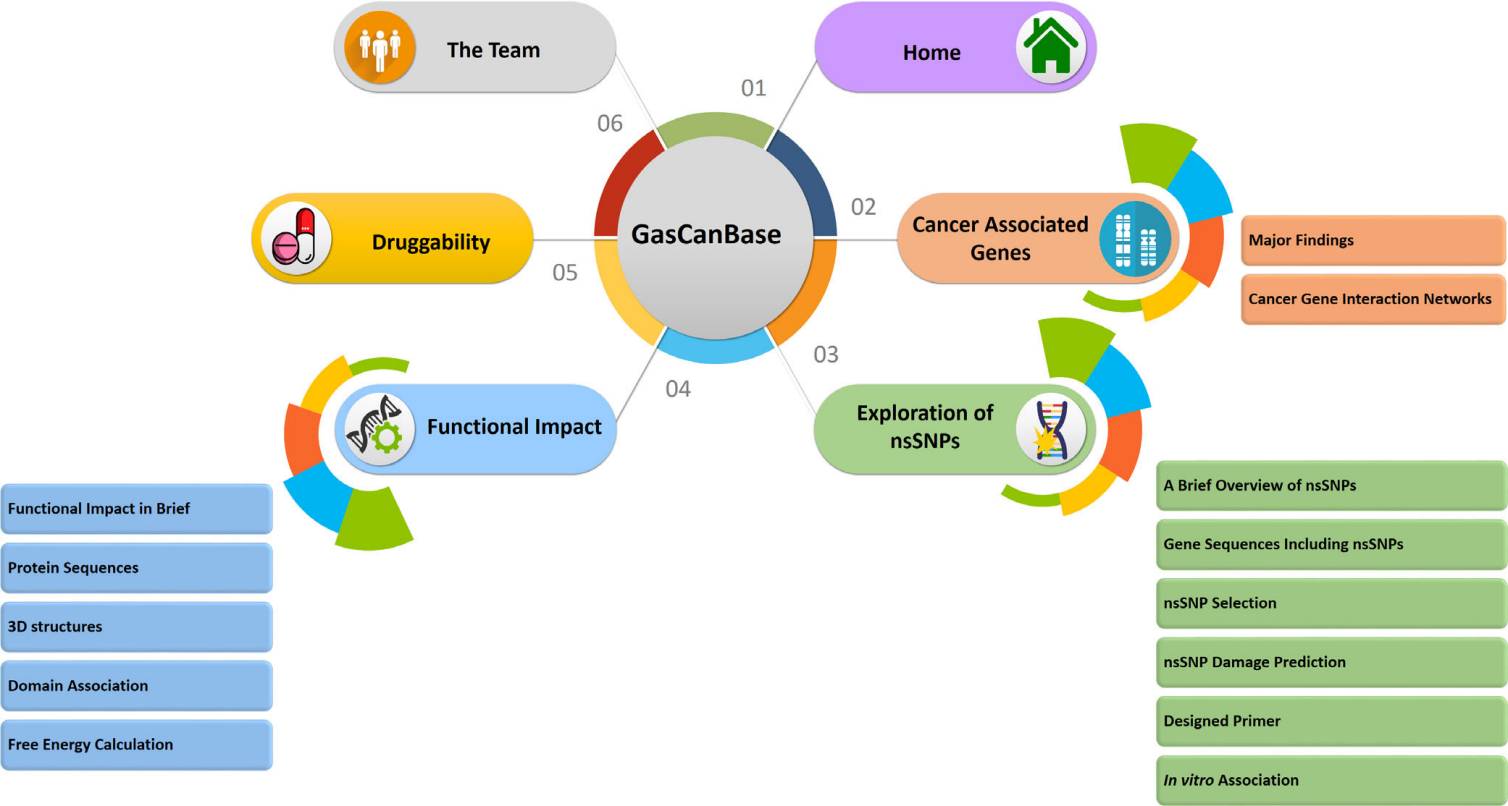
Architecture of GasCanBase database. A wide range of information related to gastric cancer were deposited into the database.

The website was named “GasCanBase” which was an abbreviated form of “Gastric Cancer Database.” It can be accessed at www.gascanbase.com. The website contained five tabs namely‐ home, cancer‐associated genes, exploration of nsSNPs, functional impact, druggability, and the team (Figure 4 and Supplementary Figure 46).

## 4 DISCUSSION

Disease‐causing polymorphisms are the key focus in human genetics to explore disease mechanisms and therapeutics. Nowadays, non‐synonymous SNPs (nsSNPs) are getting huge interest in the scientific community as they can affect the protein structures and their functions by substituting an amino acid (Missense) or introducing a stop codon (Nonsense), both of which ultimately plays an important role in causing the disease. The missense substitutions are the most common damaging nsSNPs associated with many genetic diseases including sickle cell anemia, Tay Sach's disease, hemochromatosis, and so forth.^80^ The identification of nsSNPs which affect the functional activity of a protein and contribute to disease progression is an important task. In silico approach is a useful way to predict the nsSNPs that are most likely to be damaging.^81^ However, the nsSNPs in GC genes have not yet been explored to prioritize the deleterious nsSNP based on their corresponding genes, protein structure, functional association, drug binding analysis as well as population frequency. Therefore, the current study has addressed the novel findings of nsSNPs along with an interactive “GasCanBase” database. GC causes the formation of malignant cells in the lining of the stomach. Abnormal functional activity of various genes is responsible for the development of GC, which is accompanied by severe stomach pain and discomfort.^82^ These abnormal activities of genes could emerge from mutations and are likely to be associated with the disease. In this study, 40 genes that have been documented in the literature and cancer hub having a direct or indirect role in the development of GC were studied. Additionally, an examination was conducted on the network of GC genes, which consists of 40 genes. The interactions between these genes and other genes with prostate (*n* = 8), colon (*n* = 7), bladder (*n* = 27), breast (*n* = 22), and lung cancer (*n* = 21) (Supplementary Table 1) were investigated. The Protein–protein interaction (PPI) network suggests that 17 GC‐related genes interact with other types of cancer in various ways (Figure 2, Supplementary Tables 1–4 and Supplementary Tables 1–6). These 17 genes are of great interest in medical science as they are common in several types of cancer. From a vast number of SNPs (*n* = 1 493 460) only the nsSNPs (*n* = 34 588) of 40 genes were selected. Using seven different computational algorithms, only 40 nsSNPs out of 11 359 might exert deleterious effects on the structure, function, solubility, or stability of their respective proteins (Supplementary Tables 7–86). These 40 genes were then analyzed further. The current study also explores the mechanisms by which SNPs belonging to certain protein domains in each of the 40 genes could potentially contribute to the disease. Protein domains play a significant role in cell signaling networks by binding with upstream and downstream proteins.^4^, ^36^ Proteins with domain‐altering nsSNPs might disturb certain cell signaling pathways and cause numerous biological dysfunctions. Most of the selected nsSNPs from our study fall within the functional unit of certain domains and motif regions (Supplementary Text 1, Supplementary Figure 5, Supplementary Table 87). In silico analysis was performed for the selected nsSNPs of each of the 40 genes linked to GC to probe the deleterious effect on the 3D structure of their corresponding proteins as nsSNP with the potential to cause structural modifications through amino acid substitutions could give rise to functional abnormalities. First, 3D structures of both wild‐type and mutant proteins were generated and their quality was found to be satisfactory (Supplementary Figures 6–45). The free energy of both wild‐type and mutant structures was calculated to gain insight into their structural conformations. The variations in free energy suggested the structural instability of the mutant models. To confirm structural instabilities due to nsSNPs, the current study also focused on drug binding analysis using molecular docking. For this purpose, drugs against the GC genes were screened and selected. The nsSNPs that failed to have a matching hit in the DrugBank database (https://go.drugbank.com/) were termed as “Novel” targets (Supplementary Figures 89 and 90). Then molecular docking was performed to observe the effect of the nsSNPs change on the protein structural conformations. The same docking protocol, the same receptor area for docking runs, and the same drug were used for both the wild‐type and the mutant 3D models. However, the docking result showed different binding affinities and different interacting residues. These results confirmed that only a single amino acid substitution has changed the structural conformations and as a result, the drugs could not interact with the same binding site in the same manner (Supplementary Figures 89 and 90). So, the current study provides strong evidence that the selected nsSNPs might prompt deleterious effects as they have been computationally proven to alter the protein domains and motifs. They are also capable of changing structural conformations and interfering with drug binding.^83^ Since these nsSNPs could have a damaging impact with respect to their protein structure, they might be used as potential genetic markers.

### 4.1 Establishing an experimental pipeline for identified polymorphisms

The present study has tried to establish an experimental validation pipeline for the selected 40 GC nsSNPs (Supplementary Text 2). Therefore, we performed wet lab validation on CDH1 gene SNP to establish an experimental workflow. We only tested the presence of the selected nsSNP (rs34466743) from the CDH1 gene in GC patients which made the pipeline for further experimental validation. To achieve this, allele‐specific primers were applied to facilitate association studies using GC‐affected human samples, and the selected nsSNP of the GC gene (CDH1) was found in the PCR products (Figure 3A). Later, DNA sequencing revealed the presence of a mutation in the CDH1 gene (Figure 3B–D). As the presence of the selected nsSNP (rs34466743) from the CDH1 gene was confirmed in the blood samples of GC patients, more samples could reveal information about the frequency of this polymorphism that is required to develop a disease prognostic biomarker. Furthermore, it is important to take into account that germline variations can significantly impact the risk of cancer linked with a germline mutation.^84^, ^85^, ^86^ In addition, an increasing number of germline variations have been shown to affect protein function, even when there is no observable association with disease risk. Some of these germline modifications have been identified in cancer‐related genes.^87^, ^88^, ^89^ In a recent study, an examination of genetic information obtained from almost 7000 individuals enrolled in the Cancer Genome Atlas (TCGA) revealed the presence of roughly 400 germline single nucleotide polymorphisms (SNPs) that exhibit a notable correlation with distinct cancer categories when compared to the remaining cancer kinds.^90^ Similarly, a vast number of samples could be employed to validate these SNPs since the mutational impact of even the primers (PCR‐RFLP) and allele‐specific primers have already been mentioned for each SNPs in the developed database. Moreover, the established pipeline can be followed for in vitro association studies (Figure 3). However, the metadata must be collected from the patients and then analyzed statistically to find out the variance among the populations.

Furthermore, an interactive database “GasCanBase (www.gascanbase.com)” was developed. Here, SNP data of 40 GC genes is available along with a wide range of information (Figure 4). The research findings regarding GC with their associated genes have been stored in this database. The developed database has been enriched with multi‐dimensional information regarding the SNPs. It will be regularly updated with the most recent data of GC. Public users will also be able to upload their research data into this database. Therefore, the database could be exploited for further research in GC.

### 4.2 Limitations of the study

The current study has limitations in terms of the experimental validation of all the identified 40 GC genes. In this study, only one polymorphism has been validated, demonstrating that the remaining 39 polymorphisms may also be validated. The purpose of validating a polymorphism is to establish an experimental workflow so that other researchers can validate the rest of the 39 polymorphisms by following our experimental workflow and even utilizing our primers from the GasCanBase database.

## 5 CONCLUSION

This study scrutinizes a vast SNP pool of 40 GC genes in order to unveil their deleterious effects that contributes to the disease. Subsequent association studies and the development of an easily accessible interactive database (GasCanBase) will enrich the identification of biomarkers, therapeutic targets, drug discovery, and the integration of personalized medicine into GC treatment.

## AUTHOR CONTRIBUTIONS

**Mohammad Uzzal Hossain:** Investigation (equal); methodology (equal); visualization (equal); writing – original draft (equal); writing – review and editing (equal). **Ishtiaque Ahammad:** Investigation (equal); methodology (equal); visualization (equal); writing – original draft (equal); writing – review and editing (equal). **Md. Moniruzzaman:** Investigation (equal); Methodology (equal). **Mahbuba Akter Lubna:** Formal analysis (equal). **Arittra Bhattacharjee:** Writing – review and editing (equal). **Zeshan Mahmud Chowdhury:** Writing – review and editing (equal). **Istiak Ahmed:** Formal analysis (equal). **Md. Billal Hosen:** Formal analysis (equal). **Shourov Biswas:** Formal analysis (equal). **Keshob Chandra Das:** Methodology (equal); resources (equal); supervision (equal). **Chaman Ara Keya:** Supervision (equal); writing – review and editing (equal). **Md. Salimullah:** Conceptualization (equal); supervision (equal); writing – review and editing (equal).

## CONFLICT OF INTEREST STATEMENT

The authors have stated explicitly that there are no conflicts of interest in connection with this article.

## Supporting information


**Data S1** Supporting Information.

## Data Availability

All the data generated in this study is included in the manuscript and the supplementary files. GasCanBase is available at: https://www.gascanbase.com/.
