# Supplementary material for: Investigation of pathogenic germline variants in gastric cancer and development of “GasCanBase” database
Source: Cancer Rep (Hoboken). 2023 Oct 22;6(12):e1906. doi: 10.1002/cnr2.1906 (PMC10728505; doi:10.1002/cnr2.1906)
Supplement: Supplementary file 1 — Data S1 Supporting Information. [file CNR2-6-e1906-s001.zip › Supplementary File/Figure S17. 3D model structure and validation of CTNNB1 gene.pptx]

## Slide 1
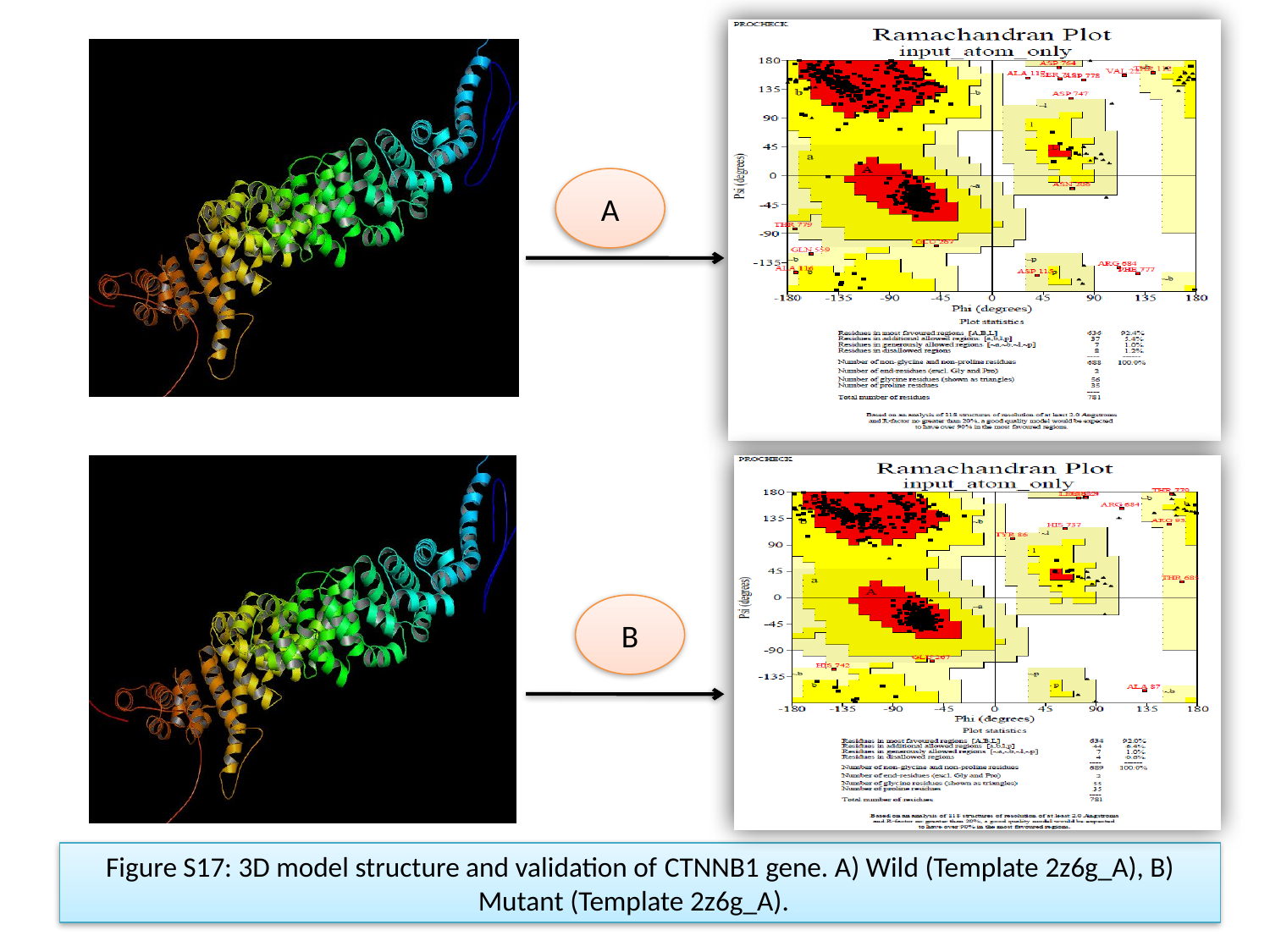

A
B
Figure S17: 3D model structure and validation of CTNNB1 gene. A) Wild (Template 2z6g_A), B) Mutant (Template 2z6g_A).
