# Supplementary material for: Investigation of pathogenic germline variants in gastric cancer and development of “GasCanBase” database
Source: Cancer Rep (Hoboken). 2023 Oct 22;6(12):e1906. doi: 10.1002/cnr2.1906 (PMC10728505; doi:10.1002/cnr2.1906)
Supplement: Supplementary file 1 — Data S1 Supporting Information. [file CNR2-6-e1906-s001.zip › Supplementary File/Figure S5. SNP in domain region of cancer gene.pptx]

## Slide 1
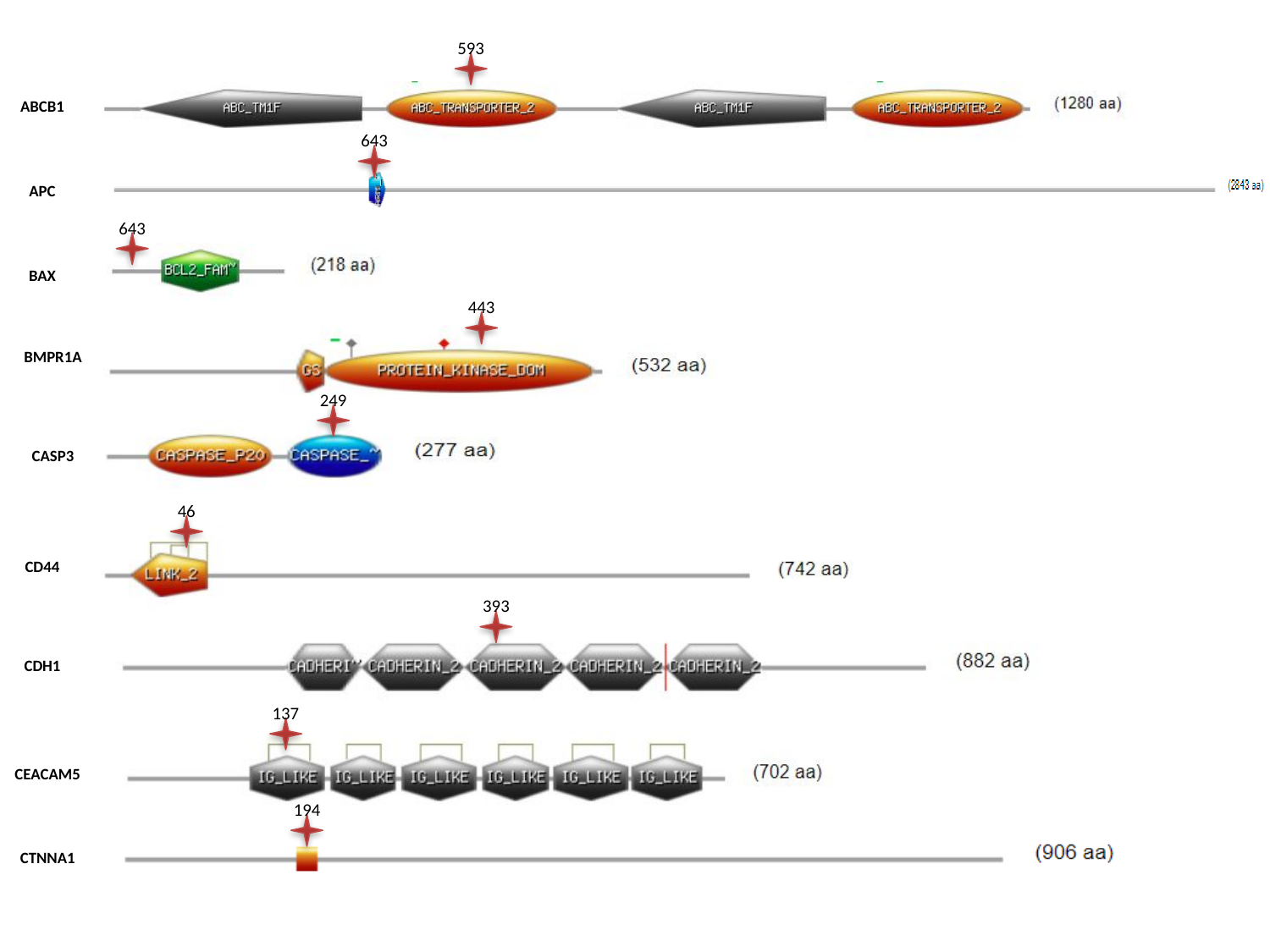

593
ABCB1
643
APC
643
BAX
443
BMPR1A
249
CASP3
46
CD44
393
	CDH1
137
	CEACAM5
194
CTNNA1

## Slide 2
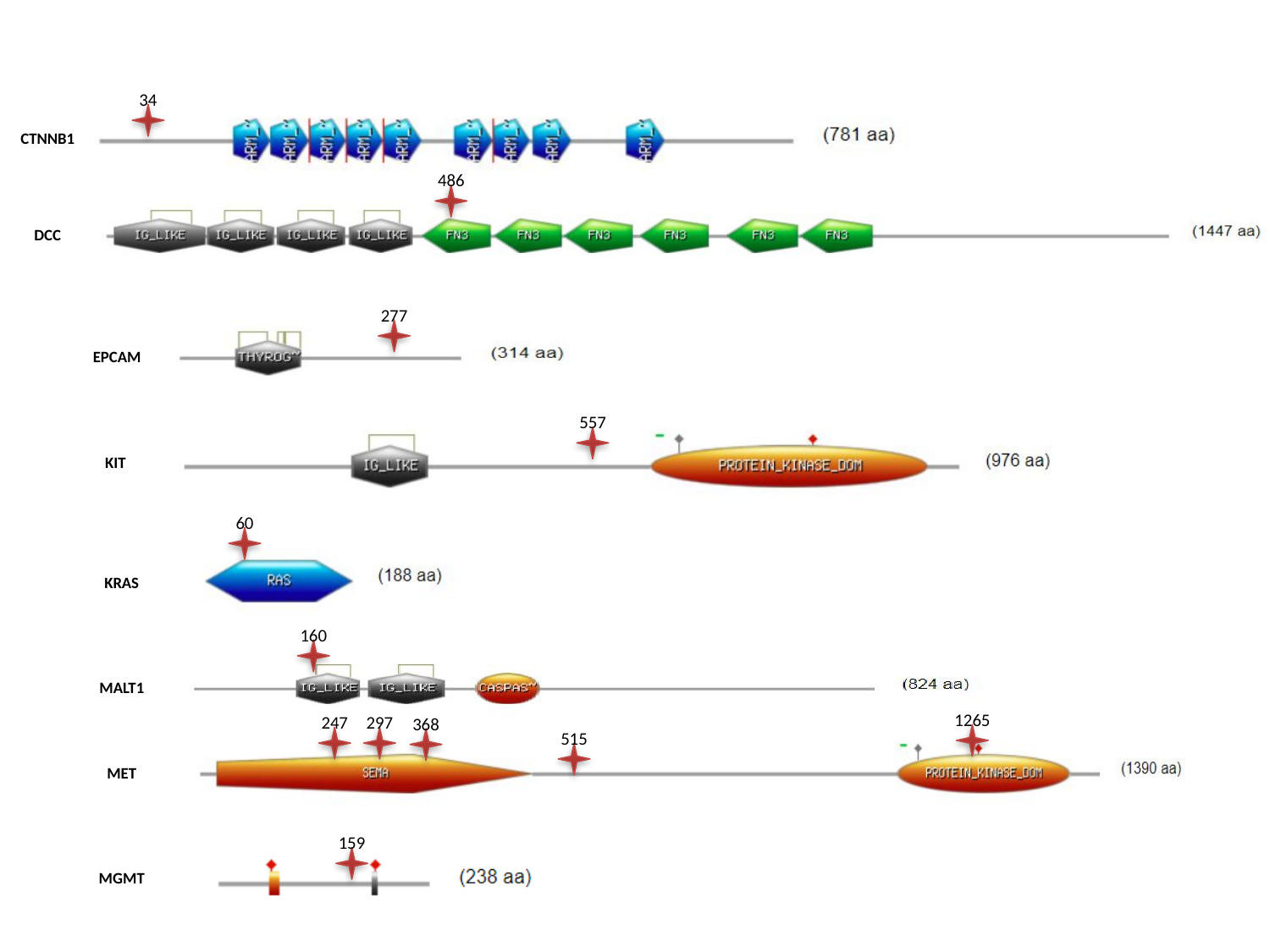

34
CTNNB1
486
DCC
277
EPCAM
557
KIT
60
KRAS
160
MALT1
1265
247
297
368
515
MET
159
MGMT

## Slide 3
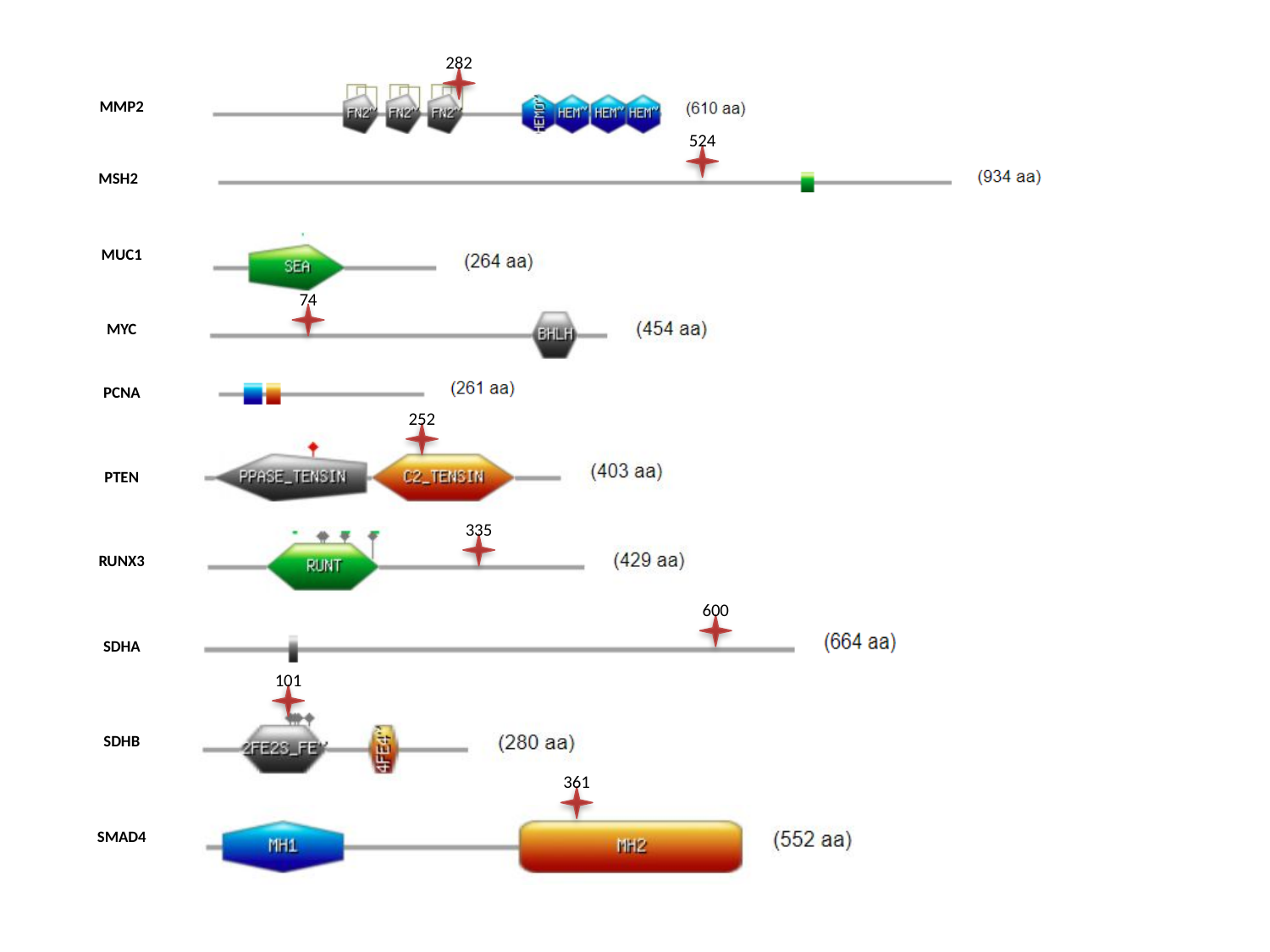

282
MMP2
524
MSH2
MUC1
74
MYC
PCNA
252
PTEN
335
RUNX3
600
SDHA
101
SDHB
361
SMAD4

## Slide 4
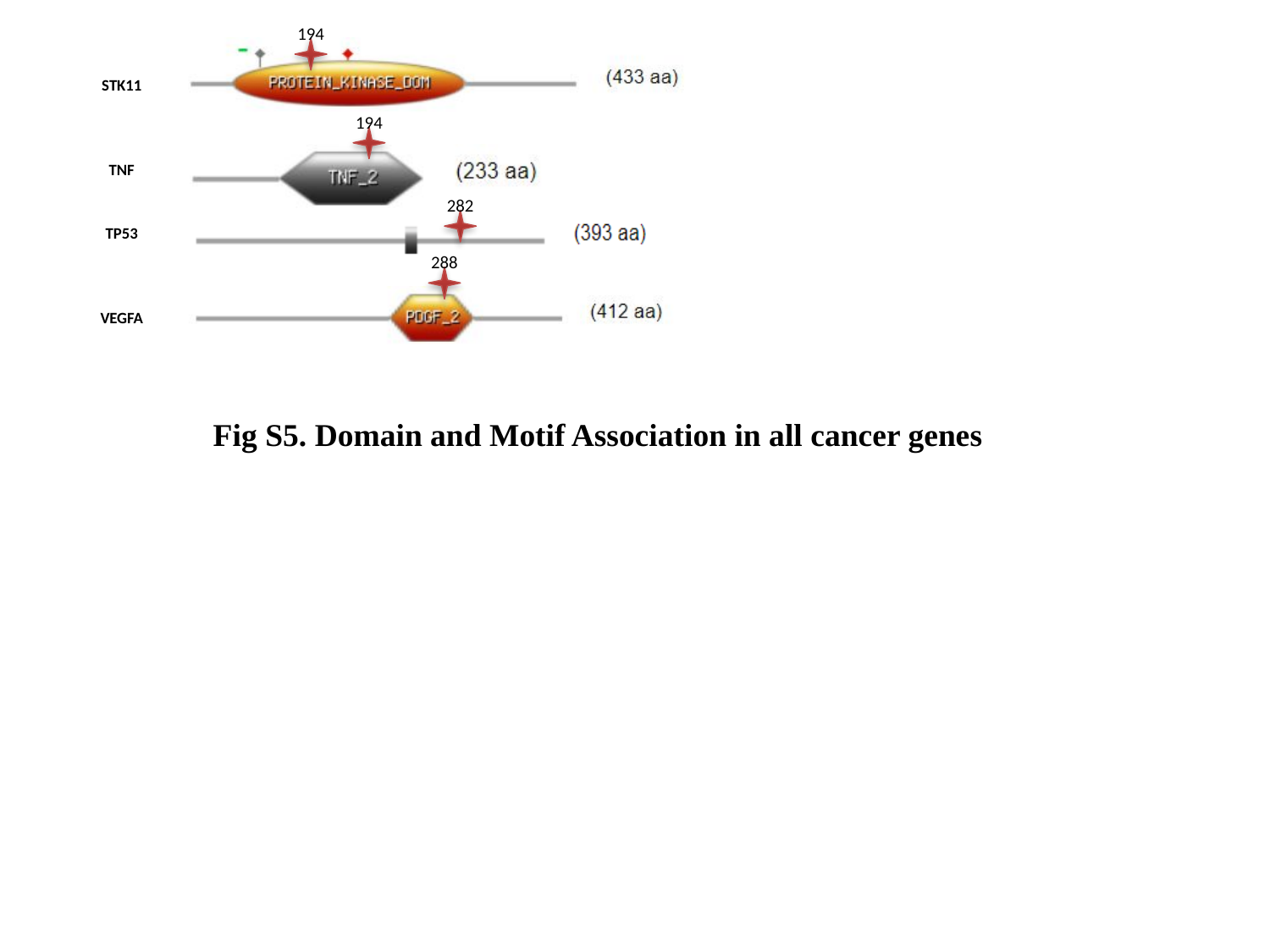

194
STK11
194
TNF
282
TP53
288
VEGFA
Fig S5. Domain and Motif Association in all cancer genes
