# Supplementary material for: Investigation of pathogenic germline variants in gastric cancer and development of “GasCanBase” database
Source: Cancer Rep (Hoboken). 2023 Oct 22;6(12):e1906. doi: 10.1002/cnr2.1906 (PMC10728505; doi:10.1002/cnr2.1906)
Supplement: Supplementary file 1 — Data S1 Supporting Information. [file CNR2-6-e1906-s001.zip › Supplementary File/Table S3. Gene networking of Gastric and Bladder Cancer genes.docx]

**Table S3: Gene networking of Gastric and Bladder Cancer genes**

| **Gene 1** | **Gene 2** | **Weight** | **Network group** |
| --- | --- | --- | --- |
| CASP3 | MUC1 | 0.018755585 | Co-expression |
| SH2B3 | KIT | 0.015913727 | Co-expression |
| UBE2J1 | CASP3 | 0.011887888 | Co-expression |
| PTPN6 | MUC1 | 0.014106806 | Co-expression |
| KIT | CD44 | 0.02083454 | Co-expression |
| GAB2 | KIT | 0.00983172 | Co-expression |
| UBE2J1 | CD44 | 0.018599423 | Co-expression |
| ARHGAP44 | KIT | 0.025426475 | Co-expression |
| KIT | CD44 | 0.015035181 | Co-expression |
| MYB | CD44 | 0.02499112 | Co-expression |
| MYB | KIT | 0.019444807 | Co-expression |
| EGFR | CD44 | 0.012705625 | Co-expression |
| EGFR | KIT | 0.016906707 | Co-expression |
| UBE2J1 | CASP3 | 0.026733134 | Co-expression |
| CASP3 | CDKN1B | 0.013243361 | Co-expression |
| UBE2J1 | CASP3 | 0.006141765 | Co-expression |
| VEGFA | CD44 | 0.012055981 | Co-expression |
| RFWD2 | MUC1 | 0.007574342 | Co-expression |
| MYB | CDKN1B | 0.0222301 | Co-expression |
| EGFR | CD44 | 0.008750701 | Co-expression |
| UBE2J1 | PIK3CA | 0.016668025 | Co-expression |
| CASP3 | PIK3CA | 0.008048266 | Co-expression |
| UBE2J1 | PIK3CA | 0.01224415 | Co-expression |
| UBE2J1 | CASP3 | 0.008411814 | Co-expression |
| PTPN6 | MUC1 | 0.015337558 | Co-expression |
| KIT | CDKN1B | 0.004205926 | Co-expression |
| ARHGAP44 | CDKN1B | 0.004692758 | Co-expression |
| KIT | PIK3CA | 0.08392231 | Pathway |
| RFWD2 | TP53 | 0.64359426 | Pathway |
| APPL1 | CASP3 | 0.48969373 | Pathway |
| DIABLO | CASP3 | 0.4451173 | Pathway |
| SIN3A | TP53 | 0.3628395 | Pathway |
| MYB | TP53 | 0.3628395 | Pathway |
| SOCS6 | KIT | 0.34526947 | Pathway |
| SOCS1 | KIT | 0.34526947 | Pathway |
| GAB2 | KIT | 0.34526947 | Pathway |
| SH2B3 | KIT | 0.34526947 | Pathway |
| PTPRU | KIT | 0.34526947 | Pathway |
