# Supplementary material for: Investigation of pathogenic germline variants in gastric cancer and development of “GasCanBase” database
Source: Cancer Rep (Hoboken). 2023 Oct 22;6(12):e1906. doi: 10.1002/cnr2.1906 (PMC10728505; doi:10.1002/cnr2.1906)
Supplement: Supplementary file 1 — Data S1 Supporting Information. [file CNR2-6-e1906-s001.zip › Supplementary File/Table S4. Gene networking of Gastric and Breast Cancer genes.docx]

**Table S4. Gene networking of Gastric and Breast Cancer genes**

| **Gene 1** | **Gene 2** | **Weight** | **Network group** |
| --- | --- | --- | --- |
| KLRG1 | TP53 | 0.014439655 | Co-expression |
| CAB39 | PTEN | 0.014294511 | Co-expression |
| CTNND1 | CDH1 | 0.015978891 | Co-expression |
| PRKAA2 | PTEN | 0.024588061 | Co-expression |
| TP53 | STK11 | 0.015240385 | Co-expression |
| PRKAB2 | PTEN | 0.009961648 | Co-expression |
| CTNND1 | TP53 | 0.016801013 | Co-expression |
| CTNND1 | TP53 | 0.009371896 | Co-expression |
| PRKAA2 | TP53 | 0.019647276 | Co-expression |
| CTNND1 | CDH1 | 0.008755496 | Co-expression |
| PDGFRB | PTEN | 0.008491529 | Co-expression |
| CENPC | PTEN | 0.008898958 | Co-expression |
| RFWD2 | CDH1 | 0.00843504 | Co-expression |
| CTNND1 | PTEN | 0.024302976 | Co-localization |
| CTNND1 | CDH1 | 0.016944114 | Co-localization |
| STRADA | STK11 | 0.5287121 | Co-localization |
| CAB39 | STK11 | 0.5287121 | Co-localization |
| PRKAA2 | STK11 | 0.5287121 | Co-localization |
| PDGFRB | PTEN | 0.661236 | Co-localization |
| EGFR | CDH1 | 0.1381843 | Co-localization |
| MDM2 | TP53 | 0.06781394 | Co-localization |
| MDM2 | TP53 | 0.08127491 | Genetic Interactions |
| TP53 | PTEN | 0.003375156 | Pathway |
| KLRG1 | CDH1 | 0.03488342 | Pathway |
| STRADA | STK11 | 0.098768964 | Pathway |
| CAB39 | STK11 | 0.098768964 | Pathway |
| PRKAA2 | STK11 | 0.0447614 | Pathway |
| RFWD2 | TP53 | 0.029489465 | Pathway |
| PDGFRB | PTEN | 0.012338694 | Pathway |
| PRKAG2 | STK11 | 0.046882823 | Pathway |
| PRKAB2 | STK11 | 0.04562669 | Pathway |
| CTNND1 | CDH1 | 0.010533359 | Pathway |
| EGFR | TP53 | 0.001220029 | Pathway |
| MDM2 | TP53 | 0.004743363 | Pathway |
| HOXD9 | CDH1 | 0.20802338 | Pathway |
| CAB39L | STK11 | 0.098768964 | Pathway |
| STRADB | STK11 | 0.098768964 | Pathway |
