# Supplementary material for: Investigation of pathogenic germline variants in gastric cancer and development of “GasCanBase” database
Source: Cancer Rep (Hoboken). 2023 Oct 22;6(12):e1906. doi: 10.1002/cnr2.1906 (PMC10728505; doi:10.1002/cnr2.1906)
Supplement: Supplementary file 1 — Data S1 Supporting Information. [file CNR2-6-e1906-s001.zip › Supplementary File/Table S47. Prediction of damaging effect on ABCB1.docx]

| **SNP** | **Protein ID** | **Amino Acid** | **Amino acid change** | **SIFT** | **PolyPhen2** | **PMut** | **MutPred** | **SNAP2** | **SNP&GO** | **PANTHER** |
| --- | --- | --- | --- | --- | --- | --- | --- | --- | --- | --- |
| rs28381902 | NP_000918 | 1280 | E566K | DAMAGING | Probably Damaging | 0.6521  PATHOLOGICAL | 0.388 | Effect91% | Disease | Probably Damaging |
| rs41316450 | NP_000918 | 1280 | I736K | DAMAGING | Probably Damaging | 0.9607 PATHOLOGICAL | 0.820 | Effect 80% | Disease | Probably Damaging |
| rs56107566 | NP_000918 | 1280 | R593H | DAMAGING | Probably Damaging | 0.5236 PATHOLOGICAL | 0.606 | Effect 91% | Disease | Probably Damaging |
