# Supplementary material for: Investigation of pathogenic germline variants in gastric cancer and development of “GasCanBase” database
Source: Cancer Rep (Hoboken). 2023 Oct 22;6(12):e1906. doi: 10.1002/cnr2.1906 (PMC10728505; doi:10.1002/cnr2.1906)
Supplement: Supplementary file 1 — Data S1 Supporting Information. [file CNR2-6-e1906-s001.zip › Supplementary File/Table S48. Prediction of damaging effect on APC.docx]

Table S48. Prediction of damaging effect on APC

| **SNP** | **Protein ID** | **Amino acid** | **Amino acid change** | **SIFT** | **PolyPhen2** | **PMut** | **MutPred** | **SNAP2** | **SNP&GO** | **PANTHER** |
| --- | --- | --- | --- | --- | --- | --- | --- | --- | --- | --- |
| rs1801155 | NP_000029 | 2843 | I1307K | Damaging | Benign | 0.8589 Pathological | 0.127 | Neutral | Neutral | Probably Benign |
| rs4987109 | NP_000029 | 2843 | S1973T | Damaging | Probably Damaging | Neutral | 0.199 | Neutral | Neutral | Probably Damaging |
| rs72541816 | NP_000029 | 2843 | S2621C | Damaging | Possibly Damaging | 0.8076 Pathological | 0.254 | Neutral | Neutral | Probably Benign |
| rs34157245 | NP_000029 | 2843 | R1882T | Damaging | Benign | 0.8870 Pathological | 0.228 | Neutral | Neutral | Probably Damaging |
| rs72541813 | NP_000029 | 2843 | R1589G | Damaging | Possibly Damaging | 0.6492 Pathological | 0.434 | Effect 75% | Neutral | Probably Damaging |
| rs73220015 | NP_000029 | 2843 | E1540Q | Damaging | Probably Damaging | Neutral | 0.132 | Neutral | Neutral | Probably Damaging |
| rs74535574 | NP_000029 | 2843 | Q1429K | Damaging | Probably Damaging | Neutral | 0.262 | Neutral | Neutral | Probably Damaging |
| rs74561014 | NP_000029 | 2843 | G974C | Damaging | Possibly Damaging | 0.8118 Pathological | 0.323 | Effect 59% | Neutral | Probably Damaging |
| rs74727182 | NP_000029 | 2843 | S62F | Damaging | Possibly Damaging | 0.5680 Pathological | 0.257 | Effect 85% | Neutral | Probably Damaging |
| rs75117039 | NP_000029 | 2843 | I638V | Damaging | Possibly Damaging | Neutral | 0.497 | Effect 66% | Neutral | Probably Damaging |
| rs75207119 | NP_000029 | 2843 | S2350F | Damaging | Probably Damaging | 0.8567 Pathological | 0.498 | Effect 71% | Neutral | Probably Damaging |
| rs75207119 | NP_000029 | 2843 | S2350Y | Damaging | Probably Damaging | 0.9078 Pathological | 0.506 | Effect 75% | Neutral | probably damaging |
| rs75239284 | NP_000029 | 2843 | Q999K | Damaging | Benign | 0.6584 Pathological | 0.345 | Neutral | Neutral | possibly damaging |
| rs75870842 | NP_000029 | 2843 | S535F | Damaging | Probably Damaging | 0.7404 Pathological | 0.577 | Effect 71% | Neutral | probably damaging |
| rs76306073 | NP_000029 | 2843 | D1841Y | Damaging | Probably Damaging | 0.9441 Pathological | 0.619 | Effect 71% | Disease | probably damaging |
| rs77056664 | NP_000029 | 2843 | Q1256K | Damaging | Probably Damaging | Neutral | 0.583 | Neutral | Neutral | probably damaging |
| rs77451514 | NP_000029 | 2843 | L505F | Damaging | Probably Damaging | Neutral | 0.570 | Effect 85% | Neutral | probably damaging |
| rs77907679 | NP_000029 | 2843 | E425G | Damaging | Possibly Damaging | Neutral | 0.403 | Effect 71% | Neutral | probably damaging |
| rs78176192 | NP_000029 | 2843 | S2710R | Damaging | Benign | 0.7337 Pathological | 0.443 | Effect 59% | Neutral | probably benign |
| rs78349383 | NP_000029 | 2843 | S643P | Damaging | Probably Damaging | Neutral | 0.863 | Effect 91% | Disease | probably damaging |
| rs79853077 | NP_000029 | 2843 | D802Y | Damaging | Probably Damaging | Pathological | 0.290 | Effect 71% | Disease | probably damaging |
| rs111423620 | NP_000029 | 2843 | D556V | Damaging | Possibly Damaging | Neutral | 0.539 | Effect 85% | Disease | probably damaging |
| rs113486158 | NP_000029 | 2843 | A2648E | Damaging | Possibly Damaging | 0.7830 Pathological | 0.381 | Effect 85% | Neutral | probably damaging |
| rs112961968 | NP_000029 | 2843 | E1464G | Damaging | Benign | 0.5190 Pathological | 0.291 | Effect 63% | Neutral | possibly damaging |
| rs80277939 | NP_000029 | 2843 | M2713L | Damaging | Benign | Neutral | 0.467 | Neutral | Neutral | probably benign |
| rs121918142 | NP_000303 | 461 | W444C | Damaging | Probably Damaging | 0.9450 Pathological | 0.978 | Effect 75% | Disease | Cannot score substitution |
| rs121918143 | NP_000303 | 461 | R211W | Damaging | Possibly Damaging | 0.6951 Pathological | 0.885 | Effect 95% | Disease | Cannot score substitution |
| rs121918144 | NP_000303 | 461 | A301V | Damaging | Probably Damaging | Neutral | 0.965 | Neutral | Disease | Cannot score substitution |
| rs121918147 | NP_000303 | 461 | G343S | Damaging | Probably Damaging | 0.5339 Pathological | 0.981 | Effect 85% | Disease | Cannot score substitution |
| rs121918148 | NP_000303 | 461 | E62A | Damaging | Probably Damaging | 0.6960 Pathological | 0.952 | Effect 80% | Disease | Cannot score substitution |
| rs121918151 | NP_000303 | 461 | P289L | Damaging | Probably Damaging | 0.8186 Pathological | 0.929 | Effect 66% | Disease | Cannot score substitution |
| rs121918154 | NP_000303 | 461 | R272C | Damaging | Probably Damaging | 0.9171 Pathological | 0.853 | Effect 66% | Disease | Cannot score substitution |
| rs121918155 | NP_000303 | 461 | Q226H | Damaging | Probably Damaging | Neutral | 0.975 | Effect 95% | Neutral | Cannot score substitution |
| rs121918156 | NP_000303 | 461 | L265F | Damaging | Possibly Damaging | Neutral | 0.876 | Effect 63% | Neutral | Cannot score substitution |
| rs121918157 | NP_000303 | 461 | I445M | Damaging | Possibly Damaging | Neutral | 0.935 | Effect 91% | Neutral | Cannot score substitution |
| rs121918158 | NP_000303 | 461 | V339M | Damaging | Probably Damaging | Neutral | 0.936 | Effect 85% | Neutral | Cannot score substitution |
| rs121918159 | NP_000303 | 461 | H149P | Damaging | Probably Damaging | Neutral | 0.856 | Effect 91% | Disease | Cannot score substitution |
| rs121918160 | NP_000303 | 461 | S312L | Damaging | Probably Damaging | Neutral | 0.847 | Effect 95% | Disease | Cannot score substitution |
