# Supplementary material for: Investigation of pathogenic germline variants in gastric cancer and development of “GasCanBase” database
Source: Cancer Rep (Hoboken). 2023 Oct 22;6(12):e1906. doi: 10.1002/cnr2.1906 (PMC10728505; doi:10.1002/cnr2.1906)
Supplement: Supplementary file 1 — Data S1 Supporting Information. [file CNR2-6-e1906-s001.zip › Supplementary File/Table S49. Prediction of damaging effect on AURKA.docx]

Table S49. Prediction of damaging effect on AURKA

| **SNP** | **Protein ID** | **Amino acid** | **Amino acid change** | **SIFT** | **PolyPhen2** | **PMut** | **MutPred** | **SNAP2** | **SNP&GO** | **PANTHER** |
| --- | --- | --- | --- | --- | --- | --- | --- | --- | --- | --- |
| rs11539196 | NP_003591 | 403 | G325W | Damaging | Probably Damaging | 0.8377 Pathological | 0.947 | Effect 91% | Disease | Probably Damaging |
| rs2230743 | NP_003591 | 403 | S104L | Damaging | Benign | Neutral | 0.204 | Effect 59% | Neutral | Probably Benign |
| rs33923703 | NP_003591 | 403 | M373V | Damaging | Benign | Neutral | 0.442 | Effect 75% | Neutral | Probably Benign |
| rs34572020 | NP_003591 | 403 | P50L | Damaging | Benign | Neutral | 0.317 | Effect 80% | Neutral | Probably Benign |
| rs45483697 | NP_003591 | 403 | G198S | Damaging | Probably Damaging | Neutral | 0.635 | Effect 53% | Neutral | Probably Damaging |
| rs45520831 | NP_003591 | 403 | R179K | Damaging | Possibly Damaging | Neutral | 0.626 | Effect 59% | Neutral | Probably Damaging |
| rs45557632 | NP_003591 | 403 | F348L | Damaging | Benign | Neutral | 0.650 | Effect 71% | Neutral | Possibly Damaging |
