# Supplementary material for: Investigation of pathogenic germline variants in gastric cancer and development of “GasCanBase” database
Source: Cancer Rep (Hoboken). 2023 Oct 22;6(12):e1906. doi: 10.1002/cnr2.1906 (PMC10728505; doi:10.1002/cnr2.1906)
Supplement: Supplementary file 1 — Data S1 Supporting Information. [file CNR2-6-e1906-s001.zip › Supplementary File/Table S5. Gene networking of Gastric and Colon Cancer genes.docx]

**Table S5. Gene networking of Gastric and Colon Cancer genes**

| **Gene 1** | **Gene 2** | **Weight** | **Network group** |
| --- | --- | --- | --- |
| TP53 | MSH2 | 0.00959636 | Co-expression |
| MSH6 | TP53 | 0.008412687 | Co-expression |
| MSH6 | MSH2 | 0.016528148 | Co-expression |
| MSH6 | KRAS | 0.006211968 | Co-expression |
| E2F3 | KRAS | 0.02353409 | Co-expression |
| PIK3R2 | TP53 | 0.02008692 | Co-expression |
| MSH6 | MSH2 | 0.014397356 | Co-expression |
| NELFB | APC | 0.013481008 | Co-expression |
| SIN3A | APC | 0.008382023 | Co-expression |
| PIK3CA | APC | 0.013220066 | Co-expression |
| MSH6 | MSH2 | 0.017393105 | Co-expression |
| SIN3A | PIK3CA | 0.010976235 | Co-expression |
| MSH6 | MSH2 | 0.010679489 | Co-expression |
| PIK3R1 | PIK3CA | 0.009916827 | Co-expression |
| MSH6 | MSH2 | 0.005778242 | Co-expression |
| MSH2 | APC | 0.006927346 | Co-expression |
| MSH6 | MSH2 | 0.007878492 | Co-expression |
| MSH6 | MSH2 | 0.011610979 | Co-expression |
| APPL1 | PIK3CA | 0.015677959 | Co-expression |
| APPL1 | MSH2 | 0.009007691 | Co-expression |
| RAF1 | KRAS | 0.011892636 | Co-expression |
| MSH3 | MSH2 | 0.008016028 | Co-expression |
| MLH1 | MSH2 | 0.014582656 | Co-localization |
| MLH1 | MSH2 | 0.008900994 | Co-localization |
| E2F3 | MSH2 | 0.004420229 | Co-localization |
| PIK3R2 | APC | 0.006861774 | Co-localization |
| MSH6 | MSH2 | 0.55178285 | Co-localization |
| APPL1 | PIK3CA | 0.7340486 | Co-localization |
| MLH1 | MSH2 | 0.3871396 | Co-localization |
| E2F3 | MSH2 | 0.55178285 | Co-localization |
| MDM2 | TP53 | 0.06781394 | Co-localization |
| PIK3CA | KRAS | 0.3899191 | Genetic Interactions |
| MDM2 | TP53 | 0.08127491 | Genetic Interactions |
