# Supplementary material for: Investigation of pathogenic germline variants in gastric cancer and development of “GasCanBase” database
Source: Cancer Rep (Hoboken). 2023 Oct 22;6(12):e1906. doi: 10.1002/cnr2.1906 (PMC10728505; doi:10.1002/cnr2.1906)
Supplement: Supplementary file 1 — Data S1 Supporting Information. [file CNR2-6-e1906-s001.zip › Supplementary File/Table S5.1. Primer and Restriction enzyme selection of ABCB1 gene.docx]

1. Primer design for selected nsSNP of ABCB1 gene

| Primer Criteria | Forward Primer | Reverse Primer |
| --- | --- | --- |
| Sequence | CCCAGTTCAGACACAAGCAC | TCCAGTTTCCTTTTGGAGGA |
| Length | 20 bp | 20 bp |
| Start | 440 | 655 |
| Tm | 59.3 °C | 59.6 °C |
| GC | 55.0 % | 45.0 % |
| Tm | 55.79 °C | 57.11 °C |
| GC% | 55.0 | 45.0 |
| Self-Dimer ( ΔG) |  | -7.6 kcal/mol |
| Hairpin ( ΔG) |  | -3.4 kcal/mol |
| Cross Dimer (ΔG) |  | |
| Product size | 216 bp | |

2. Restriction enzyme for selected nsSNP of ABCB1 gene

| Enzyme Name | Position | Recognition Site |
| --- | --- | --- |
| MseI | 85 649 732 836 912 1052 1079 1088 | T/TAA |
