# Supplementary material for: Investigation of pathogenic germline variants in gastric cancer and development of “GasCanBase” database
Source: Cancer Rep (Hoboken). 2023 Oct 22;6(12):e1906. doi: 10.1002/cnr2.1906 (PMC10728505; doi:10.1002/cnr2.1906)
Supplement: Supplementary file 1 — Data S1 Supporting Information. [file CNR2-6-e1906-s001.zip › Supplementary File/Table S5.2. Primer and Restriction enzyme selection of APC gene.docx]

1. Primer design for selected nsSNP of APC gene

| Primer Criteria | Forward Primer | Reverse Primer |
| --- | --- | --- |
| Sequence | TCCAAGGTCTTCAATGATAAGC | TCCCTGGAAAGGTCAACATC |
| Length | 22 bp | 20 bp |
| Start | 486 | 658 |
| Tm | 58.3 °C | 59.9 °C |
| GC | 40.9 % | 50.0 % |
| Tm | 56.5 °C | 56.96 °C |
| GC% | 40.91 | 50.0 |
| Self-Dimer ( ΔG) | -4.54 kcal/mol |  |
| Hairpin ( ΔG) | -0.44 kcal/mol | -0.17 kcal/mol |
| Cross Dimer (ΔG) | -6.6 kcal/mol | |
| Product size | 173 bp | |

2. Restriction enzyme for selected nsSNP of APC gene

| Enzyme Name | Position | Recognition Site |
| --- | --- | --- |
| TfiI | 46 487 580 637 724 | G/AWTC |
