# Supplementary material for: Investigation of pathogenic germline variants in gastric cancer and development of “GasCanBase” database
Source: Cancer Rep (Hoboken). 2023 Oct 22;6(12):e1906. doi: 10.1002/cnr2.1906 (PMC10728505; doi:10.1002/cnr2.1906)
Supplement: Supplementary file 1 — Data S1 Supporting Information. [file CNR2-6-e1906-s001.zip › Supplementary File/Table S5.3. Primer and Restriction enzyme selection of CDKN1B gene.docx]

1. Primer design for selected nsSNP of CDKN1B gene

| Primer Criteria | Forward Primer | Reverse Primer |
| --- | --- | --- |
| Sequence | CGCTTTGTTTTGTTCGGTTT | TCTCTGCAGTGCTTCTCCAA |
| Length | 20 bp | 20 bp |
| Start | 405 | 625 |
| Tm | 60.1 °C | 59.9 °C |
| GC | 40.0 % | 50.0 % |
| Tm | 58.26 °C | 56.44 °C |
| GC% | 40.0 | 50.0 |
| Self-Dimer ( ΔG) |  | -10.24  kcal/mol |
| Hairpin ( ΔG) |  |  |
| Cross Dimer (ΔG) | -4.9  kcal/mol | |
| Product size | 221 bp | |

2. Restriction enzyme for selected nsSNP of CDKN1B gene

| Enzyme Name | Position | Recognition Site |
| --- | --- | --- |
| BsrBI | 18 33 175 276 549 | GAGCGG |
