# Supplementary material for: Investigation of pathogenic germline variants in gastric cancer and development of “GasCanBase” database
Source: Cancer Rep (Hoboken). 2023 Oct 22;6(12):e1906. doi: 10.1002/cnr2.1906 (PMC10728505; doi:10.1002/cnr2.1906)
Supplement: Supplementary file 1 — Data S1 Supporting Information. [file CNR2-6-e1906-s001.zip › Supplementary File/Table S5.4. Primer and Restriction enzyme selection of CTNNB1 gene.docx]

1. Primer design for selected nsSNP of CTNNB1 gene

| Primer Criteria | Forward Primer | Reverse Primer |
| --- | --- | --- |
| Sequence | AAGCGGCTGTTAGTCACTGG | AAAATCCCTGTTCCCACTCA |
| Length | 20 bp | 20 bp |
| Start | 507 | 662 |
| Tm | 60.5 °C | 59.4 °C |
| GC | 55.0 % | 45.0 % |
| Tm | 57.91 °C | 56.74 °C |
| GC% | 55.0 | 45.0 |
| Self-Dimer ( ΔG) | -3.94 kcal/mol |  |
| Hairpin ( ΔG) |  |  |
| Cross Dimer (ΔG) | -6.02 kcal/mol | |
| Product size | 156 bp | |

2. Restriction enzyme for selected nsSNP of CTNNB1gene

| Enzyme Name | Position | Recognition Site |
| --- | --- | --- |
| TfiI | 200 255 397 552 | G/AWTC |

| **TfiI** | \| [Help](javascript:void(0)) \| \| --- \| |  | \| [Comments](javascript:void(0)) \| \| --- \| |
| --- | --- | --- | --- | --- | --- |

| [[Back to main display](http://nc2.neb.com/NEBcutter2/cutshow.php?name=53b2da32-)] | \| **5'...** \|  \|  \|  \| **G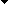A W T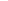C** \|  \|  \|  \| **... 3'** \| \| --- \| --- \| --- \| --- \| --- \| --- \| --- \| --- \| --- \| \| **3'...** \|  \|  \|  \| **C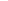T W A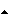G** \|  \|  \|  \| **... 5'** \| |
| --- | --- | --- | --- | --- | --- | --- | --- | --- | --- | --- | --- | --- | --- | --- | --- | --- | --- | --- | --- |

Enzyme No. Positions Recognition
name cuts of sites sequence
TfiI 4 200 255 397 552 g/awtc

| Pair 3: |  |  |  | |  |  |  |
| --- | --- | --- | --- | --- | --- | --- | --- |
|  Left Primer 3:      | | | | | | |  |
| Sequence: |  | | | | | |  |
| Start:   507 | Length:   20 bp | Tm:   60.5 °C | GC:   55.0 % | | ANY:   5.0 | SELF:   1.0 |  |
|  | | | | | | |  |
|  Right Primer 3:      | | | | | | |  |
| Sequence: |  | | | | | |  |
| Start:   662 | Length:   20 bp | Tm:   59.4 °C | GC:   45.0 % | | ANY:   2.0 | SELF:   1.0 |  |
|  | | | | | | |  |
| Product Size:   156 bp | | Pair Any: 3.0 | Pair End: 3.0 | |  |  |  |
| **Analysis Results #1: AAGCGGCTGTTAGTCACTGG** | | | | | | | |
| \| Rating \| : \| 92.0 \|  \| \| --- \| --- \| --- \| --- \| \| Molecular Wt \| : \| 6173.09 \|  \| \| Tm \| : \| 57.91 \| °C \| \| GC% \| : \| 55.0 \|  \| \| GC Clamp \| : \| 2 \|  \| \| nmol/A_260_ \| : \| 5.19 \|  \| \| ug/A_260_ \| : \| 32.03 \|  \| \| ΔG \| : \| -34.23 \| kcal/mol \| | | | | \| 3' end stability \| : \| -7.96 \| kcal/mol \| \| --- \| --- \| --- \| --- \| \| ΔH \| : \| -154.0 \| kcal/mol \| \| ΔS \| : \| -0.4 \| kcal/°K/mol \| \| 5' end ΔG \| : \| -10.29 \| kcal/mol \| \| Self Dimer ( ΔG) \| : \| [-3.94](http://www.premierbiosoft.com/NetPrimer/www.premierbiosoft.com) \| kcal/mol \| \| Hairpin ( ΔG) \| : \|  \| kcal/mol \| \| Repeats (# of pairs) \| : \|  \| kcal/mol \| \| Run (# of bases) \| : \|  \| kcal/mol \| | | | |

| **Analysis Results #2: AAAATCCCTGTTCCCACTCA** | |
| --- | --- |
| \| Rating \| : \| 100.0 \|  \| \| --- \| --- \| --- \| --- \| \| Molecular Wt \| : \| 5981.01 \|  \| \| Tm \| : \| 56.74 \| °C \| \| GC% \| : \| 45.0 \|  \| \| GC Clamp \| : \| 1 \|  \| \| nmol/A_260_ \| : \| 5.38 \|  \| \| ug/A_260_ \| : \| 32.16 \|  \| \| ΔG \| : \| -33.49 \| kcal/mol \| | \| 3' end stability \| : \| -6.47 \| kcal/mol \| \| --- \| --- \| --- \| --- \| \| ΔH \| : \| -151.8 \| kcal/mol \| \| ΔS \| : \| -0.4 \| kcal/°K/mol \| \| 5' end ΔG \| : \| -7.31 \| kcal/mol \| \| Self Dimer ( ΔG) \| : \|  \| kcal/mol \| \| Hairpin ( ΔG) \| : \|  \| kcal/mol \| \| Repeats (# of pairs) \| : \|  \| kcal/mol \| \| Run (# of bases) \| : \| [4](http://www.premierbiosoft.com/NetPrimer/www.premierbiosoft.com) \| kcal/mol \| |

| \| Cross Dimer (ΔG) \| : \| [-6.02](http://www.premierbiosoft.com/NetPrimer/www.premierbiosoft.com) \| kcal/mol \| \| --- \| --- \| --- \| --- \| |
| --- | --- | --- | --- | --- |
