# Supplementary material for: Investigation of pathogenic germline variants in gastric cancer and development of “GasCanBase” database
Source: Cancer Rep (Hoboken). 2023 Oct 22;6(12):e1906. doi: 10.1002/cnr2.1906 (PMC10728505; doi:10.1002/cnr2.1906)
Supplement: Supplementary file 1 — Data S1 Supporting Information. [file CNR2-6-e1906-s001.zip › Supplementary File/Table S51. Prediction of damaging effect on BMPR1A.docx]

Table S51. Prediction of damaging effect on BMPR1A

| **SNP** | **Protein ID** | **Position** | **Amino acid change** | **SIFT** | **PolyPhen2** | **PMut** | **MutPred** | **SNAP2** | **SNP&GO** | **PANTHER** |
| --- | --- | --- | --- | --- | --- | --- | --- | --- | --- | --- |
| rs35619497 | NP_004320 | 532 | R443C | Damaging | Probably Damaging | 0.8527 Pathological | 0.965 | Effect 91% | Disease | Probably Damaging |
| rs112603659 | NP_004320 | 532 | E502G | Damaging | Possibly Damaging | 0.9144 Pathological | 0.742 | Effect 71% | Neutral | Probably Damaging |
| rs112883778 | NP_004320 | 532 | W487R | Damaging | Probably Damaging | 0.9741 Pathological | 0.750 | Effect 85% | Disease | Probably Damaging |
