# Supplementary material for: Investigation of pathogenic germline variants in gastric cancer and development of “GasCanBase” database
Source: Cancer Rep (Hoboken). 2023 Oct 22;6(12):e1906. doi: 10.1002/cnr2.1906 (PMC10728505; doi:10.1002/cnr2.1906)
Supplement: Supplementary file 1 — Data S1 Supporting Information. [file CNR2-6-e1906-s001.zip › Supplementary File/Table S52. Prediction of damaging effect on CASP3.docx]

Table S52. Prediction of damaging effect on CASP3

| **SNP** | **Protein ID** | **Amino acid** | **Amino acid change** | **SIFT** | **PolyPhen2** | **PMut** | **MutPred** | **SNAP2** | **SNP&GO** | **PANTHER** |
| --- | --- | --- | --- | --- | --- | --- | --- | --- | --- | --- |
| rs1049210 | NP_004337 | 277 | E190D | Damaging | Probably Damaging | Neutral | 0.215 | Effect 66% | Neutral | Possibly Damaging |
| rs79348369 | NP_004337 | 277 | S249Y | Damaging | Probably Damaging | 0.8058 Pathological | 0.502 | Effect 63% | Disease | Possibly Damaging |
| rs111512673 | NP_004337 | 277 | L91P | Damaging | Probably Damaging | Neutral | 0.767 | Neutral | Disease | Possibly Damaging |
| rs35578277 | NP_004337 | 277 | H22R | Damaging | Possibly Damaging | Pathological | 0.093 | Effect 71% | Neutral | Possibly Damaging |
| rs78678473 | NP_004337 | 277 | V117A | Damaging | Probably Damaging | Neutral | 0.681 | Neutral | Neutral | Probably Damaging |
| rs80000647 | NP_004337 | 277 | A183V | Damaging | Benign | Neutral | 0.440 | Neutral | Neutral | Possibly Damaging |
