# Supplementary material for: Investigation of pathogenic germline variants in gastric cancer and development of “GasCanBase” database
Source: Cancer Rep (Hoboken). 2023 Oct 22;6(12):e1906. doi: 10.1002/cnr2.1906 (PMC10728505; doi:10.1002/cnr2.1906)
Supplement: Supplementary file 1 — Data S1 Supporting Information. [file CNR2-6-e1906-s001.zip › Supplementary File/Table S53. Prediction of damaging effect on CD44.docx]

Table S53. Prediction of damaging effect on CD44

| **SNP** | **Protein ID** | **Amino acid** | **Amino acid change** | **SIFT** | **PolyPhen2** | **PMut** | **MutPred** | **SNAP2** | **SNP&GO** | **PANTHER** |
| --- | --- | --- | --- | --- | --- | --- | --- | --- | --- | --- |
| rs121909545 | NP_000601 | 742 | R46G | Damaging | Possibly Damaging | 0.7977 Pathological | 0.701 | Effect 91% | Neutral | Possibly Damaging |
| rs1058200 | NP_000601 | 742 | S109Y | Damaging | Possibly Damaging | 0.4898 Neutral | 0.266 | Effect 75% | Neutral | Probably Benign |
| rs61752930 | NP_000601 | 742 | T163M | Damaging | Probably Damaging | 0.8425 Pathological | 0.425 | Neutral | Neutral | Possibly Damaging |
| rs61752932 | NP_000601 | 742 | R673Q | Damaging | Probably Damaging | 0.5768 Pathological | 0.423 | Effect 80% | Disease | Probably Damaging |
| rs61755294 | NP_000601 | 742 | A655P | Damaging | Probably Damaging | 0.9520 Pathological | 0.527 | Effect 80% | Neutral | Probably Benign |
| rs12273397 | NP_000601 | 742 | D494H | Damaging | Probably Damaging | 0.6126 Pathological | 0.233 | Effect 59% | Neutral | Possibly Damaging |
| rs79985395 | NP_000601 | 742 | M461T | Damaging | Benign | 0.8427 Pathological | 0.160 | Effect 53% | Disease | Possibly Damaging |
| rs111509467 | NP_000601 | 742 | I96V | Damaging | Benign | 0.0279 Neutral | 0.374 | Effect 59% | Neutral | Probably Benign |
