# Supplementary material for: Investigation of pathogenic germline variants in gastric cancer and development of “GasCanBase” database
Source: Cancer Rep (Hoboken). 2023 Oct 22;6(12):e1906. doi: 10.1002/cnr2.1906 (PMC10728505; doi:10.1002/cnr2.1906)
Supplement: Supplementary file 1 — Data S1 Supporting Information. [file CNR2-6-e1906-s001.zip › Supplementary File/Table S54. Prediction of damaging effect on CDH1.docx]

Table S54. Prediction of damaging effect on CDH1

| **SNP** | **Protein ID** | **Amino acid** | **Amino acid change** | **SIFT** | **PolyPhen2** | **PMut** | **MutPred** | **SNAP2** | **SNP&GO** | **PANTHER** |
| --- | --- | --- | --- | --- | --- | --- | --- | --- | --- | --- |
| rs34466743 | NP_004351 | 882 | I393N | Damaging | Probably Damaging | 0.5681  Pathological | 0.286 | Effect 85% | Disease | Possibly Damaging |
| rs34507583 | NP_004351 | 882 | E880K | Damaging | Possibly Damaging | 0.5372  Pathological | 0.131 | Effect 85% | Disease | Probably Damaging |
| rs9282655 | NP_004351 | 882 | C695R | Damaging | Probably Damaging | 0.8448  Pathological | 0.345 | Effect 75% | Disease | Probably Damaging |
| rs2276331 | NP_004351 | 882 | L630V | Damaging | Probably Damaging | Neutral | 0.316 | Effect 85% | Neutral | Probably Damaging |
| rs35187787 | NP_004351 | 882 | A592S | Damaging | Benign | Neutral | 0.647 | Neutral | Neutral | Probably Benign |
| rs35187787 | NP_004351 | 882 | A592T | Damaging | Possibly Damaging | Neutral | 0.843 | Effect 85% | Neutral | Probably Benign |
| rs35572355 | NP_004351 | 882 | V832M | Damaging | Probably Damaging | Neutral | 0.861 | Effect 91% | Disease | Probably Damaging |
| rs115934514 | NP_004351 | 882 | V574F | Damaging | Benign | 0.6001 Pathological | 0.371 | Neutral | Neutral | Probably Benign |
| rs121964871 | NP_004351 | 882 | L711V | Damaging | Possibly Damaging | Neutral | 0.413 | Effect 80% | Neutral | Possibly Damaging |
| rs121964878 | NP_004351 | 882 | A634V | Damaging | Benign | Neutral | 0.365 | Neutral | Neutral | Possibly Damaging |
| rs56836234 | NP_004351 | 882 | D498E | Damaging | Possibly Damaging | Neutral | 0.536 | Neutral | Neutral | Probably Damaging |
