# Supplementary material for: Investigation of pathogenic germline variants in gastric cancer and development of “GasCanBase” database
Source: Cancer Rep (Hoboken). 2023 Oct 22;6(12):e1906. doi: 10.1002/cnr2.1906 (PMC10728505; doi:10.1002/cnr2.1906)
Supplement: Supplementary file 1 — Data S1 Supporting Information. [file CNR2-6-e1906-s001.zip › Supplementary File/Table S55. Prediction of damaging effect on CDK1B.docx]

Table S55. Prediction of damaging effect on CDK1B

| **SNP** | **Protein ID** | **Amino Acid** | **Amino acid change** | **SIFT** | **PolyPhen2** | **PMut** | **MutPred** | **SNAP2** | **SNP&GO** | **PANTHER** |
| --- | --- | --- | --- | --- | --- | --- | --- | --- | --- | --- |
| rs2066828 | NP_004055 | 198 | R15W | Damaging | Probably Damaging | 0.9874 Pathological | 0.190 | Effect 85% | Neutral | Probably Damaging |
