# Supplementary material for: Investigation of pathogenic germline variants in gastric cancer and development of “GasCanBase” database
Source: Cancer Rep (Hoboken). 2023 Oct 22;6(12):e1906. doi: 10.1002/cnr2.1906 (PMC10728505; doi:10.1002/cnr2.1906)
Supplement: Supplementary file 1 — Data S1 Supporting Information. [file CNR2-6-e1906-s001.zip › Supplementary File/Table S56. Prediction of damaging effect on CEACAM5.docx]

Table S56. Prediction of damaging effect on CEACAM5

| **SNP** | **Protein ID** | **Amino acid** | **Amino acid change** | **SIFT** | **PolyPhen2** | **PMut** | **MutPred** | **SNAP2** | **SNP&GO** | **PANTHER** |
| --- | --- | --- | --- | --- | --- | --- | --- | --- | --- | --- |
| rs3815780 | NP_004354 | 702 | Q137P | Damaging | Probably Damaging | 0.6186 Pathological | 0.553 | Effect 95% | Disease | Probably Benign |
| rs10407503 | NP_004354 | 702 | A340D | Damaging | Benign | Neutral | 0.394 | Effect 80% | Neutral | Cannot Score Substitution |
| rs17853315 | NP_004354 | 702 | T210S | Damaging | Benign | Neutral | 0.473 | Neutral | Neutral | Cannot Score Substitution |
| rs61735258 | NP_004354 | 702 | Q282R | Damaging | Possibly Damaging | Neutral | 0.712 | Effect 66% | Neutral | Cannot Score Substitution |
| rs75927810 | NP_004354 | 702 | V335M | Damaging | Benign | Neutral | 0.708 | Neutral | Neutral | Cannot Score Substitution |
| rs112998286 | NP_004354 | 702 | I451N | Damaging | Probably Damaging | 0.6144 Pathological | 0.503 | Effect 53% | Neutral | Cannot Score Substitution |
| rs113097830 | NP_004354 | 702 | P186Q | Damaging | Probably Damaging | 0.6782 Pathological | 0.378 | Neutral | Neutral | Cannot Score Substitution |
