# Supplementary material for: Investigation of pathogenic germline variants in gastric cancer and development of “GasCanBase” database
Source: Cancer Rep (Hoboken). 2023 Oct 22;6(12):e1906. doi: 10.1002/cnr2.1906 (PMC10728505; doi:10.1002/cnr2.1906)
Supplement: Supplementary file 1 — Data S1 Supporting Information. [file CNR2-6-e1906-s001.zip › Supplementary File/Table S57. Prediction of damaging effect on CTNNA1.docx]

Table S57. Prediction of damaging effect on CTNNA1

| **SNP** | **Protein ID** | **Amino acid** | **Amino acid change** | **SIFT** | **PolyPhen2** | **PMut** | **MutPred** | **SNAP2** | **SNP&GO** | **PANTHER** |
| --- | --- | --- | --- | --- | --- | --- | --- | --- | --- | --- |
| rs28363394 | NP_001894 | 906 | A179V | Damaging | Benign | 0.6646 Pathological | 0.181 | Effect 66% | Neutral | Probably Damaging |
| rs28363406 | NP_001894 | 906 | P219S | Damaging | Benign | Neutral | 0.512 | Effect 71% | Neutral | Probably Damaging |
| rs35788568 | NP_001894 | 906 | M124I | Damaging | Possibly Damaging | Neutral | 0.617 | Neutral | Neutral | Probably Damaging |
| rs35879875 | NP_001894 | 906 | R302K | Damaging | Possibly Damaging | Neutral | 0.581 | Effect 53% | Neutral | Probably Damaging |
| rs74648499 | NP_001894 | 906 | R194K | Damaging | Probably Damaging | Neutral | 0.790 | Effect 75% | Neutral | Probably Damaging |
