# Supplementary material for: Investigation of pathogenic germline variants in gastric cancer and development of “GasCanBase” database
Source: Cancer Rep (Hoboken). 2023 Oct 22;6(12):e1906. doi: 10.1002/cnr2.1906 (PMC10728505; doi:10.1002/cnr2.1906)
Supplement: Supplementary file 1 — Data S1 Supporting Information. [file CNR2-6-e1906-s001.zip › Supplementary File/Table S58. Prediction of damaging effect on CTNNB1.docx]

Table S58. Prediction of damaging effect on CTNNB1

| **SNP** | **Protein ID** | **Amino acid** | **Amino acid change** | **SIFT** | **PolyPhen2** | **PMut** | **MutPred** | **SNAP2** | **SNP&GO** | **PANTHER** |
| --- | --- | --- | --- | --- | --- | --- | --- | --- | --- | --- |
| rs28931588 | NP_001091679 | 781 | D32N | Damaging | Probably Damaging | Neutral | 0.397 | Effect 95% | Neutral | Cannot Score Substitution |
| rs28931589 | NP_001091679 | 781 | G34E | Damaging | Probably Damaging | 0.5552 Pathological | 0.466 | Effect 95% | Neutral | Cannot Score Substitution |
| rs35288908 | NP_001091679 | 781 | N287S | Damaging | Benign | Neutral | 0.308 | Neutral | Neutral | Cannot Score Substitution |
| rs121913396 | NP_001091679 | 781 | D32A | Damaging | Probably Damaging | 0.6036 Pathological | 0.395 | Effect 91% | Neutral | Cannot Score Substitution |
| rs121913400 | NP_001091679 | 781 | S33C | Damaging | Probably Damaging | Neutral | 0.353 | Effect 95% | Neutral | Cannot Score Substitution |
| rs121913403 | NP_001091679 | 781 | S37C | Damaging | Probably Damaging | Neutral | 0.428 | Effect 95% | Neutral | Cannot Score Substitution |
| rs121913407 | NP_001091679 | 781 | S45A | Damaging | Possibly Damaging | Neutral | 0.200 | Effect 80% | Neutral | Cannot Score Substitution |
| rs121913409 | NP_001091679 | 781 | S45F | Damaging | Probably Damaging | 0.8075 Pathological | 0.304 | Effect 85% | Neutral | Cannot Score Substitution |
| rs121913412 | NP_001091679 | 781 | T41A | Damaging | Possibly Damaging | Neutral | 0.283 | Effect 85% | Neutral | Cannot Score Substitution |
| rs121913413 | NP_001091679 | 781 | T41I | Damaging | Probably Damaging | Neutral | 0.352 | Effect 91% | Neutral | Cannot Score Substitution |
| rs4135384 | NP_001091679 | 781 | M688V | Damaging | Benign | 0.5404 Pathological | 0.236 | Neutral | Neutral | Cannot Score Substitution |
| rs77064436 | NP_001091679 | 781 | V22A | Damaging | Benign | Neutral | 0.522 | Effect 59% | Neutral | Cannot Score Substitution |
| rs77624106 | NP_001091679 | 781 | S179Y | Damaging | Probably Damaging | 0.7700 Pathological | 0.487 | Effect 75% | Neutral | Cannot Score Substitution |
| rs77750814 | NP_001091679 | 781 | D665E | Damaging | Benign | Neutral | 0.286 | Neutral | Neutral | Cannot Score Substitution |
| rs113411271 | NP_001091679 | 781 | R486C | Damaging | Probably Damaging | 0.8076 Pathological | 0.652 | Neutral | Neutral | Cannot Score Substitution |
| rs121913228 | NP_001091679 | 781 | S37A | Damaging | Probably Damaging | Neutral | 0.404 | Effect 91% | Neutral | Cannot Score Substitution |
| rs121913394 | NP_001091679 | 781 | A13T | Damaging | Benign | Neutral | 0.210 | Neutral | Neutral | Cannot Score Substitution |
| rs121913395 | NP_001091679 | 781 | A21T | Damaging | Benign | Neutral | 0.254 | Neutral | Neutral | Cannot Score Substitution |
| rs121913399 | NP_001091679 | 781 | G34R | Damaging | Benign | 0.5791 Pathological | 0.487 | Effect 95% | Neutral | Cannot Score Substitution |
