# Supplementary material for: Investigation of pathogenic germline variants in gastric cancer and development of “GasCanBase” database
Source: Cancer Rep (Hoboken). 2023 Oct 22;6(12):e1906. doi: 10.1002/cnr2.1906 (PMC10728505; doi:10.1002/cnr2.1906)
Supplement: Supplementary file 1 — Data S1 Supporting Information. [file CNR2-6-e1906-s001.zip › Supplementary File/Table S59. Prediction of damaging effect on DCC.docx]

Table S59. Prediction of damaging effect on DCC

| **SNP** | **Protein ID** | **Amino acid** | **Amino acid change** | **SIFT** | **PolyPhen2** | **PMut** | **MutPred** | **SNAP2** | **SNP&GO** | **PANTHER** |
| --- | --- | --- | --- | --- | --- | --- | --- | --- | --- | --- |
| rs35691189 | NP_005206 | 1447 | N702S | Damaging | Probably Damaging | Neutral | 0.484 | Effect 66% | Disease | Probably Damaging |
| rs2270950 | NP_005206 | 1447 | H1191L | Damaging | Possibly Damaging | 0.9643 Pathological | 0.276 | Effect 66% | Neutral | Probably Damaging |
| rs2271042 | NP_005206 | 1447 | L679R | Damaging | Benign | Neutral | 0.346 | Effect 66% | Neutral | Probably Damaging |
| rs2278339 | NP_005206 | 1447 | I759M | Damaging | Possibly Damaging | Neutral | 0.304 | Effect 71% | Neutral | Probably Damaging |
| rs34573287 | NP_005206 | 1447 | K640T | Damaging | Benign | Neutral | 0.601 | Effect 63% | Neutral | Probably Damaging |
| rs112302456 | NP_005206 | 1447 | V621M | Damaging | Probably Damaging | Neutral | 0.503 | Neutral | Neutral | Probably Damaging |
| rs113366086 | NP_005206 | 1447 | S569T | Damaging | Benign | Neutral | 0.471 | Neutral | Neutral | Probably Damaging |
| rs116498325 | NP_005206 | 1447 | Q486L | Damaging | Probably Damaging | 0.6746 Pathological | 0.480 | Effect 71% | Neutral | Probably Damaging |
| rs117282798 | NP_005206 | 1447 | N3S | Damaging | Benign | Neutral | 0.353 | Neutral | Neutral | Possibly Damaging |
