# Supplementary material for: Investigation of pathogenic germline variants in gastric cancer and development of “GasCanBase” database
Source: Cancer Rep (Hoboken). 2023 Oct 22;6(12):e1906. doi: 10.1002/cnr2.1906 (PMC10728505; doi:10.1002/cnr2.1906)
Supplement: Supplementary file 1 — Data S1 Supporting Information. [file CNR2-6-e1906-s001.zip › Supplementary File/Table S6. Gene networking of Gastric and Lung Cancer genes.docx]

**Table S6. Gene networking of Gastric and Lung Cancer genes**

| Gene 1 | Gene 2 | Weight | Network group |
| --- | --- | --- | --- |
| SHOC2 | KRAS | 0.009137684 | Co-expression |
| RHOA | MET | 0.021096658 | Co-expression |
| RAF1 | KRAS | 0.011892636 | Co-expression |
| RHOA | MET | 0.013627649 | Co-expression |
| CBL | MET | 0.7056816 | Co-localization |
| RAF1 | KRAS | 0.3899191 | Genetic Interactions |
| SHC1 | KRAS | 0.3899191 | Genetic Interactions |
| CBL | MET | 0.00446139 | Pathway |
| HGF | MET | 0.012195868 | Pathway |
| RAF1 | KRAS | 0.008205559 | Pathway |
| PAX3 | MET | 0.066735476 | Pathway |
| RHOA | KRAS | 0.003456038 | Pathway |
| INPPL1 | MET | 0.024139082 | Pathway |
| RGL2 | KRAS | 0.07931504 | Pathway |
| SHC1 | KRAS | 0.004475513 | Pathway |
| SHC1 | MET | 0.003929855 | Pathway |
| SHOC2 | KRAS | 0.1106227 | Pathway |
| MUC20 | MET | 0.07181805 | Pathway |
| ARHGAP35 | MET | 0.30825618 | Pathway |
| RHOA | MET | 0.2228451 | Pathway |
| RRAS | MET | 0.20801303 | Pathway |
| HGF | MET | 0.017483111 | Pathway |
| PAX3 | MET | 0.320779 | Pathway |
| INPPL1 | MET | 0.19073634 | Pathway |
