# Supplementary material for: Investigation of pathogenic germline variants in gastric cancer and development of “GasCanBase” database
Source: Cancer Rep (Hoboken). 2023 Oct 22;6(12):e1906. doi: 10.1002/cnr2.1906 (PMC10728505; doi:10.1002/cnr2.1906)
Supplement: Supplementary file 1 — Data S1 Supporting Information. [file CNR2-6-e1906-s001.zip › Supplementary File/Table S6.1. Allele specific primer design on selected nsSNP of ABCB1 gene.docx]

[rs41316450](https://www.ncbi.nlm.nih.gov/projects/SNP/snp_ref.cgi?rs=41316450) *[Homo sapiens]*

ACAAATGGGCATCACACTTACCCCT[A/T]TAATCTTTGAAAATATTATTGCAAA

Chromosome: 7:87544133

Gene: ABCB1

1. Allele specific primer design on wild type nucleotide of ABCB1 gene

|  | Forward Primer | Reverse Primer |
| --- | --- | --- |
| Sequence | GGGCATCACACTTACCCCTA | CCTCCAGTTTCCTTTTGGAG |
| Length | 20 bp | 20 bp |
| Start | 508 | 657 |
| Tm | 59.8 °C | 58.8 °C |
| GC | 55.0 % | 50.0 % |
| Tm | 57.3 °C | 56.44 °C |
| GC% | 55.0 | 50.0 |
| Self-Dimer ( ΔG) | -7.14 kcal/mol | -9.2 kcal/mol |
| Hairpin ( ΔG) | -2.64 kcal/mol | -5.0 kcal/mol |
| Cross Dimer (ΔG) |  | |
| Product size | 150 bp | |

2. Allele specific primer design on mutant nucleotide of ABCB1 gene

|  | Forward Primer | Reverse Primer |
| --- | --- | --- |
| Sequence | GGGCATCACACTTACCCCTT | ACCTCCAGTTTCCTTTTGGA |
| Length | 20 bp | 20 bp |
| Start | 508 | 658 |
| Tm | 61.1 °C | 58.6 °C |
| GC | 55.0 % | 45.0 % |
| Tm | 58.76 °C | 56.22 °C |
| GC% | 55.0 | 45.0 |
| Self-Dimer ( ΔG) | -7.14 kcal/mol | -7.6 kcal/mol |
| Hairpin ( ΔG) | -2.64 kcal/mol | -3.4 kcal/mol |
| Cross Dimer (ΔG) | kcal/mol | |
| Product size | 151 bp | |
