# Supplementary material for: Investigation of pathogenic germline variants in gastric cancer and development of “GasCanBase” database
Source: Cancer Rep (Hoboken). 2023 Oct 22;6(12):e1906. doi: 10.1002/cnr2.1906 (PMC10728505; doi:10.1002/cnr2.1906)
Supplement: Supplementary file 1 — Data S1 Supporting Information. [file CNR2-6-e1906-s001.zip › Supplementary File/Table S6.10. Allele specific primer design on selected nsSNP of CTNNA1 gene.docx]

[rs74648499](https://www.ncbi.nlm.nih.gov/projects/SNP/snp_ref.cgi?rs=74648499) *[Homo sapiens]*

AAGCTGAACATTATGGCAGCCAAAA[A/G]ACAACAGGTACAGTCATGATTTGGG

Chromosome: 5:138812295

Gene:CTNNA1

1. Allele specific primer design on wild type nucleotide of CTNNA1 gene

|  | Forward Primer | Reverse Primer |
| --- | --- | --- |
| Sequence | AACATTATGGCAGCCAAAAG | CAGCACAATACTGAAAAGACACC |
| Length | 20 bp | 23bp |
| Start | 529 | 711 |
| Tm | 57.8 °C | 58.8 °C |
| GC | 40.0 % | 43.5 % |
| Tm | 55.71 °C | 56.99 °C |
| GC% | 40.0 | 43.48 |
| Self-Dimer ( ΔG) | -8.16 kcal/mol |  |
| Hairpin ( ΔG) |  |  |
| Cross Dimer (ΔG) |  | |
| Product size | 183 bp | |

2. Allele specific primer design on mutant nucleotide of CTNNA1 gene

|  | Forward Primer | Reverse Primer |
| --- | --- | --- |
| Sequence | AACATTATGGCAGCCAAAAA | CAGCACAATACTGAAAAGACACC |
| Length | 20 bp | 23 bp |
| Start | 529 | 711 |
| Tm | 58.2 °C | 58.8 °C |
| GC | 35.0 % | 43.5 % |
| Tm | 56.15 °C | 56.99 °C |
| GC% | 35.0 | 43.48 |
| Self-Dimer ( ΔG) | -8.16  kcal/mol |  |
| Hairpin ( ΔG) |  |  |
| Cross Dimer (ΔG) |  | |
| Product size | 183 bp | |

| Pair 5: |  |  |  |  |  |
| --- | --- | --- | --- | --- | --- |
|  Left Primer 5:      | | | | | |
| Sequence: |  | | | | |
| Start:   529 | Length:   20 bp | Tm:   57.8 °C | GC:   40.0 % | ANY:   9.0 | SELF:   7.0 |
|  | | | | | |
|  Right Primer 5:      | | | | | |
| Sequence: |  | | | | |
| Start:   711 | Length:   23 bp | Tm:   58.8 °C | GC:   43.5 % | ANY:   4.0 | SELF:   0.0 |
|  | | | | | |
| Product Size:   183 bp | | Pair Any: 4.0 | Pair End: 1.0 |  |  |

| **Analysis Results #1: AACATTATGGCAGCCAAAAG** | |
| --- | --- |
| \| Rating \| : \| 85.0 \|  \| \| --- \| --- \| --- \| --- \| \| Molecular Wt \| : \| 6143.11 \|  \| \| Tm \| : \| 55.71 \| °C \| \| GC% \| : \| 40.0 \|  \| \| GC Clamp \| : \| 1 \|  \| \| nmol/A_260_ \| : \| 4.83 \|  \| \| ug/A_260_ \| : \| 29.65 \|  \| \| ΔG \| : \| -33.9 \| kcal/mol \| | \| 3' end stability \| : \| -7.43 \| kcal/mol \| \| --- \| --- \| --- \| --- \| \| ΔH \| : \| -158.2 \| kcal/mol \| \| ΔS \| : \| -0.42 \| kcal/°K/mol \| \| 5' end ΔG \| : \| -6.71 \| kcal/mol \| \| Self Dimer ( ΔG) \| : \| [-8.16](http://www.premierbiosoft.com/NetPrimer/www.premierbiosoft.com) \| kcal/mol \| \| Hairpin ( ΔG) \| : \|  \| kcal/mol \| \| Repeats (# of pairs) \| : \|  \| kcal/mol \| \| Run (# of bases) \| : \| [4](http://www.premierbiosoft.com/NetPrimer/www.premierbiosoft.com) \| kcal/mol \| |

| **Analysis Results #2: CAGCACAATACTGAAAAGACACC** | |
| --- | --- |
| \| Rating \| : \| 100.0 \|  \| \| --- \| --- \| --- \| --- \| \| Molecular Wt \| : \| 7003.69 \|  \| \| Tm \| : \| 56.99 \| °C \| \| GC% \| : \| 43.48 \|  \| \| GC Clamp \| : \| 2 \|  \| \| nmol/A_260_ \| : \| 4.28 \|  \| \| ug/A_260_ \| : \| 29.94 \|  \| \| ΔG \| : \| -35.0 \| kcal/mol \| | \| 3' end stability \| : \| -7.71 \| kcal/mol \| \| --- \| --- \| --- \| --- \| \| ΔH \| : \| -162.7 \| kcal/mol \| \| ΔS \| : \| -0.43 \| kcal/°K/mol \| \| 5' end ΔG \| : \| -8.65 \| kcal/mol \| \| Self Dimer ( ΔG) \| : \|  \| kcal/mol \| \| Hairpin ( ΔG) \| : \|  \| kcal/mol \| \| Repeats (# of pairs) \| : \|  \| kcal/mol \| \| Run (# of bases) \| : \| [4](http://www.premierbiosoft.com/NetPrimer/www.premierbiosoft.com) \| kcal/mol \| |

| \| Cross Dimer (ΔG) \| : \|  \| kcal/mol \| \| --- \| --- \| --- \| --- \| |
| --- | --- | --- | --- | --- |

|  |
| --- |

| \| \|  \| \| --- \| \|  \| \| \| --- \| --- \| --- \| |
| --- | --- | --- | --- |
|  |

000000000000000000000000000000000000000000000000000000000000000000000000

| Pair 5: |  |  |  |  |  |
| --- | --- | --- | --- | --- | --- |
|  Left Primer 5:      | | | | | |
| Sequence: |  | | | | |
| Start:   529 | Length:   20 bp | Tm:   58.2 °C | GC:   35.0 % | ANY:   9.0 | SELF:   7.0 |
|  | | | | | |
|  Right Primer 5:      | | | | | |
| Sequence: |  | | | | |
| Start:   711 | Length:   23 bp | Tm:   58.8 °C | GC:   43.5 % | ANY:   4.0 | SELF:   0.0 |
|  | | | | | |
| Product Size:   183 bp | | Pair Any: 4.0 | Pair End: 0.0 |  |  |

| **Analysis Results #1: AACATTATGGCAGCCAAAAA** | |
| --- | --- |
| \| Rating \| : \| 85.0 \|  \| \| --- \| --- \| --- \| --- \| \| Molecular Wt \| : \| 6127.11 \|  \| \| Tm \| : \| 56.15 \| °C \| \| GC% \| : \| 35.0 \|  \| \| GC Clamp \| : \| 0 \|  \| \| nmol/A_260_ \| : \| 4.77 \|  \| \| ug/A_260_ \| : \| 29.23 \|  \| \| ΔG \| : \| -34.25 \| kcal/mol \| | \| 3' end stability \| : \| -7.78 \| kcal/mol \| \| --- \| --- \| --- \| --- \| \| ΔH \| : \| -159.5 \| kcal/mol \| \| ΔS \| : \| -0.42 \| kcal/°K/mol \| \| 5' end ΔG \| : \| -6.71 \| kcal/mol \| \| Self Dimer ( ΔG) \| : \| [-8.16](http://www.premierbiosoft.com/NetPrimer/www.premierbiosoft.com) \| kcal/mol \| \| Hairpin ( ΔG) \| : \|  \| kcal/mol \| \| Repeats (# of pairs) \| : \|  \| kcal/mol \| \| Run (# of bases) \| : \| [5](http://www.premierbiosoft.com/NetPrimer/www.premierbiosoft.com) \| kcal/mol \| |

| **Analysis Results #2: CAGCACAATACTGAAAAGACACC** | |
| --- | --- |
| \| Rating \| : \| 100.0 \|  \| \| --- \| --- \| --- \| --- \| \| Molecular Wt \| : \| 7003.69 \|  \| \| Tm \| : \| 56.99 \| °C \| \| GC% \| : \| 43.48 \|  \| \| GC Clamp \| : \| 2 \|  \| \| nmol/A_260_ \| : \| 4.28 \|  \| \| ug/A_260_ \| : \| 29.94 \|  \| \| ΔG \| : \| -35.0 \| kcal/mol \| | \| 3' end stability \| : \| -7.71 \| kcal/mol \| \| --- \| --- \| --- \| --- \| \| ΔH \| : \| -162.7 \| kcal/mol \| \| ΔS \| : \| -0.43 \| kcal/°K/mol \| \| 5' end ΔG \| : \| -8.65 \| kcal/mol \| \| Self Dimer ( ΔG) \| : \|  \| kcal/mol \| \| Hairpin ( ΔG) \| : \|  \| kcal/mol \| \| Repeats (# of pairs) \| : \|  \| kcal/mol \| \| Run (# of bases) \| : \| [4](http://www.premierbiosoft.com/NetPrimer/www.premierbiosoft.com) \| kcal/mol \| |

| \| Cross Dimer (ΔG) \| : \|  \| kcal/mol \| \| --- \| --- \| --- \| --- \| |
| --- | --- | --- | --- | --- |

|  |
| --- |

|  |
| --- |
|  |
