# Supplementary material for: Investigation of pathogenic germline variants in gastric cancer and development of “GasCanBase” database
Source: Cancer Rep (Hoboken). 2023 Oct 22;6(12):e1906. doi: 10.1002/cnr2.1906 (PMC10728505; doi:10.1002/cnr2.1906)
Supplement: Supplementary file 1 — Data S1 Supporting Information. [file CNR2-6-e1906-s001.zip › Supplementary File/Table S6.11. Allele specific primer design on selected nsSNP of CTNNB1 gene.docx]

[rs28931589](https://www.ncbi.nlm.nih.gov/projects/SNP/snp_ref.cgi?rs=28931589) *[Homo sapiens]*

CAGCAACAGTCTTACCTGGACTCTG[A/G/T]AATCCATTCTGGTGCCACTACCACA

Chromosome: 3:41224613

Gene: CTNNB1

1. Allele specific primer design on wild type nucleotide of CTNNB1 gene

| Primer Criteria | Forward Primer | Reverse Primer |
| --- | --- | --- |
| Sequence | CAGTCTTACCTGGACTCTGG | TCAAAACTGCATTCTGACTTTCA |
| Length | 20 bp | 23 bp |
| Start | 533 | 743 |
| Tm | 54.8 °C | 59.9 °C |
| GC | 55.0 % | 34.8 % |
| Tm | 51.63 °C | 57.97 °C |
| GC% | 55.0 | 34.78 |
| Self-Dimer ( ΔG) | -4.55 kcal/mol | -7.05 kcal/mol |
| Hairpin ( ΔG) | -0.42 kcal/mol |  |
| Cross Dimer (ΔG) | -4.89 kcal/mol | |
| Product size | 211 bp | |

1. Allele specific primer design on mutant nucleotide of CTNNB1 gene

| Primer Criteria | Forward Primer | Reverse Primer |
| --- | --- | --- |
| Sequence | CAGTCTTACCTGGACTCTGA | TGTTCTTGAGTGAAGGACTGAGA |
| Length | 20 bp | 23 bp |
| Start | 533 | 684 |
| Tm | 52.7 °C | 59.1 °C |
| GC | 50.0 % | 43.5 % |
| Tm | 49.2 °C | 56.64 °C |
| GC% | 50.0 | 43.48 |
| Self-Dimer ( ΔG) | -4.55 kcal/mol | -4.17  kcal/mol |
| Hairpin ( ΔG) | -0.42 kcal/mol |  |
| Cross Dimer (ΔG) | -7.47 kcal/mol | |
| Product size | 152 bp | |

| Pair 1: |  |  |  |  |  |
| --- | --- | --- | --- | --- | --- |
|  Left Primer 1:      | | | | | |
| Sequence: |  | | | | |
| Start:   533 | Length:   20 bp | Tm:   54.8 °C | GC:   55.0 % | ANY:   4.0 | SELF:   1.0 |
|  | | | | | |
|  Right Primer 1:      | | | | | |
| Sequence: |  | | | | |
| Start:   743 | Length:   23 bp | Tm:   59.9 °C | GC:   34.8 % | ANY:   8.0 | SELF:   3.0 |
|  | | | | | |
| Product Size:   211 bp | | Pair Any: 5.0 | Pair End: 1.0 |  |  |

| **Analysis Results #1: CAGTCTTACCTGGACTCTGG** | |
| --- | --- |
| \| Rating \| : \| 91.0 \|  \| \| --- \| --- \| --- \| --- \| \| Molecular Wt \| : \| 6084.04 \|  \| \| Tm \| : \| 51.63 \| °C \| \| GC% \| : \| 55.0 \|  \| \| GC Clamp \| : \| 2 \|  \| \| nmol/A_260_ \| : \| 5.51 \|  \| \| ug/A_260_ \| : \| 33.5 \|  \| \| ΔG \| : \| -30.22 \| kcal/mol \| | \| 3' end stability \| : \| -8.2 \| kcal/mol \| \| --- \| --- \| --- \| --- \| \| ΔH \| : \| -140.8 \| kcal/mol \| \| ΔS \| : \| -0.37 \| kcal/°K/mol \| \| 5' end ΔG \| : \| -6.47 \| kcal/mol \| \| Self Dimer ( ΔG) \| : \| [-4.55](http://www.premierbiosoft.com/NetPrimer/www.premierbiosoft.com) \| kcal/mol \| \| Hairpin ( ΔG) \| : \| [-0.42](http://www.premierbiosoft.com/NetPrimer/www.premierbiosoft.com) \| kcal/mol \| \| Repeats (# of pairs) \| : \|  \| kcal/mol \| \| Run (# of bases) \| : \|  \| kcal/mol \| |

| **Analysis Results #2: TCAAAACTGCATTCTGACTTTCA** | |
| --- | --- |
| \| Rating \| : \| 87.0 \|  \| \| --- \| --- \| --- \| --- \| \| Molecular Wt \| : \| 6957.65 \|  \| \| Tm \| : \| 57.97 \| °C \| \| GC% \| : \| 34.78 \|  \| \| GC Clamp \| : \| 1 \|  \| \| nmol/A_260_ \| : \| 4.64 \|  \| \| ug/A_260_ \| : \| 32.26 \|  \| \| ΔG \| : \| -35.33 \| kcal/mol \| | \| 3' end stability \| : \| -7.42 \| kcal/mol \| \| --- \| --- \| --- \| --- \| \| ΔH \| : \| -162.1 \| kcal/mol \| \| ΔS \| : \| -0.43 \| kcal/°K/mol \| \| 5' end ΔG \| : \| -7.42 \| kcal/mol \| \| Self Dimer ( ΔG) \| : \| [-7.05](http://www.premierbiosoft.com/NetPrimer/www.premierbiosoft.com) \| kcal/mol \| \| Hairpin ( ΔG) \| : \|  \| kcal/mol \| \| Repeats (# of pairs) \| : \|  \| kcal/mol \| \| Run (# of bases) \| : \| [4](http://www.premierbiosoft.com/NetPrimer/www.premierbiosoft.com) \| kcal/mol \| |

| \| Cross Dimer (ΔG) \| : \| [-4.89](http://www.premierbiosoft.com/NetPrimer/www.premierbiosoft.com) \| kcal/mol \| \| --- \| --- \| --- \| --- \| |
| --- | --- | --- | --- | --- |

|  |
| --- |

00000000000000000000000000000000000000000

| Pair 2: |  |  |  |  |  |
| --- | --- | --- | --- | --- | --- |
|  Left Primer 2:      | | | | | |
| Sequence: |  | | | | |
| Start:   533 | Length:   20 bp | Tm:   52.7 °C | GC:   50.0 % | ANY:   4.0 | SELF:   3.0 |
|  | | | | | |
|  Right Primer 2:      | | | | | |
| Sequence: |  | | | | |
| Start:   684 | Length:   23 bp | Tm:   59.1 °C | GC:   43.5 % | ANY:   7.0 | SELF:   1.0 |
|  | | | | | |
| Product Size:   152 bp | | Pair Any: 5.0 | Pair End: 3.0 |  |  |

| **Analysis Results #1: CAGTCTTACCTGGACTCTGA** | |
| --- | --- |
| \| Rating \| : \| 91.0 \|  \| \| --- \| --- \| --- \| --- \| \| Molecular Wt \| : \| 6068.04 \|  \| \| Tm \| : \| 49.2 \| °C \| \| GC% \| : \| 50.0 \|  \| \| GC Clamp \| : \| 1 \|  \| \| nmol/A_260_ \| : \| 5.4 \|  \| \| ug/A_260_ \| : \| 32.76 \|  \| \| ΔG \| : \| -28.72 \| kcal/mol \| | \| 3' end stability \| : \| -6.7 \| kcal/mol \| \| --- \| --- \| --- \| --- \| \| ΔH \| : \| -135.4 \| kcal/mol \| \| ΔS \| : \| -0.36 \| kcal/°K/mol \| \| 5' end ΔG \| : \| -6.47 \| kcal/mol \| \| Self Dimer ( ΔG) \| : \| [-4.55](http://www.premierbiosoft.com/NetPrimer/www.premierbiosoft.com) \| kcal/mol \| \| Hairpin ( ΔG) \| : \| [-0.42](http://www.premierbiosoft.com/NetPrimer/www.premierbiosoft.com) \| kcal/mol \| \| Repeats (# of pairs) \| : \|  \| kcal/mol \| \| Run (# of bases) \| : \|  \| kcal/mol \| |

| **Analysis Results #2: TGTTCTTGAGTGAAGGACTGAGA** | |
| --- | --- |
| \| Rating \| : \| 92.0 \|  \| \| --- \| --- \| --- \| --- \| \| Molecular Wt \| : \| 7158.74 \|  \| \| Tm \| : \| 56.64 \| °C \| \| GC% \| : \| 43.48 \|  \| \| GC Clamp \| : \| 1 \|  \| \| nmol/A_260_ \| : \| 4.32 \|  \| \| ug/A_260_ \| : \| 30.94 \|  \| \| ΔG \| : \| -33.68 \| kcal/mol \| | \| 3' end stability \| : \| -6.7 \| kcal/mol \| \| --- \| --- \| --- \| --- \| \| ΔH \| : \| -153.6 \| kcal/mol \| \| ΔS \| : \| -0.4 \| kcal/°K/mol \| \| 5' end ΔG \| : \| -6.82 \| kcal/mol \| \| Self Dimer ( ΔG) \| : \| [-4.17](http://www.premierbiosoft.com/NetPrimer/www.premierbiosoft.com) \| kcal/mol \| \| Hairpin ( ΔG) \| : \|  \| kcal/mol \| \| Repeats (# of pairs) \| : \|  \| kcal/mol \| \| Run (# of bases) \| : \|  \| kcal/mol \| |

| \| Cross Dimer (ΔG) \| : \| [-7.47](http://www.premierbiosoft.com/NetPrimer/www.premierbiosoft.com) \| kcal/mol \| \| --- \| --- \| --- \| --- \| |
| --- | --- | --- | --- | --- |

|  |
| --- |

000000000000000000000000000000000000000

|  | Forward Primer | Reverse Primer |
| --- | --- | --- |
| Sequence |  |  |
| Length |  |  |
| Start |  |  |
| Tm |  |  |
| GC |  |  |
| Tm |  |  |
| GC% |  |  |
| Self-Dimer ( ΔG) |  |  |
| Hairpin ( ΔG) |  |  |
| Cross Dimer (ΔG) |  | |
| Product size |  | |

| Pair 2: |  |  |  |  |  |
| --- | --- | --- | --- | --- | --- |
|  Left Primer 2:      | | | | | |
| Sequence: |  | | | | |
| Start:   533 | Length:   20 bp | Tm:   51.7 °C | GC:   50.0 % | ANY:   4.0 | SELF:   1.0 |
|  | | | | | |
|  Right Primer 2:      | | | | | |
| Sequence: |  | | | | |
| Start:   684 | Length:   23 bp | Tm:   59.1 °C | GC:   43.5 % | ANY:   7.0 | SELF:   1.0 |
|  | | | | | |
| Product Size:   152 bp | | Pair Any: 5.0 | Pair End: 3.0 |  |  |

| **Analysis Results #1: CAGTCTTACCTGGACTCTGT** | |
| --- | --- |
| \| Rating \| : \| 91.0 \|  \| \| --- \| --- \| --- \| --- \| \| Molecular Wt \| : \| 6059.03 \|  \| \| Tm \| : \| 48.29 \| °C \| \| GC% \| : \| 50.0 \|  \| \| GC Clamp \| : \| 1 \|  \| \| nmol/A_260_ \| : \| 5.56 \|  \| \| ug/A_260_ \| : \| 33.66 \|  \| \| ΔG \| : \| -28.49 \| kcal/mol \| | \| 3' end stability \| : \| -6.47 \| kcal/mol \| \| --- \| --- \| --- \| --- \| \| ΔH \| : \| -136.3 \| kcal/mol \| \| ΔS \| : \| -0.36 \| kcal/°K/mol \| \| 5' end ΔG \| : \| -6.47 \| kcal/mol \| \| Self Dimer ( ΔG) \| : \| [-4.55](http://www.premierbiosoft.com/NetPrimer/www.premierbiosoft.com) \| kcal/mol \| \| Hairpin ( ΔG) \| : \| [-0.42](http://www.premierbiosoft.com/NetPrimer/www.premierbiosoft.com) \| kcal/mol \| \| Repeats (# of pairs) \| : \|  \| kcal/mol \| \| Run (# of bases) \| : \|  \| kcal/mol \| |

| **Analysis Results #2: TGTTCTTGAGTGAAGGACTGAGA** | |
| --- | --- |
| \| Rating \| : \| 92.0 \|  \| \| --- \| --- \| --- \| --- \| \| Molecular Wt \| : \| 7158.74 \|  \| \| Tm \| : \| 56.64 \| °C \| \| GC% \| : \| 43.48 \|  \| \| GC Clamp \| : \| 1 \|  \| \| nmol/A_260_ \| : \| 4.32 \|  \| \| ug/A_260_ \| : \| 30.94 \|  \| \| ΔG \| : \| -33.68 \| kcal/mol \| | \| 3' end stability \| : \| -6.7 \| kcal/mol \| \| --- \| --- \| --- \| --- \| \| ΔH \| : \| -153.6 \| kcal/mol \| \| ΔS \| : \| -0.4 \| kcal/°K/mol \| \| 5' end ΔG \| : \| -6.82 \| kcal/mol \| \| Self Dimer ( ΔG) \| : \| [-4.17](http://www.premierbiosoft.com/NetPrimer/www.premierbiosoft.com) \| kcal/mol \| \| Hairpin ( ΔG) \| : \|  \| kcal/mol \| \| Repeats (# of pairs) \| : \|  \| kcal/mol \| \| Run (# of bases) \| : \|  \| kcal/mol \| |

| \| Cross Dimer (ΔG) \| : \| [-7.47](http://www.premierbiosoft.com/NetPrimer/www.premierbiosoft.com) \| kcal/mol \| \| --- \| --- \| --- \| --- \| |
| --- | --- | --- | --- | --- |
