# Supplementary material for: Investigation of pathogenic germline variants in gastric cancer and development of “GasCanBase” database
Source: Cancer Rep (Hoboken). 2023 Oct 22;6(12):e1906. doi: 10.1002/cnr2.1906 (PMC10728505; doi:10.1002/cnr2.1906)
Supplement: Supplementary file 1 — Data S1 Supporting Information. [file CNR2-6-e1906-s001.zip › Supplementary File/Table S6.12. Allele specific primer design on selected nsSNP of DCC gene.docx]

[rs116498325](https://www.ncbi.nlm.nih.gov/projects/SNP/snp_ref.cgi?rs=116498325) *[Homo sapiens]*

AATACAACACAGCCTGGGTCCCTTC[A/T]GCTCACTGTGGGAAACCTGAAGCCA

Chromosome: 18:53179000

Gene: DCC

1. Allele specific primer design on wild type nucleotide of DCC gene

| Primer Criteria | Forward Primer | Reverse Primer |
| --- | --- | --- |
| Sequence | ACACAGCCTGGGTCCCTTCA | TCTTCGCTTTTGTCACTTCG |
| Length | 20 bp | 20 bp |
| Start | 448 | 686 |
| Tm | 65.3 °C | 59.2 °C |
| GC | 60.0 % | 45.0 % |
| Tm | 62.32 °C | 56.49 °C |
| GC% | 60.0 | 45.0 |
| Self-Dimer ( ΔG) |  |  |
| Hairpin ( ΔG) |  |  |
| Cross Dimer (ΔG) |  | |
| Product size | 239 bp | |

1. Allele specific primer design on mutant nucleotide of DCC gene

| Primer Criteria | Forward Primer | Reverse Primer |
| --- | --- | --- |
| Sequence | ACACAGCCTGGGTCCCTTCT | TCTTCGCTTTTGTCACTTCG |
| Length | 20 bp | 20 bp |
| Start | 448 | 686 |
| Tm | 63.3 °C | 59.2 °C |
| GC | 60.0 % | 45.0 % |
| Tm | 60.65 °C | 56.49 °C |
| GC% | 60.0 | 45.0 |
| Self-Dimer ( ΔG) |  |  |
| Hairpin ( ΔG) |  |  |
| Cross Dimer (ΔG) |  | |
| Product size | 239 bp | |

| Pair 4: |  |  |  |  |  |
| --- | --- | --- | --- | --- | --- |
|  Left Primer 4:      | | | | | |
| Sequence: |  | | | | |
| Start:   448 | Length:   20 bp | Tm:   65.3 °C | GC:   60.0 % | ANY:   7.0 | SELF:   1.0 |
|  | | | | | |
|  Right Primer 4:      | | | | | |
| Sequence: |  | | | | |
| Start:   686 | Length:   20 bp | Tm:   59.2 °C | GC:   45.0 % | ANY:   3.0 | SELF:   2.0 |
|  | | | | | |
| Product Size:   239 bp | | Pair Any: 3.0 | Pair End: 0.0 |  |  |

| **Analysis Results #1: ACACAGCCTGGGTCCCTTCA** | |
| --- | --- |
| \| Rating \| : \| 100.0 \|  \| \| --- \| --- \| --- \| --- \| \| Molecular Wt \| : \| 6038.02 \|  \| \| Tm \| : \| 62.32 \| °C \| \| GC% \| : \| 60.0 \|  \| \| GC Clamp \| : \| 1 \|  \| \| nmol/A_260_ \| : \| 5.45 \|  \| \| ug/A_260_ \| : \| 32.92 \|  \| \| ΔG \| : \| -35.71 \| kcal/mol \| | \| 3' end stability \| : \| -7.07 \| kcal/mol \| \| --- \| --- \| --- \| --- \| \| ΔH \| : \| -152.5 \| kcal/mol \| \| ΔS \| : \| -0.39 \| kcal/°K/mol \| \| 5' end ΔG \| : \| -6.59 \| kcal/mol \| \| Self Dimer ( ΔG) \| : \|  \| kcal/mol \| \| Hairpin ( ΔG) \| : \|  \| kcal/mol \| \| Repeats (# of pairs) \| : \|  \| kcal/mol \| \| Run (# of bases) \| : \| [3](http://www.premierbiosoft.com/NetPrimer/www.premierbiosoft.com) \| kcal/mol \| |

| **Analysis Results #2: TCTTCGCTTTTGTCACTTCG** | |
| --- | --- |
| \| Rating \| : \| 100.0 \|  \| \| --- \| --- \| --- \| --- \| \| Molecular Wt \| : \| 6016.0 \|  \| \| Tm \| : \| 56.49 \| °C \| \| GC% \| : \| 45.0 \|  \| \| GC Clamp \| : \| 2 \|  \| \| nmol/A_260_ \| : \| 5.94 \|  \| \| ug/A_260_ \| : \| 35.75 \|  \| \| ΔG \| : \| -33.27 \| kcal/mol \| | \| 3' end stability \| : \| -8.73 \| kcal/mol \| \| --- \| --- \| --- \| --- \| \| ΔH \| : \| -150.8 \| kcal/mol \| \| ΔS \| : \| -0.39 \| kcal/°K/mol \| \| 5' end ΔG \| : \| -6.69 \| kcal/mol \| \| Self Dimer ( ΔG) \| : \|  \| kcal/mol \| \| Hairpin ( ΔG) \| : \|  \| kcal/mol \| \| Repeats (# of pairs) \| : \|  \| kcal/mol \| \| Run (# of bases) \| : \| [4](http://www.premierbiosoft.com/NetPrimer/www.premierbiosoft.com) \| kcal/mol \| |

| \| Cross Dimer (ΔG) \| : \|  \| kcal/mol \| \| --- \| --- \| --- \| --- \| |
| --- | --- | --- | --- | --- |

|  |
| --- |

| Pair 4: |  |  |  |  |  |
| --- | --- | --- | --- | --- | --- |
|  Left Primer 4:      | | | | | |
| Sequence: |  | | | | |
| Start:   448 | Length:   20 bp | Tm:   63.3 °C | GC:   60.0 % | ANY:   7.0 | SELF:   1.0 |
|  | | | | | |
|  Right Primer 4:      | | | | | |
| Sequence: |  | | | | |
| Start:   686 | Length:   20 bp | Tm:   59.2 °C | GC:   45.0 % | ANY:   3.0 | SELF:   2.0 |
|  | | | | | |
| Product Size:   239 bp | | Pair Any: 3.0 | Pair End: 0.0 |  |  |

| **Analysis Results #1: ACACAGCCTGGGTCCCTTCT** | |
| --- | --- |
| \| Rating \| : \| 100.0 \|  \| \| --- \| --- \| --- \| --- \| \| Molecular Wt \| : \| 6029.01 \|  \| \| Tm \| : \| 60.65 \| °C \| \| GC% \| : \| 60.0 \|  \| \| GC Clamp \| : \| 1 \|  \| \| nmol/A_260_ \| : \| 5.64 \|  \| \| ug/A_260_ \| : \| 33.99 \|  \| \| ΔG \| : \| -35.36 \| kcal/mol \| | \| 3' end stability \| : \| -6.72 \| kcal/mol \| \| --- \| --- \| --- \| --- \| \| ΔH \| : \| -154.5 \| kcal/mol \| \| ΔS \| : \| -0.4 \| kcal/°K/mol \| \| 5' end ΔG \| : \| -6.59 \| kcal/mol \| \| Self Dimer ( ΔG) \| : \|  \| kcal/mol \| \| Hairpin ( ΔG) \| : \|  \| kcal/mol \| \| Repeats (# of pairs) \| : \|  \| kcal/mol \| \| Run (# of bases) \| : \| [3](http://www.premierbiosoft.com/NetPrimer/www.premierbiosoft.com) \| kcal/mol \| |

| **Analysis Results #2: TCTTCGCTTTTGTCACTTCG** | |
| --- | --- |
| \| Rating \| : \| 100.0 \|  \| \| --- \| --- \| --- \| --- \| \| Molecular Wt \| : \| 6016.0 \|  \| \| Tm \| : \| 56.49 \| °C \| \| GC% \| : \| 45.0 \|  \| \| GC Clamp \| : \| 2 \|  \| \| nmol/A_260_ \| : \| 5.94 \|  \| \| ug/A_260_ \| : \| 35.75 \|  \| \| ΔG \| : \| -33.27 \| kcal/mol \| | \| 3' end stability \| : \| -8.73 \| kcal/mol \| \| --- \| --- \| --- \| --- \| \| ΔH \| : \| -150.8 \| kcal/mol \| \| ΔS \| : \| -0.39 \| kcal/°K/mol \| \| 5' end ΔG \| : \| -6.69 \| kcal/mol \| \| Self Dimer ( ΔG) \| : \|  \| kcal/mol \| \| Hairpin ( ΔG) \| : \|  \| kcal/mol \| \| Repeats (# of pairs) \| : \|  \| kcal/mol \| \| Run (# of bases) \| : \| [4](http://www.premierbiosoft.com/NetPrimer/www.premierbiosoft.com) \| kcal/mol \| |

| \| Cross Dimer (ΔG) \| : \|  \| kcal/mol \| \| --- \| --- \| --- \| --- \| |
| --- | --- | --- | --- | --- |

|  |
| --- |
