# Supplementary material for: Investigation of pathogenic germline variants in gastric cancer and development of “GasCanBase” database
Source: Cancer Rep (Hoboken). 2023 Oct 22;6(12):e1906. doi: 10.1002/cnr2.1906 (PMC10728505; doi:10.1002/cnr2.1906)
Supplement: Supplementary file 1 — Data S1 Supporting Information. [file CNR2-6-e1906-s001.zip › Supplementary File/Table S6.13. Allele specific primer design on selected nsSNP of EPCAM gene.docx]

[rs115283528](https://www.ncbi.nlm.nih.gov/projects/SNP/snp_ref.cgi?rs=115283528) *[Homo sapiens]*

TTGCTGTTATTGTGGTTGTGGTGAT[A/G]GCAGTTGTTGCTGGAATTGTTGTGC

Chromosome: 2:47379942

Gene: EPCAM

1. Allele specific primer design on wild type nucleotide of EPCAM gene

|  | Forward Primer | Reverse Primer |
| --- | --- | --- |
| Sequence | GTTATTGTGGTTGTGGTGATA | GTCTCAGCCTCCCAAAGTGT |
| Length | 21 bp | 20 bp |
| Start | 567 | 731 |
| Tm | 53.4 °C | 59.3 °C |
| GC | 38.1 % | 55.0 % |
| Tm | 50.65 °C | 56.32 °C |
| GC% | 38.1 | 55.0 |
| Self-Dimer ( ΔG) | -3.44 kcal/mol |  |
| Hairpin ( ΔG) |  |  |
| Cross Dimer (ΔG) | -4.53 kcal/mol | |
| Product size | 165 bp | |

2. Allele specific primer design on mutant nucleotide of EPCAM gene

|  | Forward Primer | Reverse Primer |
| --- | --- | --- |
| Sequence | GTTATTGTGGTTGTGGTGATG | GTCTCAGCCTCCCAAAGTGT |
| Length | 21 bp | 20bp |
| Start | 567 |  |
| Tm | 56.2 °C | 59.3 °C |
| GC | 42.9 % | 55.0 % |
| Tm | 53.32 °C | 56.32 °C |
| GC% | 42.86 | 55.0 |
| Self-Dimer ( ΔG) |  |  |
| Hairpin ( ΔG) |  |  |
| Cross Dimer (ΔG) | -4.53 kcal/mol | |
| Product size | 165 bp | |

| Pair 4: |  |  |  |  |  |
| --- | --- | --- | --- | --- | --- |
|  Left Primer 4:      | | | | | |
| Sequence: |  | | | | |
| Start:   567 | Length:   21 bp | Tm:   53.4 °C | GC:   38.1 % | ANY:   3.0 | SELF:   2.0 |
|  | | | | | |
|  Right Primer 4:      | | | | | |
| Sequence: |  | | | | |
| Start:   731 | Length:   20 bp | Tm:   59.3 °C | GC:   55.0 % | ANY:   3.0 | SELF:   1.0 |
|  | | | | | |
| Product Size:   165 bp | | Pair Any: 3.0 | Pair End: 1.0 |  |  |

| **Analysis Results #1: GTTATTGTGGTTGTGGTGATA** | |
| --- | --- |
| \| Rating \| : \| 93.0 \|  \| \| --- \| --- \| --- \| --- \| \| Molecular Wt \| : \| 6553.33 \|  \| \| Tm \| : \| 50.65 \| °C \| \| GC% \| : \| 38.1 \|  \| \| GC Clamp \| : \| 1 \|  \| \| nmol/A_260_ \| : \| 4.81 \|  \| \| ug/A_260_ \| : \| 31.52 \|  \| \| ΔG \| : \| -30.39 \| kcal/mol \| | \| 3' end stability \| : \| -5.96 \| kcal/mol \| \| --- \| --- \| --- \| --- \| \| ΔH \| : \| -145.6 \| kcal/mol \| \| ΔS \| : \| -0.39 \| kcal/°K/mol \| \| 5' end ΔG \| : \| -5.72 \| kcal/mol \| \| Self Dimer ( ΔG) \| : \| [-3.44](http://www.premierbiosoft.com/NetPrimer/www.premierbiosoft.com) \| kcal/mol \| \| Hairpin ( ΔG) \| : \|  \| kcal/mol \| \| Repeats (# of pairs) \| : \|  \| kcal/mol \| \| Run (# of bases) \| : \|  \| kcal/mol \| |

| **Analysis Results #2: GTCTCAGCCTCCCAAAGTGT** | |
| --- | --- |
| \| Rating \| : \| 100.0 \|  \| \| --- \| --- \| --- \| --- \| \| Molecular Wt \| : \| 6053.03 \|  \| \| Tm \| : \| 56.32 \| °C \| \| GC% \| : \| 55.0 \|  \| \| GC Clamp \| : \| 1 \|  \| \| nmol/A_260_ \| : \| 5.44 \|  \| \| ug/A_260_ \| : \| 32.91 \|  \| \| ΔG \| : \| -32.74 \| kcal/mol \| | \| 3' end stability \| : \| -6.24 \| kcal/mol \| \| --- \| --- \| --- \| --- \| \| ΔH \| : \| -147.2 \| kcal/mol \| \| ΔS \| : \| -0.38 \| kcal/°K/mol \| \| 5' end ΔG \| : \| -6.09 \| kcal/mol \| \| Self Dimer ( ΔG) \| : \|  \| kcal/mol \| \| Hairpin ( ΔG) \| : \|  \| kcal/mol \| \| Repeats (# of pairs) \| : \|  \| kcal/mol \| \| Run (# of bases) \| : \| [3](http://www.premierbiosoft.com/NetPrimer/www.premierbiosoft.com) \| kcal/mol \| |

| \| Cross Dimer (ΔG) \| : \| [-4.53](http://www.premierbiosoft.com/NetPrimer/www.premierbiosoft.com) \| kcal/mol \| \| --- \| --- \| --- \| --- \| |
| --- | --- | --- | --- | --- |

| Pair 4: |  |  |  |  |  |
| --- | --- | --- | --- | --- | --- |
|  Left Primer 4:      | | | | | |
| Sequence: |  | | | | |
| Start:   567 | Length:   21 bp | Tm:   56.2 °C | GC:   42.9 % | ANY:   2.0 | SELF:   0.0 |
|  | | | | | |
|  Right Primer 4:      | | | | | |
| Sequence: |  | | | | |
| Start:   731 | Length:   20 bp | Tm:   59.3 °C | GC:   55.0 % | ANY:   3.0 | SELF:   1.0 |
|  | | | | | |
| Product Size:   165 bp | |  |  |  |  |

| **Analysis Results #1: GTTATTGTGGTTGTGGTGATG** | |
| --- | --- |
| \| Rating \| : \| 100.0 \|  \| \| --- \| --- \| --- \| --- \| \| Molecular Wt \| : \| 6569.33 \|  \| \| Tm \| : \| 53.32 \| °C \| \| GC% \| : \| 42.86 \|  \| \| GC Clamp \| : \| 1 \|  \| \| nmol/A_260_ \| : \| 4.91 \|  \| \| ug/A_260_ \| : \| 32.28 \|  \| \| ΔG \| : \| -31.39 \| kcal/mol \| | \| 3' end stability \| : \| -6.96 \| kcal/mol \| \| --- \| --- \| --- \| --- \| \| ΔH \| : \| -145.4 \| kcal/mol \| \| ΔS \| : \| -0.38 \| kcal/°K/mol \| \| 5' end ΔG \| : \| -5.72 \| kcal/mol \| \| Self Dimer ( ΔG) \| : \|  \| kcal/mol \| \| Hairpin ( ΔG) \| : \|  \| kcal/mol \| \| Repeats (# of pairs) \| : \|  \| kcal/mol \| \| Run (# of bases) \| : \|  \| kcal/mol \| |

| **Analysis Results #2: GTCTCAGCCTCCCAAAGTGT** | |
| --- | --- |
| \| Rating \| : \| 100.0 \|  \| \| --- \| --- \| --- \| --- \| \| Molecular Wt \| : \| 6053.03 \|  \| \| Tm \| : \| 56.32 \| °C \| \| GC% \| : \| 55.0 \|  \| \| GC Clamp \| : \| 1 \|  \| \| nmol/A_260_ \| : \| 5.44 \|  \| \| ug/A_260_ \| : \| 32.91 \|  \| \| ΔG \| : \| -32.74 \| kcal/mol \| | \| 3' end stability \| : \| -6.24 \| kcal/mol \| \| --- \| --- \| --- \| --- \| \| ΔH \| : \| -147.2 \| kcal/mol \| \| ΔS \| : \| -0.38 \| kcal/°K/mol \| \| 5' end ΔG \| : \| -6.09 \| kcal/mol \| \| Self Dimer ( ΔG) \| : \|  \| kcal/mol \| \| Hairpin ( ΔG) \| : \|  \| kcal/mol \| \| Repeats (# of pairs) \| : \|  \| kcal/mol \| \| Run (# of bases) \| : \| [3](http://www.premierbiosoft.com/NetPrimer/www.premierbiosoft.com) \| kcal/mol \| |

| \| Cross Dimer (ΔG) \| : \| [-4.53](http://www.premierbiosoft.com/NetPrimer/www.premierbiosoft.com) \| kcal/mol \| \| --- \| --- \| --- \| --- \| |
| --- | --- | --- | --- | --- |
