# Supplementary material for: Investigation of pathogenic germline variants in gastric cancer and development of “GasCanBase” database
Source: Cancer Rep (Hoboken). 2023 Oct 22;6(12):e1906. doi: 10.1002/cnr2.1906 (PMC10728505; doi:10.1002/cnr2.1906)
Supplement: Supplementary file 1 — Data S1 Supporting Information. [file CNR2-6-e1906-s001.zip › Supplementary File/Table S6.14. Allele specific primer design on selected nsSNP of KIT gene.docx]

[rs121913507](https://www.ncbi.nlm.nih.gov/projects/SNP/snp_ref.cgi?rs=121913507) *[Homo sapiens]*

ATTTGTGATTTTGGTCTAGCCAGAG[A/T]CATCAAGAATGATTCTAATTATGTG

Chromosome: 4:54733155

Gene: KIT

1. Allele specific primer design on wild type nucleotide of KIT gene

|  | Forward Primer | Reverse Primer |
| --- | --- | --- |
| Sequence | GATTTTGGTCTAGCCAGAGA | TGTTCAGCATACCATGCAAAT |
| Length | 20 bp | 21 bp |
| Start | 535 | 759 |
| Tm | 54.6 °C | 59.1 °C |
| GC | 45.0 % | 38.1 % |
| Tm | 51.86 °C | 56.57 °C |
| GC% | 45.0 | 38.1 |
| Self-Dimer ( ΔG) | -4.17 kcal/mol | -8.05 kcal/mol |
| Hairpin ( ΔG) | -0.62 kcal/mol | -1.37 kcal/mol |
| Cross Dimer (ΔG) | -6.84 kcal/mol | |
| Product size | 225 bp | |

|  |  |  |  |  |  |
| --- | --- | --- | --- | --- | --- |
| 2. Allele specific primer design on mutant nucleotide of KIT gene |  |  |  |  |  |

|  | Forward Primer | Reverse Primer |
| --- | --- | --- |
| Sequence | GATTTTGGTCTAGCCAGAGT | TGTTCAGCATACCATGCAAAT |
| Length | 20 bp | 21bp |
| Start | 535 | 759 |
| Tm | 53.6 °C | 59.1 °C |
| GC | 45.0 % | 38.1 % |
| Tm | 50.99 °C | 56.57 °C |
| GC% | 45.0 | 38.1 |
| Self-Dimer ( ΔG) | -4.17  kcal/mol | -8.05  kcal/mol |
| Hairpin ( ΔG) | -0.62  kcal/mol | -1.37  kcal/mol |
| Cross Dimer (ΔG) | -6.84  kcal/mol | |
| Product size | 225 bp | |

|  |  |  |  |  |  |
| --- | --- | --- | --- | --- | --- |
|  Left Primer 3:      | | | | | |
| Sequence: |  | | | | |
| Start:   535 | Length:   20 bp | Tm:   54.6 °C | GC:   45.0 % | ANY:   6.0 | SELF:   4.0 |
|  | | | | | |
|  Right Primer 3:      | | | | | |
| Sequence: |  | | | | |
| Start:   759 | Length:   21 bp | Tm:   59.1 °C | GC:   38.1 % | ANY:   5.0 | SELF:   2.0 |
|  | | | | | |
| Product Size:   225 bp | | Pair Any: 4.0 | Pair End: 1.0 |  |  |

| **Analysis Results #1: GATTTTGGTCTAGCCAGAGA** | |
| --- | --- |
| \| Rating \| : \| 91.0 \|  \| \| --- \| --- \| --- \| --- \| \| Molecular Wt \| : \| 6172.1 \|  \| \| Tm \| : \| 51.86 \| °C \| \| GC% \| : \| 45.0 \|  \| \| GC Clamp \| : \| 1 \|  \| \| nmol/A_260_ \| : \| 5.02 \|  \| \| ug/A_260_ \| : \| 31.0 \|  \| \| ΔG \| : \| -30.99 \| kcal/mol \| | \| 3' end stability \| : \| -6.35 \| kcal/mol \| \| --- \| --- \| --- \| --- \| \| ΔH \| : \| -146.7 \| kcal/mol \| \| ΔS \| : \| -0.39 \| kcal/°K/mol \| \| 5' end ΔG \| : \| -6.94 \| kcal/mol \| \| Self Dimer ( ΔG) \| : \| [-4.17](http://www.premierbiosoft.com/NetPrimer/www.premierbiosoft.com) \| kcal/mol \| \| Hairpin ( ΔG) \| : \| [-0.62](http://www.premierbiosoft.com/NetPrimer/www.premierbiosoft.com) \| kcal/mol \| \| Repeats (# of pairs) \| : \|  \| kcal/mol \| \| Run (# of bases) \| : \| [4](http://www.premierbiosoft.com/NetPrimer/www.premierbiosoft.com) \| kcal/mol \| |

| **Analysis Results #2: TGTTCAGCATACCATGCAAAT** | |
| --- | --- |
| \| Rating \| : \| 83.0 \|  \| \| --- \| --- \| --- \| --- \| \| Molecular Wt \| : \| 6389.27 \|  \| \| Tm \| : \| 56.57 \| °C \| \| GC% \| : \| 38.1 \|  \| \| GC Clamp \| : \| 1 \|  \| \| nmol/A_260_ \| : \| 4.89 \|  \| \| ug/A_260_ \| : \| 31.27 \|  \| \| ΔG \| : \| -33.64 \| kcal/mol \| | \| 3' end stability \| : \| -7.32 \| kcal/mol \| \| --- \| --- \| --- \| --- \| \| ΔH \| : \| -153.5 \| kcal/mol \| \| ΔS \| : \| -0.4 \| kcal/°K/mol \| \| 5' end ΔG \| : \| -6.82 \| kcal/mol \| \| Self Dimer ( ΔG) \| : \| [-8.05](http://www.premierbiosoft.com/NetPrimer/www.premierbiosoft.com) \| kcal/mol \| \| Hairpin ( ΔG) \| : \| [-1.37](http://www.premierbiosoft.com/NetPrimer/www.premierbiosoft.com) \| kcal/mol \| \| Repeats (# of pairs) \| : \|  \| kcal/mol \| \| Run (# of bases) \| : \| [3](http://www.premierbiosoft.com/NetPrimer/www.premierbiosoft.com) \| kcal/mol \| |

| \| Cross Dimer (ΔG) \| : \| [-6.84](http://www.premierbiosoft.com/NetPrimer/www.premierbiosoft.com) \| kcal/mol \| \| --- \| --- \| --- \| --- \| |
| --- | --- | --- | --- | --- |

|  |
| --- |

| Pair 2: |  |  |  |  |  |
| --- | --- | --- | --- | --- | --- |
|  Left Primer 2:      | | | | | |
| Sequence: |  | | | | |
| Start:   535 | Length:   20 bp | Tm:   53.6 °C | GC:   45.0 % | ANY:   6.0 | SELF:   6.0 |
|  | | | | | |
|  Right Primer 2:      | | | | | |
| Sequence: |  | | | | |
| Start:   759 | Length:   21 bp | Tm:   59.1 °C | GC:   38.1 % | ANY:   5.0 | SELF:   2.0 |
|  | | | | | |
| Product Size:   225 bp | | Pair Any: 4.0 | Pair End: 1.0 |  |  |

| **Analysis Results #1: GATTTTGGTCTAGCCAGAGT** | |
| --- | --- |
| \| Rating \| : \| 91.0 \|  \| \| --- \| --- \| --- \| --- \| \| Molecular Wt \| : \| 6163.09 \|  \| \| Tm \| : \| 50.99 \| °C \| \| GC% \| : \| 45.0 \|  \| \| GC Clamp \| : \| 1 \|  \| \| nmol/A_260_ \| : \| 5.16 \|  \| \| ug/A_260_ \| : \| 31.78 \|  \| \| ΔG \| : \| -30.76 \| kcal/mol \| | \| 3' end stability \| : \| -6.11 \| kcal/mol \| \| --- \| --- \| --- \| --- \| \| ΔH \| : \| -147.6 \| kcal/mol \| \| ΔS \| : \| -0.39 \| kcal/°K/mol \| \| 5' end ΔG \| : \| -6.94 \| kcal/mol \| \| Self Dimer ( ΔG) \| : \| [-4.17](http://www.premierbiosoft.com/NetPrimer/www.premierbiosoft.com) \| kcal/mol \| \| Hairpin ( ΔG) \| : \| [-0.62](http://www.premierbiosoft.com/NetPrimer/www.premierbiosoft.com) \| kcal/mol \| \| Repeats (# of pairs) \| : \|  \| kcal/mol \| \| Run (# of bases) \| : \| [4](http://www.premierbiosoft.com/NetPrimer/www.premierbiosoft.com) \| kcal/mol \| |

| **Analysis Results #2: TGTTCAGCATACCATGCAAAT** | |
| --- | --- |
| \| Rating \| : \| 83.0 \|  \| \| --- \| --- \| --- \| --- \| \| Molecular Wt \| : \| 6389.27 \|  \| \| Tm \| : \| 56.57 \| °C \| \| GC% \| : \| 38.1 \|  \| \| GC Clamp \| : \| 1 \|  \| \| nmol/A_260_ \| : \| 4.89 \|  \| \| ug/A_260_ \| : \| 31.27 \|  \| \| ΔG \| : \| -33.64 \| kcal/mol \| | \| 3' end stability \| : \| -7.32 \| kcal/mol \| \| --- \| --- \| --- \| --- \| \| ΔH \| : \| -153.5 \| kcal/mol \| \| ΔS \| : \| -0.4 \| kcal/°K/mol \| \| 5' end ΔG \| : \| -6.82 \| kcal/mol \| \| Self Dimer ( ΔG) \| : \| [-8.05](http://www.premierbiosoft.com/NetPrimer/www.premierbiosoft.com) \| kcal/mol \| \| Hairpin ( ΔG) \| : \| [-1.37](http://www.premierbiosoft.com/NetPrimer/www.premierbiosoft.com) \| kcal/mol \| \| Repeats (# of pairs) \| : \|  \| kcal/mol \| \| Run (# of bases) \| : \| [3](http://www.premierbiosoft.com/NetPrimer/www.premierbiosoft.com) \| kcal/mol \| |

| \| Cross Dimer (ΔG) \| : \| [-6.84](http://www.premierbiosoft.com/NetPrimer/www.premierbiosoft.com) \| kcal/mol \| \| --- \| --- \| --- \| --- \| |
| --- | --- | --- | --- | --- |
