# Supplementary material for: Investigation of pathogenic germline variants in gastric cancer and development of “GasCanBase” database
Source: Cancer Rep (Hoboken). 2023 Oct 22;6(12):e1906. doi: 10.1002/cnr2.1906 (PMC10728505; doi:10.1002/cnr2.1906)
Supplement: Supplementary file 1 — Data S1 Supporting Information. [file CNR2-6-e1906-s001.zip › Supplementary File/Table S6.15. Allele specific primer design on selected nsSNP of KITLG gene.docx]

[rs41416044](https://www.ncbi.nlm.nih.gov/projects/SNP/snp_ref.cgi?rs=41416044) *[Homo sapiens]*

GCCACTACAAAGTCCTTGAAGGCAT[A/C/T]AATGGATCTATTAAAAATTCTAAAG

Chromosome: 12:88516409

Gene: KITLG

1. Allele specific primer design on wild type nucleotide of KITLG gene

|  | Forward Primer | Reverse Primer |
| --- | --- | --- |
| Sequence | ACAAAGTCCTTGAAGGCATC | CAGCCATCTAAAAGTGCTAGTTGT |
| Length | 20 bp | 24 bp |
| Start | 497 | 729 |
| Tm | 56.8 °C | 59.1 °C |
| GC | 45.0 % | 41.7 % |
| Tm | 54.12 °C | 58.12 °C |
| GC% | 45.0 | 41.67 |
| Self-Dimer ( ΔG) | -6.61  kcal/mol | -4.16  kcal/mol |
| Hairpin ( ΔG) |  | -0.24  kcal/mol |
| Cross Dimer (ΔG) | -6.24  kcal/mol | |
| Product size | 233 bp | |

1. Allele specific primer design on mutant nucleotide of KITLG gene

|  | Forward Primer | Reverse Primer |
| --- | --- | --- |
| Sequence | ACAAAGTCCTTGAAGGCATA | CAGCCATCTAAAAGTGCTAGTTGT |
| Length | 20 bp | 24bp |
| Start | 497 | 729 |
| Tm | 55.0 °C | 59.1 °C |
| GC | 40.0 % | 41.7 % |
| Tm | 52.41 °C | 58.12 °C |
| GC% | 40.0 | 41.67 |
| Self-Dimer ( ΔG) | -6.61  kcal/mol | -4.16  kcal/mol |
| Hairpin ( ΔG) |  | -0.24  kcal/mol |
| Cross Dimer (ΔG) | -6.24  kcal/mol | |
| Product size | 233 bp | |

|  | Forward Primer | Reverse Primer |
| --- | --- | --- |
| Sequence | ACAAAGTCCTTGAAGGCATT | CAGCCATCTAAAAGTGCTAGTTGT |
| Length | 20 bp | 24 bp |
| Start | 497 | 729 |
| Tm | 56.4 °C | 59.1 °C |
| GC | 40.0 % | 41.7 % |
| Tm | 53.97 °C | 58.12 °C |
| GC% | 40.0 | 41.67 |
| Self-Dimer ( ΔG) | -6.61  kcal/mol | -4.16  kcal/mol |
| Hairpin ( ΔG) |  | -0.24  kcal/mol |
| Cross Dimer (ΔG) | -6.24  kcal/mol | |
| Product size | 233 bp | |

| Pair 2: |  |  |  |  |  |
| --- | --- | --- | --- | --- | --- |
|  Left Primer 2:      | | | | | |
| Sequence: |  | | | | |
| Start:   497 | Length:   20 bp | Tm:   56.8 °C | GC:   45.0 % | ANY:   7.0 | SELF:   3.0 |
|  | | | | | |
|  Right Primer 2:      | | | | | |
| Sequence: |  | | | | |
| Start:   729 | Length:   24 bp | Tm:   59.1 °C | GC:   41.7 % | ANY:   5.0 | SELF:   3.0 |
|  | | | | | |
| Product Size:   233 bp | | Pair Any: 4.0 | Pair End: 1.0 |  |  |

| **Analysis Results #1: ACAAAGTCCTTGAAGGCATC** | |
| --- | --- |
| \| Rating \| : \| 88.0 \|  \| \| --- \| --- \| --- \| --- \| \| Molecular Wt \| : \| 6110.08 \|  \| \| Tm \| : \| 54.12 \| °C \| \| GC% \| : \| 45.0 \|  \| \| GC Clamp \| : \| 2 \|  \| \| nmol/A_260_ \| : \| 5.05 \|  \| \| ug/A_260_ \| : \| 30.83 \|  \| \| ΔG \| : \| -32.09 \| kcal/mol \| | \| 3' end stability \| : \| -8.14 \| kcal/mol \| \| --- \| --- \| --- \| --- \| \| ΔH \| : \| -148.7 \| kcal/mol \| \| ΔS \| : \| -0.39 \| kcal/°K/mol \| \| 5' end ΔG \| : \| -7.18 \| kcal/mol \| \| Self Dimer ( ΔG) \| : \| [-6.61](http://www.premierbiosoft.com/NetPrimer/www.premierbiosoft.com) \| kcal/mol \| \| Hairpin ( ΔG) \| : \|  \| kcal/mol \| \| Repeats (# of pairs) \| : \|  \| kcal/mol \| \| Run (# of bases) \| : \| [3](http://www.premierbiosoft.com/NetPrimer/www.premierbiosoft.com) \| kcal/mol \| |

| **Analysis Results #2: CAGCCATCTAAAAGTGCTAGTTGT** | |
| --- | --- |
| \| Rating \| : \| 92.0 \|  \| \| --- \| --- \| --- \| --- \| \| Molecular Wt \| : \| 7351.89 \|  \| \| Tm \| : \| 58.12 \| °C \| \| GC% \| : \| 41.67 \|  \| \| GC Clamp \| : \| 1 \|  \| \| nmol/A_260_ \| : \| 4.29 \|  \| \| ug/A_260_ \| : \| 31.51 \|  \| \| ΔG \| : \| -37.43 \| kcal/mol \| | \| 3' end stability \| : \| -6.58 \| kcal/mol \| \| --- \| --- \| --- \| --- \| \| ΔH \| : \| -177.5 \| kcal/mol \| \| ΔS \| : \| -0.47 \| kcal/°K/mol \| \| 5' end ΔG \| : \| -9.76 \| kcal/mol \| \| Self Dimer ( ΔG) \| : \| [-4.16](http://www.premierbiosoft.com/NetPrimer/www.premierbiosoft.com) \| kcal/mol \| \| Hairpin ( ΔG) \| : \| [-0.24](http://www.premierbiosoft.com/NetPrimer/www.premierbiosoft.com) \| kcal/mol \| \| Repeats (# of pairs) \| : \|  \| kcal/mol \| \| Run (# of bases) \| : \| [4](http://www.premierbiosoft.com/NetPrimer/www.premierbiosoft.com) \| kcal/mol \| |

| \| Cross Dimer (ΔG) \| : \| [-6.24](http://www.premierbiosoft.com/NetPrimer/www.premierbiosoft.com) \| kcal/mol \| \| --- \| --- \| --- \| --- \| |
| --- | --- | --- | --- | --- |

| Pair 2: |  |  |  |  |  |
| --- | --- | --- | --- | --- | --- |
|  Left Primer 2:      | | | | | |
| Sequence: |  | | | | |
| Start:   497 | Length:   20 bp | Tm:   55.0 °C | GC:   40.0 % | ANY:   7.0 | SELF:   3.0 |
|  | | | | | |
|  Right Primer 2:      | | | | | |
| Sequence: |  | | | | |
| Start:   729 | Length:   24 bp | Tm:   59.1 °C | GC:   41.7 % | ANY:   5.0 | SELF:   3.0 |
|  | | | | | |
| Product Size:   233 bp | | Pair Any: 4.0 | Pair End: 1.0 |  |  |

| **Analysis Results #1: ACAAAGTCCTTGAAGGCATA** | |
| --- | --- |
| \| Rating \| : \| 88.0 \|  \| \| --- \| --- \| --- \| --- \| \| Molecular Wt \| : \| 6134.1 \|  \| \| Tm \| : \| 52.41 \| °C \| \| GC% \| : \| 40.0 \|  \| \| GC Clamp \| : \| 2 \|  \| \| nmol/A_260_ \| : \| 4.87 \|  \| \| ug/A_260_ \| : \| 29.86 \|  \| \| ΔG \| : \| -31.48 \| kcal/mol \| | \| 3' end stability \| : \| -7.53 \| kcal/mol \| \| --- \| --- \| --- \| --- \| \| ΔH \| : \| -149.1 \| kcal/mol \| \| ΔS \| : \| -0.39 \| kcal/°K/mol \| \| 5' end ΔG \| : \| -7.18 \| kcal/mol \| \| Self Dimer ( ΔG) \| : \| [-6.61](http://www.premierbiosoft.com/NetPrimer/www.premierbiosoft.com) \| kcal/mol \| \| Hairpin ( ΔG) \| : \|  \| kcal/mol \| \| Repeats (# of pairs) \| : \|  \| kcal/mol \| \| Run (# of bases) \| : \| [3](http://www.premierbiosoft.com/NetPrimer/www.premierbiosoft.com) \| kcal/mol \| |

| **Analysis Results #2: CAGCCATCTAAAAGTGCTAGTTGT** | |
| --- | --- |
| \| Rating \| : \| 92.0 \|  \| \| --- \| --- \| --- \| --- \| \| Molecular Wt \| : \| 7351.89 \|  \| \| Tm \| : \| 58.12 \| °C \| \| GC% \| : \| 41.67 \|  \| \| GC Clamp \| : \| 1 \|  \| \| nmol/A_260_ \| : \| 4.29 \|  \| \| ug/A_260_ \| : \| 31.51 \|  \| \| ΔG \| : \| -37.43 \| kcal/mol \| | \| 3' end stability \| : \| -6.58 \| kcal/mol \| \| --- \| --- \| --- \| --- \| \| ΔH \| : \| -177.5 \| kcal/mol \| \| ΔS \| : \| -0.47 \| kcal/°K/mol \| \| 5' end ΔG \| : \| -9.76 \| kcal/mol \| \| Self Dimer ( ΔG) \| : \| [-4.16](http://www.premierbiosoft.com/NetPrimer/www.premierbiosoft.com) \| kcal/mol \| \| Hairpin ( ΔG) \| : \| [-0.24](http://www.premierbiosoft.com/NetPrimer/www.premierbiosoft.com) \| kcal/mol \| \| Repeats (# of pairs) \| : \|  \| kcal/mol \| \| Run (# of bases) \| : \| [4](http://www.premierbiosoft.com/NetPrimer/www.premierbiosoft.com) \| kcal/mol \| |

| \| Cross Dimer (ΔG) \| : \| [-6.24](http://www.premierbiosoft.com/NetPrimer/www.premierbiosoft.com) \| kcal/mol \| \| --- \| --- \| --- \| --- \| |
| --- | --- | --- | --- | --- |

| Pair 2: |  |  |  | |  |  |  |
| --- | --- | --- | --- | --- | --- | --- | --- |
|  Left Primer 2:      | | | | | | |  |
| Sequence: |  | | | | | |  |
| Start:   497 | Length:   20 bp | Tm:   56.4 °C | GC:   40.0 % | | ANY:   7.0 | SELF:   7.0 |  |
|  | | | | | | |  |
|  Right Primer 2:      | | | | | | |  |
| Sequence: |  | | | | | |  |
| Start:   729 | Length:   24 bp | Tm:   59.1 °C | GC:   41.7 % | | ANY:   5.0 | SELF:   3.0 |  |
|  | | | | | | |  |
| Product Size:   233 bp | | Pair Any: 4.0 | Pair End: 0.0 | |  |  |  |
| **Analysis Results #1: ACAAAGTCCTTGAAGGCATT** | | | | | | | |
| \| Rating \| : \| 88.0 \|  \| \| --- \| --- \| --- \| --- \| \| Molecular Wt \| : \| 6125.09 \|  \| \| Tm \| : \| 53.97 \| °C \| \| GC% \| : \| 40.0 \|  \| \| GC Clamp \| : \| 2 \|  \| \| nmol/A_260_ \| : \| 5.03 \|  \| \| ug/A_260_ \| : \| 30.81 \|  \| \| ΔG \| : \| -32.46 \| kcal/mol \| | | | | \| 3' end stability \| : \| -8.51 \| kcal/mol \| \| --- \| --- \| --- \| --- \| \| ΔH \| : \| -152.2 \| kcal/mol \| \| ΔS \| : \| -0.4 \| kcal/°K/mol \| \| 5' end ΔG \| : \| -7.18 \| kcal/mol \| \| Self Dimer ( ΔG) \| : \| [-6.61](http://www.premierbiosoft.com/NetPrimer/www.premierbiosoft.com) \| kcal/mol \| \| Hairpin ( ΔG) \| : \|  \| kcal/mol \| \| Repeats (# of pairs) \| : \|  \| kcal/mol \| \| Run (# of bases) \| : \| [3](http://www.premierbiosoft.com/NetPrimer/www.premierbiosoft.com) \| kcal/mol \| | | | |

| **Analysis Results #2: CAGCCATCTAAAAGTGCTAGTTGT** | |
| --- | --- |
| \| Rating \| : \| 92.0 \|  \| \| --- \| --- \| --- \| --- \| \| Molecular Wt \| : \| 7351.89 \|  \| \| Tm \| : \| 58.12 \| °C \| \| GC% \| : \| 41.67 \|  \| \| GC Clamp \| : \| 1 \|  \| \| nmol/A_260_ \| : \| 4.29 \|  \| \| ug/A_260_ \| : \| 31.51 \|  \| \| ΔG \| : \| -37.43 \| kcal/mol \| | \| 3' end stability \| : \| -6.58 \| kcal/mol \| \| --- \| --- \| --- \| --- \| \| ΔH \| : \| -177.5 \| kcal/mol \| \| ΔS \| : \| -0.47 \| kcal/°K/mol \| \| 5' end ΔG \| : \| -9.76 \| kcal/mol \| \| Self Dimer ( ΔG) \| : \| [-4.16](http://www.premierbiosoft.com/NetPrimer/www.premierbiosoft.com) \| kcal/mol \| \| Hairpin ( ΔG) \| : \| [-0.24](http://www.premierbiosoft.com/NetPrimer/www.premierbiosoft.com) \| kcal/mol \| \| Repeats (# of pairs) \| : \|  \| kcal/mol \| \| Run (# of bases) \| : \| [4](http://www.premierbiosoft.com/NetPrimer/www.premierbiosoft.com) \| kcal/mol \| |

| \| Cross Dimer (ΔG) \| : \| [-6.24](http://www.premierbiosoft.com/NetPrimer/www.premierbiosoft.com) \| kcal/mol \| \| --- \| --- \| --- \| --- \| |
| --- | --- | --- | --- | --- |
