# Supplementary material for: Investigation of pathogenic germline variants in gastric cancer and development of “GasCanBase” database
Source: Cancer Rep (Hoboken). 2023 Oct 22;6(12):e1906. doi: 10.1002/cnr2.1906 (PMC10728505; doi:10.1002/cnr2.1906)
Supplement: Supplementary file 1 — Data S1 Supporting Information. [file CNR2-6-e1906-s001.zip › Supplementary File/Table S6.2. Allele specific primer design on selected nsSNP of APC gene.docx]

[rs76306073](https://www.ncbi.nlm.nih.gov/projects/SNP/snp_ref.cgi?rs=76306073) *[Homo sapiens]*

TAGAGTCAGAGGAAGTTTTGCTTTT[G/T]ATTCACCTCATCATTACACGCCTAT

Chromosome: 5:112841115

Gene:APC

1. Allele specific primer design on wild type nucleotide of ABCB1 gene

|  | Forward Primer | Reverse Primer |
| --- | --- | --- |
| Sequence | CAGAGGAAGTTTTGCTTTTG | GCTGATTGTTGGTTGGAGGT |
| Length | 20 bp | 20 bp |
| Start | 530 | 748 |
| Tm | 55.3 °C | 60.0 °C |
| GC | 40.0 % | 50.0 % |
| Tm | 52.99 °C | 57.12 °C |
| GC% | 40.0 | 50.0 |
| Self-Dimer ( ΔG) | -4.54 kcal/mol | kcal/mol |
| Hairpin ( ΔG) | -0.14 kcal/mol | kcal/mol |
| Cross Dimer (ΔG) | kcal/mol | |
| Product size | 219 bp | |

2. Allele specific primer design on mutant nucleotide of ABCB1 gene

|  | Forward Primer | Reverse Primer |
| --- | --- | --- |
| Sequence | GTCAGAGGAAGTTTTGCTTTTT | GCTGATTGTTGGTTGGAGGT |
| Length | 22 bp | 20 bp |
| Start | 528 | 748 |
| Tm | 56.9 °C | 60.0 °C |
| GC | 36.4 % | 50.0 % |
| Tm | 55.47 °C | 57.12 °C |
| GC% | 36.36 | 50.0 |
| Self-Dimer ( ΔG) | -4.54 kcal/mol |  |
| Hairpin ( ΔG) | -0.14 kcal/mol |  |
| Cross Dimer (ΔG) | -5.13 kcal/mol | |
| Product size | 221 bp | |
