# Supplementary material for: Investigation of pathogenic germline variants in gastric cancer and development of “GasCanBase” database
Source: Cancer Rep (Hoboken). 2023 Oct 22;6(12):e1906. doi: 10.1002/cnr2.1906 (PMC10728505; doi:10.1002/cnr2.1906)
Supplement: Supplementary file 1 — Data S1 Supporting Information. [file CNR2-6-e1906-s001.zip › Supplementary File/Table S6.3. Allele specific primer design on selected nsSNP of BAX gene.docx]

[rs36017265](https://www.ncbi.nlm.nih.gov/projects/SNP/snp_ref.cgi?rs=36017265) *[Homo sapiens]*

CATCCAGGATCGAGCMGGGCGAATG[C/G/T]GGGGGGAGGCACCCGAGCTGGCCCT

Chromosome: 19:48955715

Gene:BAX

1. Allele specific primer design on wild type nucleotide of BAX gene

| Primer Criteria | Forward Primer | Reverse Primer |
| --- | --- | --- |
| Sequence | GGATCGAGCAGGGCGAATGG |  |
| Length | 20 bp | 20 bp |
| Start | 419 | 643 |
| Tm | 69.4 °C | 60.5 °C |
| GC | 65.0 % | 55.0 % |
| Tm | 67.14 °C | 58.3 °C |
| GC% | 65.0 | 55.0 |
| Self-Dimer ( ΔG) | -6.76 kcal/mol |  |
| Hairpin ( ΔG) | -1.09 kcal/mol |  |
| Cross Dimer (ΔG) | -6.09 kcal/mol | |
| Product size | 225 bp | |

1. Allele specific primer design on Mutant nucleotide of BAX gene

| Primer Criteria | Forward Primer | Reverse Primer |
| --- | --- | --- |
| Sequence | GGATCGAGCAGGGCGAATGC | TTAGGGGAGGAGGAGAATGC |
| Length | 20 bp | 20 bp |
| Start | 419 | 643 |
| Tm | 69.6 °C | 60.5 °C |
| GC | 65.0 % | 55.0 % |
| Tm | 67.28 °C | 58.3 °C |
| GC% | 65.0 | 55.0 |
| Self-Dimer ( ΔG) | -6.76 kcal/mol |  |
| Hairpin ( ΔG) | -1.99 kcal/mol |  |
| Cross Dimer (ΔG) | -6.09 kcal/mol | |
| Product size | 225 bp | |
