# Supplementary material for: Investigation of pathogenic germline variants in gastric cancer and development of “GasCanBase” database
Source: Cancer Rep (Hoboken). 2023 Oct 22;6(12):e1906. doi: 10.1002/cnr2.1906 (PMC10728505; doi:10.1002/cnr2.1906)
Supplement: Supplementary file 1 — Data S1 Supporting Information. [file CNR2-6-e1906-s001.zip › Supplementary File/Table S6.4. Allele specific primer design on selected nsSNP of BMPR1A gene.docx]

[rs35619497](https://www.ncbi.nlm.nih.gov/projects/SNP/snp_ref.cgi?rs=35619497) *[Homo sapiens]*

CCTAATCATTTGGGAGATGGCTCGT[C/T]GTTGTATCACAGGAGGTGGGAGTTT

Chromosome: 10:86921680

Gene:BMPR1A

1. Allele specific primer design on wild type nucleotide of BMPR1A gene

|  | Forward Primer | Reverse Primer |
| --- | --- | --- |
| Sequence | CATTTGGGAGATGGCTCGTC | ATGGCATGCCTGTATCAAAA |
| Length | 20 bp | 20 bp |
| Start | 498 | 686 |
| Tm | 62.9 °C | 59.0 °C |
| GC | 55.0 % | 40.0 % |
| Tm | 60.13 °C | 56.41 °C |
| GC% | 55.0 | 40.0 |
| Self-Dimer ( ΔG) | -4.17 kcal/mol |  |
| Hairpin ( ΔG) |  |  |
| Cross Dimer (ΔG) | -6.84 kcal/mol | |
| Product size | 189 bp | |

2. Allele specific primer design on mutant nucleotide of BMPR1A gene

|  | Forward Primer | Reverse Primer |
| --- | --- | --- |
| Sequence | CATTTGGGAGATGGCTCGTT | ATGGCATGCCTGTATCAAAA |
| Length | 20 bp | 20 bp |
| Start | 498 | 686 |
| Tm | 62.3 °C | 59.0 °C |
| GC | 50.0 % | 40.0 % |
| Tm | 59.84 °C | 56.41 °C |
| GC% | 50.0 | 40.0 |
| Self-Dimer ( ΔG) | -4.17 kcal/mol | -17.8 kcal/mol |
| Hairpin ( ΔG) |  |  |
| Cross Dimer (ΔG) | -6.84 kcal/mol | |
| Product size | 189 bp | |
