# Supplementary material for: Investigation of pathogenic germline variants in gastric cancer and development of “GasCanBase” database
Source: Cancer Rep (Hoboken). 2023 Oct 22;6(12):e1906. doi: 10.1002/cnr2.1906 (PMC10728505; doi:10.1002/cnr2.1906)
Supplement: Supplementary file 1 — Data S1 Supporting Information. [file CNR2-6-e1906-s001.zip › Supplementary File/Table S6.5. Allele specific primer design on selected nsSNP of CASP3 gene.docx]

[rs79348369](https://www.ncbi.nlm.nih.gov/projects/SNP/snp_ref.cgi?rs=79348369) *[Homo sapiens]*

ATGAAAAGTAGCGTCAAAGGAAAAG[G/T]ACTCAAATTCTGTTGCCACCTTTCG

Chromosome: 4:184629360

Gene:CASP3

1. Allele specific primer design on wild type nucleotide of CASP3 gene

|  | Forward Primer | Reverse Primer |
| --- | --- | --- |
| Sequence | AGTAGCGTCAAAGGAAAAGG | TTCTTGGCGAAATTCAAAGG |
| Length | 20 bp | 20 bp |
| Start | 535 | 688 |
| Tm | 56.3 °C | 60.2 °C |
| GC | 45.0 % | 40.0 % |
| Tm | 54.26 °C | 58.17 °C |
| GC% | 45.0 | 40.0 |
| Self-Dimer ( ΔG) |  | -5.36 kcal/mol |
| Hairpin ( ΔG) |  | -0.8 kcal/mol |
| Cross Dimer (ΔG) | -4.54 kcal/mol | |
| Product size | 154 bp | |

1. Allele specific primer design on mutant nucleotide of CASP3 gene

|  | Forward Primer | Reverse Primer |
| --- | --- | --- |
| Sequence | AGTAGCGTCAAAGGAAAAGT | TTCTTGGCGAAATTCAAAGG |
| Length | 20 bp | 20 bp |
| Start | 535 | 688 |
| Tm | 53.5 °C | 60.2 °C |
| GC | 40.0 % | 40.0 % |
| Tm | 51.32 °C | 58.17 °C |
| GC% | 40.0 | 40.0 |
| Self-Dimer ( ΔG) |  | -5.36 kcal/mol |
| Hairpin ( ΔG) |  | -0.8 kcal/mol |
| Cross Dimer (ΔG) | -4.54 kcal/mol | |
| Product size | 154 bp | |
