# Supplementary material for: Investigation of pathogenic germline variants in gastric cancer and development of “GasCanBase” database
Source: Cancer Rep (Hoboken). 2023 Oct 22;6(12):e1906. doi: 10.1002/cnr2.1906 (PMC10728505; doi:10.1002/cnr2.1906)
Supplement: Supplementary file 1 — Data S1 Supporting Information. [file CNR2-6-e1906-s001.zip › Supplementary File/Table S6.6. Allele specific primer design on selected nsSNP of CD44 gene.docx]

[rs61752932](https://www.ncbi.nlm.nih.gov/projects/SNP/snp_ref.cgi?rs=61752932) *[Homo sapiens]*

GCAGTTTGCATTGCAGTCAACAGTC[A/G]AAGAAGGTAAGGGGCTGTCCTGGGG

Chromosome: 11:35221726

Gene:CD44

1. Allele specific primer design on wild type nucleotide of CD44 gene

|  | Forward Primer | Reverse Primer |
| --- | --- | --- |
| Sequence | TGCATTGCAGTCAACAGTCG | GCACATGAATCCCTGGAAAC |
| Length | 20 bp | 20 bp |
| Start | 487 | 715 |
| Tm | 62.1 °C | 60.3 °C |
| GC | 50.0 % | 50.0 % |
| Tm | 58.46 °C | 57.44 °C |
| GC% | 50.0 | 50.0 |
| Self-Dimer ( ΔG) | -7.05 kcal/mol | -5.64 kcal/mol |
| Hairpin ( ΔG) |  |  |
| Cross Dimer (ΔG) |  | |
| Product size | 229 bp | |

2. Allele specific primer design on mutant nucleotide of CD44 gene

|  | Forward Primer | Reverse Primer |
| --- | --- | --- |
| Sequence | TGCATTGCAGTCAACAGTCA | GCACATGAATCCCTGGAAAC |
| Length | 20 bp | 20 bp |
| Start | 487 | 715 |
| Tm | 60.0 °C | 60.3 °C |
| GC | 45.0 % | 50.0 % |
| Tm | 56.01 °C | 57.44 °C |
| GC% | 45.0 | 50.0 |
| Self-Dimer ( ΔG) | -7.05 kcal/mol | -5.64 kcal/mol |
| Hairpin ( ΔG) |  |  |
| Cross Dimer (ΔG) | -4.53 kcal/mol | |
| Product size | 229 bp | |
