# Supplementary material for: Investigation of pathogenic germline variants in gastric cancer and development of “GasCanBase” database
Source: Cancer Rep (Hoboken). 2023 Oct 22;6(12):e1906. doi: 10.1002/cnr2.1906 (PMC10728505; doi:10.1002/cnr2.1906)
Supplement: Supplementary file 1 — Data S1 Supporting Information. [file CNR2-6-e1906-s001.zip › Supplementary File/Table S6.7. Allele specific primer design on selected nsSNP of CDH1 gene.docx]

[rs35572355](https://www.ncbi.nlm.nih.gov/projects/SNP/snp_ref.cgi?rs=35572355) *[Homo sapiens]*

AGCCCCGCCTTATGATTCTCTGCTC[A/C/G]TGTTTGACTATGAAGGAAGCGGTTC

Chromosome: 16:68833344

Gene:CDH1

1. Allele specific primer design on wild type nucleotide of CDH1 gene

|  | Forward Primer | Reverse Primer |
| --- | --- | --- |
| Sequence | GCCTTATGATTCTCTGCTCG | AACCACCAGCAACGTGATTT |
| Length | 20 bp | 20 bp |
| Start | 460 | 701 |
| Tm | 57.7 °C | 60.4 °C |
| GC | 50.0 % | 45.0 % |
| Tm | 55.14 °C | 57.77 °C |
| GC% | 50.0 | 45.0 |
| Self-Dimer ( ΔG) |  | -6.3 kcal/mol |
| Hairpin ( ΔG) |  |  |
| Cross Dimer (ΔG) | -6.69 kcal/mol | |
| Product size | 242 bp | |

2. Allele specific primer design on mutant nucleotide of CDH1 gene

|  | Forward Primer | Reverse Primer |
| --- | --- | --- |
| Sequence | GCCTTATGATTCTCTGCTCA | AACCACCAGCAACGTGATTT |
| Length | 20 bp | 20 bp |
| Start | 460 | 701 |
| Tm | 55.6 °C | 60.4 °C |
| GC | 45.0 % | 45.0 % |
| Tm | 52.76 °C | 57.77 °C |
| GC% | 45.0 | 45.0 |
| Self-Dimer ( ΔG) | -4.53 kcal/mol | -6.3 kcal/mol |
| Hairpin ( ΔG) | -0.43 kcal/mol |  |
| Cross Dimer (ΔG) | -6.69 kcal/mol | |
| Product size | 242 bp | |
