# Supplementary material for: Investigation of pathogenic germline variants in gastric cancer and development of “GasCanBase” database
Source: Cancer Rep (Hoboken). 2023 Oct 22;6(12):e1906. doi: 10.1002/cnr2.1906 (PMC10728505; doi:10.1002/cnr2.1906)
Supplement: Supplementary file 1 — Data S1 Supporting Information. [file CNR2-6-e1906-s001.zip › Supplementary File/Table S6.8. Allele specific primer design on selected nsSNP of CDKN1B gene.docx]

GTCTAACGGGAGCCCTAGCCTGGAG[C/T]GGATGGACGCCAGGCAGGCGGAGCA

Chromosome: 12:12717882

Gene:CDKN1B

1. Allele specific primer design on wild type nucleotide of CDKN1B gene

|  | Forward Primer | Reverse Primer |
| --- | --- | --- |
| Sequence | GGAGCCCTAGCCTGGAGC | TTCTCCACCTCTTGCCACTC |
| Length | 18 bp | 20 bp |
| Start | 499 | 715 |
| Tm | 62.4 °C | 60.4 °C |
| GC | 72.2 % | 55.0 % |
| Tm | 59.43 °C | 57.25 °C |
| GC% | 72.22 | 55.0 |
| Self-Dimer ( ΔG) | -4.16 kcal/mol |  |
| Hairpin ( ΔG) |  |  |
| Cross Dimer (ΔG) | -8.2 kcal/mol | |
| Product size | 217 bp | |

2. Allele specific primer design on mutant nucleotide of CDKN1B gene

|  | Forward Primer | Reverse Primer |
| --- | --- | --- |
| Sequence | GGAGCCCTAGCCTGGAGT | CTTCTCCACCTCTTGCCACT |
| Length | 18 bp | 20 bp |
| Start | 499 | 716 |
| Tm | 59.4 °C | 59.5 °C |
| GC | 66.7 % | 55.0 % |
| Tm | 56.25 °C | 56.55 °C |
| GC% | 66.67 | 55.0 |
| Self-Dimer ( ΔG) | -4.16 kcal/mol |  |
| Hairpin ( ΔG) |  |  |
| Cross Dimer (ΔG) | -8.2 kcal/mol | |
| Product size | 218 bp | |
