# Supplementary material for: Investigation of pathogenic germline variants in gastric cancer and development of “GasCanBase” database
Source: Cancer Rep (Hoboken). 2023 Oct 22;6(12):e1906. doi: 10.1002/cnr2.1906 (PMC10728505; doi:10.1002/cnr2.1906)

[rs3815780](https://www.ncbi.nlm.nih.gov/projects/SNP/snp_ref.cgi?rs=3815780) *[Homo sapiens]*

CTTGTGAATGAAGAAGCAACTGGCC[A/C]GTTCCGGGTATACCGTGAGTGATTC

Chromosome: 19:41710025

Gene: CEACAM5

1. Allele specific primer design on wild type nucleotide of CEACAM5 gene

|  | Forward Primer | Reverse Primer |
| --- | --- | --- |
| Sequence | AATGAAGAAGCAACTGGCCA | ATGCCGGAAAGGAATTCTG |
| Length | 20 bp | 19 bp |
| Start | 419 | 637 |
| Tm | 60.8 °C | 60.0 °C |
| GC | 45.0 % | 47.4 % |
| Tm | 58.17 °C | 55.49 °C |
| GC% | 45.0 | 60.0 |
| Self-Dimer ( ΔG) | -14.19 kcal/mol | -10.24 kcal/mol |
| Hairpin ( ΔG) |  |  |
| Cross Dimer (ΔG) | -4.17 kcal/mol | |
| Product size | 219 bp | |

2. Allele specific primer design on mutant nucleotide of CEACAM5 gene

|  | Forward Primer | Reverse Primer |
| --- | --- | --- |
| Sequence | AATGAAGAAGCAACTGGCCC | ATGCCGGAAAGGAATTCTG |
| Length | 20 bp | 19 bp |
| Start | 419 | 637 |
| Tm | 61.5 °C | 60.0 °C |
| GC | 50.0 % | 47.4 % |
| Tm | 59.24 °C | 57.48 °C |
| GC% | 50.0 | 47.37 |
| Self-Dimer ( ΔG) | -10.28 kcal/mol | -9.75 kcal/mol |
| Hairpin ( ΔG) |  | -0.12 kcal/mol |
| Cross Dimer (ΔG) | -6.12 kcal/mol | |
| Product size | 219 bp | |


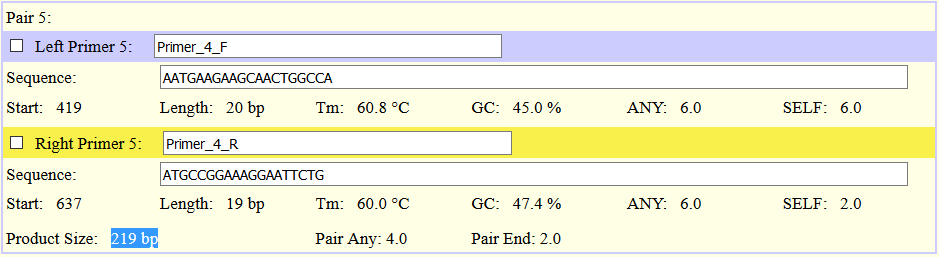


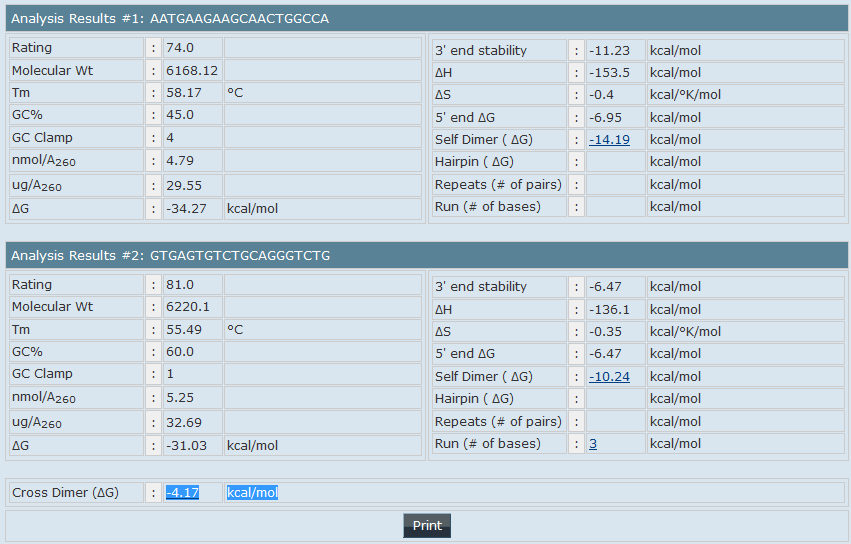


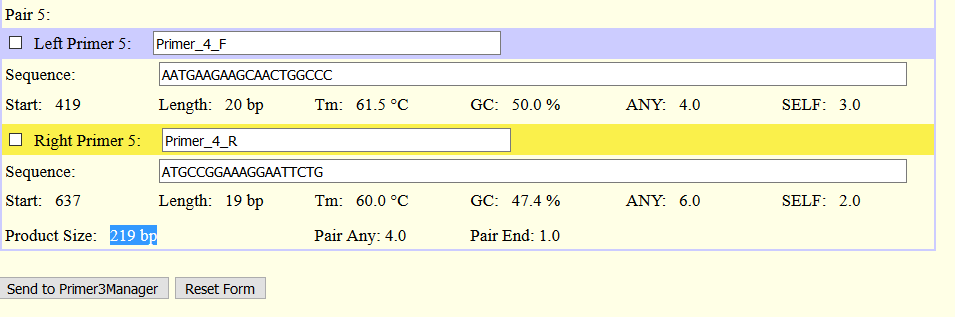


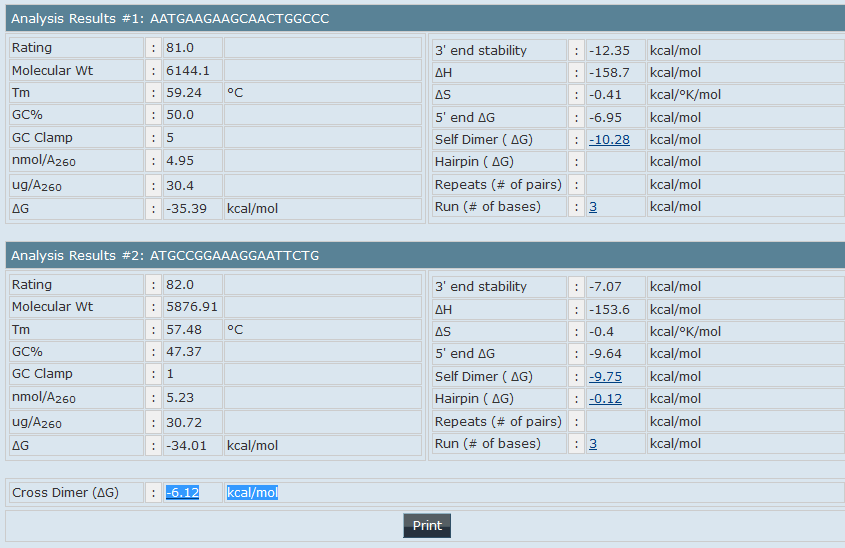

Supplement: Supplementary file 1 — Data S1 Supporting Information. [file CNR2-6-e1906-s001.zip › Supplementary File/Table S6.9. Allele specific primer design on selected nsSNP of CEACAM5 gene.docx]
