# Supplementary material for: Investigation of pathogenic germline variants in gastric cancer and development of “GasCanBase” database
Source: Cancer Rep (Hoboken). 2023 Oct 22;6(12):e1906. doi: 10.1002/cnr2.1906 (PMC10728505; doi:10.1002/cnr2.1906)
Supplement: Supplementary file 1 — Data S1 Supporting Information. [file CNR2-6-e1906-s001.zip › Supplementary File/Table S60. Prediction of damaging effect on EPCAM.docx]

Table S60. Prediction of damaging effect on EPCAM

| **SNP** | **Protein ID** | **Amino acid** | **Amino acid change** | **SIFT** | **PolyPhen2** | **PMut** | **MutPred** | **SNAP2** | **SNP&GO** | **PANTHER** |
| --- | --- | --- | --- | --- | --- | --- | --- | --- | --- | --- |
| rs74531854 | NP_002345 | 314 | T172M | Damaging | Probably Damaging | Neutral | 0.209 | Neutral | Neutral | Probably Benign |
| rs115283528 | NP_002345 | 314 | I277M | Damaging | Possibly Damaging | Neutral | 0.341 | Effect 91% | Neutral | Probably Benign |
| rs11554293 | NP_002345 | 314 | A64P | Damaging | Benign | Neutral | 0.355 | Neutral | Neutral | Probably Benign |
