# Supplementary material for: Investigation of pathogenic germline variants in gastric cancer and development of “GasCanBase” database
Source: Cancer Rep (Hoboken). 2023 Oct 22;6(12):e1906. doi: 10.1002/cnr2.1906 (PMC10728505; doi:10.1002/cnr2.1906)
Supplement: Supplementary file 1 — Data S1 Supporting Information. [file CNR2-6-e1906-s001.zip › Supplementary File/Table S62. Prediction of damaging effect on KIT.docx]

Table S62. Prediction of damaging effect on KIT

| **SNP** | **Protein ID** | **Amino acid** | **Amino acid change** | **SIFT** | **PolyPhen2** | **PMut** | **MutPred** | **SNAP2** | **SNP&GO** | **PANTHER** |
| --- | --- | --- | --- | --- | --- | --- | --- | --- | --- | --- |
| rs121913506 | NP_000213 | 976 | D816H | Damaging | Possibly Damaging | 0.6622 Pathological | 0.502 | Effect 95% | Disease | Cannot Score Substitution |
| rs28933371 | NP_000213 | 976 | F584C | Damaging | Probably Damaging | 0.7629 Pathological | 0.807 | Effect 71% | Disease | Cannot Score Substitution |
| rs55792975 | NP_000213 | 976 | V532I | Damaging | Benign | Neutral | 0.193 | Neutral | Neutral | Cannot Score Substitution |
| rs121913507 | NP_000213 | 976 | D816V | Damaging | Probably Damaging | 0.5079 Pathological | 0.897 | Effect 95% | Disease | Cannot Score Substitution |
| rs121913509 | NP_000213 | 976 | E839K | Damaging | Probably Damaging | 0.5685 Pathological | 0.963 | Effect 91% | Disease | Cannot Score Substitution |
| rs121913512 | NP_000213 | 976 | K642E | Damaging | Probably Damaging | Neutral | 0.839 | Effect 75% | Disease | Cannot Score Substitution |
| rs121913517 | NP_000213 | 976 | V559A | Damaging | Probably Damaging | Neutral | 0.739 | Effect 71% | Neutral | Cannot Score Substitution |
| rs35200131 | NP_000213 | 976 | C691S | Damaging | Possibly Damaging | Neutral | 0.208 | Effect 59% | Neutral | Cannot Score Substitution |
| rs56225530 | NP_000213 | 976 | T488M | Damaging | Probably Damaging | 0.6515 Pathological | 0.364 | Effect 66% | Neutral | Cannot Score Substitution |
| rs111466688 | NP_000213 | 976 | M618T | Damaging | Possibly Damaging | 0.8380 Pathological | 0.631 | Neutral | Neutral | Cannot Score Substitution |
| rs121913235 | NP_000213 | 976 | W557R | Damaging | Probably Damaging | 0.9505 Pathological | 0.753 | Effect 85% | Disease | Cannot Score Substitution |
| rs121913513 | NP_000213 | 976 | L576P | Damaging | Possibly Damaging | 0.8460 Pathological | 0.680 | Neutral | Disease | Cannot Score Substitution |
| rs121913514 | NP_000213 | 976 | N822K | Damaging | Probably Damaging | 0.7051 Pathological | 0.607 | Effect 71% | Disease | Cannot Score Substitution |
| rs121913516 | NP_000213 | 976 | T670I | Damaging | Probably Damaging | 0.6598 Pathological | 0.716 | Effect 71% | Disease | Cannot Score Substitution |
| rs121913520 | NP_000213 | 976 | V559I | Damaging | Possibly Damaging | Neutral | 0.523 | Neutral | Neutral | Cannot Score Substitution |
| rs121913521 | NP_000213 | 976 | V560D | Damaging | Probably Damaging | Neutral | 0.697 | Effect 80% | Disease | Cannot Score Substitution |
| rs121913523 | NP_000213 | 976 | V654A | Damaging | Benign | Neutral | 0.793 | Effect 75% | Disease | Cannot Score Substitution |
| rs121913524 | NP_000213 | 976 | V825A | Damaging | Probably Damaging | Neutral | 0.519 | Effect 66% | Neutral | Cannot Score Substitution |
