# Supplementary material for: Investigation of pathogenic germline variants in gastric cancer and development of “GasCanBase” database
Source: Cancer Rep (Hoboken). 2023 Oct 22;6(12):e1906. doi: 10.1002/cnr2.1906 (PMC10728505; doi:10.1002/cnr2.1906)
Supplement: Supplementary file 1 — Data S1 Supporting Information. [file CNR2-6-e1906-s001.zip › Supplementary File/Table S63. Prediction of damaging effect on KITLG.docx]

Table S63. Prediction of damaging effect on KITLG

| **SNP** | **Protein ID** | **Amino acid** | **Amino acid change** | **SIFT** | **PolyPhen2** | **PMut** | **MutPred** | **SNAP2** | **SNP&GO** | **PANTHER** |
| --- | --- | --- | --- | --- | --- | --- | --- | --- | --- | --- |
| rs121918653 | NP_000890 | 273 | N36S | Damaging | Benign | Neutral | 0.914 | Neutral | Neutral | Probably Benign |
| rs12721563 | NP_000890 | 273 | F232C | Damaging | Probably Damaging | 0.8899 Pathological | 0.642 | Effect 59% | Disease | Probably Benign |
| rs12721563 | NP_000890 | 273 | F232Y | Damaging | Probably Damaging | Neutral | 0.246 | Effect 75% | Neutral | Probably Benign |
| rs41283112 | NP_000890 | 273 | D210Y | Damaging | Probably Damaging | 0.8416 Pathological | 0.758 | Effect 85% | Neutral | Probably Benign |
| rs41416044 | NP_000890 | 273 | D149Y | Damaging | Probably Damaging | 0.9356 Pathological | 0.624 | Effect 80% | Disease | Probably Benign |
