# Supplementary material for: Investigation of pathogenic germline variants in gastric cancer and development of “GasCanBase” database
Source: Cancer Rep (Hoboken). 2023 Oct 22;6(12):e1906. doi: 10.1002/cnr2.1906 (PMC10728505; doi:10.1002/cnr2.1906)
Supplement: Supplementary file 1 — Data S1 Supporting Information. [file CNR2-6-e1906-s001.zip › Supplementary File/Table S64. Prediction of damaging effect on KRAS.docx]

Table S64. Prediction of damaging effect on KRAS

| **SNP** | **Protein ID** | **Amino acid** | **Amino acid change** | **SIFT** | **PolyPhen2** | **PMut** | **MutPred** | **SNAP2** | **SNP&GO** | **PANTHER** |
| --- | --- | --- | --- | --- | --- | --- | --- | --- | --- | --- |
| rs17851045 | NP_004976.2 | 188 | Q61H | Damaging | Benign | 0.7304 Pathological | 0.737 | Effect 75% | Neutral | Cannot Score Substitution |
| rs104886028 | NP_004976.2 | 188 | M72I | Damaging | Probably Damaging | Neutral | 0.635 | Effect 59% | Neutral | Cannot Score Substitution |
| rs104886029 | NP_004976.2 | 188 | A59V | Damaging | Probably Damaging | 0.9322 Pathological | 0.789 | Effect 66% | Disease | Cannot Score Substitution |
| rs104894359 | NP_004976.2 | 188 | G60R | Damaging | Probably Damaging | 0.8525 Pathological | 0.972 | Effect 95% | Disease | Cannot Score Substitution |
| rs104894360 | NP_004976.2 | 188 | D153V | Damaging | Possibly Damaging | 0.8822 Pathological | 0.817 | Effect 63% | Neutral | Cannot Score Substitution |
| rs104894361 | NP_004976.2 | 188 | K5N | Damaging | Probably Damaging | 0.7177 Pathological | 0.933 | Effect 75% | Neutral | Cannot Score Substitution |
| rs104894362 | NP_004976.2 | 188 | F156L | Damaging | Probably Damaging | 0.6458 Pathological | 0.956 | Effect 75% | Disease | Cannot Score Substitution |
| rs104894364 | NP_004976.2 | 188 | T58I | Damaging | Probably Damaging | 0.8935 Pathological | 0.967 | Effect 85% | Disease | Cannot Score Substitution |
| rs104894365 | NP_004976.2 | 188 | V14I | Damaging | Probably Damaging | Neutral | 0.922 | Effect 85% | Neutral | Cannot Score Substitution |
| rs104894366 | NP_004976.2 | 188 | P34R | Damaging | Probably Damaging | 0.9585 Pathological | 0.935 | Effect 91% | Disease | Cannot Score Substitution |
| rs104894367 | NP_004976.2 | 188 | V152G | Damaging | Probably Damaging | 0.9117 Pathological | 0.956 | Effect 85% | Neutral | Cannot Score Substitution |
| rs112445441 | NP_004976.2 | 188 | G13D | Damaging | Benign | 0.9664 Pathological | 0.926 | Effect 91% | Disease | Cannot Score Substitution |
| rs121913238 | NP_004976.2 | 188 | Q61E | Damaging | Possibly Damaging | Neutral | 0.703 | Effect 75% | Neutral | Cannot Score Substitution |
| rs121913240 | NP_004976.2 | 188 | Q61L | Damaging | Probably Damaging | 0.8557 Pathological | 0.716 | Effect 75% | Neutral | Cannot Score Substitution |
| rs121913527 | NP_004976.2 | 188 | A146T | Damaging | Probably Damaging | 0.7629 Pathological | 0.913 | Effect 80% | Disease | Cannot Score Substitution |
| rs121913528 | NP_004976.2 | 188 | A59T | Damaging | Possibly Damaging | 0.8276 Pathological | 0.778 | Effect 71% | Disease | Cannot Score Substitution |
| rs121913529 | NP_004976.2 | 188 | G12A | Damaging | Possibly Damaging | 0.9024 Pathological | 0.891 | Effect 85% | Neutral | Cannot Score Substitution |
| rs121913530 | NP_004976.2 | 188 | G12C | Damaging | Probably Damaging | 0.9619 Pathological | 0.894 | Effect 75% | Disease | Cannot Score Substitution |
| rs121913535 | NP_004976.2 | 188 | G13C | Damaging | Probably Damaging | 0.9678 Pathological | 0.927 | Effect 75% | Disease | Cannot Score Substitution |
| rs121913538 | NP_004976.2 | 188 | L19F | Damaging | Probably Damaging | 0.7742 Pathological | 0.739 | Effect 75% | Disease | Cannot Score Substitution |
| rs121913236 | NP_004976.2 | 188 | Q22K | Damaging | Probably Damaging | 0.7697 Pathological | 0.909 | Effect 75% | Disease | Cannot Score Substitution |
