# Supplementary material for: Investigation of pathogenic germline variants in gastric cancer and development of “GasCanBase” database
Source: Cancer Rep (Hoboken). 2023 Oct 22;6(12):e1906. doi: 10.1002/cnr2.1906 (PMC10728505; doi:10.1002/cnr2.1906)
Supplement: Supplementary file 1 — Data S1 Supporting Information. [file CNR2-6-e1906-s001.zip › Supplementary File/Table S65. Prediction of damaging effect on KRT20.docx]

Table S65. Prediction of damaging effect on KRT20

| **SNP** | **Protein ID** | **Amino acid** | **Amino acid change** | **SIFT** | **PolyPhen2** | **PMut** | **MutPred** | **SNAP2** | **SNP&GO** | **PANTHER** |
| --- | --- | --- | --- | --- | --- | --- | --- | --- | --- | --- |
| rs111500551 | NP_061883 | 424 | H208Y | Damaging | Benign | Neutral | 0.839 | Effect 63% | Disease | Possibly Damaging |
| rs113823624 | NP_061883 | 424 | V413M | Damaging | Probably Damaging | Neutral | 0.402 | Effect 63% | Neutral | Possibly Damaging |
