# Supplementary material for: Investigation of pathogenic germline variants in gastric cancer and development of “GasCanBase” database
Source: Cancer Rep (Hoboken). 2023 Oct 22;6(12):e1906. doi: 10.1002/cnr2.1906 (PMC10728505; doi:10.1002/cnr2.1906)
Supplement: Supplementary file 1 — Data S1 Supporting Information. [file CNR2-6-e1906-s001.zip › Supplementary File/Table S67. Prediction of damaging effect on MET.docx]

Table S67. Prediction of damaging effect on MET

| **SNP** | **Protein ID** | **Amino acid** | **Amino acid change** | **SIFT** | **PolyPhen2** | **PMut** | **MutPred** | **SNAP2** | **SNP&GO** | **PANTHER** |
| --- | --- | --- | --- | --- | --- | --- | --- | --- | --- | --- |
| rs80256822 | NP_000236 | 1390 | A48V | Damaging | Possibly Damaging | Neutral | 0.439 | Neutral | Disease | Possibly Damaging |
| rs35776110 | NP_000236 | 1390 | A320V | Damaging | Probably Damaging | Neutral | 0.314 | Effect 66% | Disease | Probably Damaging |
| rs34589476 | NP_000236 | 1390 | R970C | Damaging | Probably Damaging | 0.8790 Pathological | 0.219 | Effect 80% | Disease | Possibly Damaging |
| rs121913243 | NP_000236 | 1390 | H1094R | Damaging | Probably Damaging | 0.5145 Pathological | 0.783 | Effect 80% | Disease | Probably Damaging |
| rs121913244 | NP_000236 | 1390 | H1094Y | Damaging | Probably Damaging | Neutral | 0.831 | Effect 80% | Disease | Probably Damaging |
| rs45541232 | NP_000236 | 1390 | D1180N | Damaging | Probably Damaging | Neutral | 0.709 | Effect 75% | Disease | Probably Damaging |
| rs121913246 | NP_000236 | 1390 | Y1230C | Damaging | Probably Damaging | 0.8789 Pathological | 0.678 | Effect 59% | Disease | Probably Damaging |
| rs121913247 | NP_000236 | 1390 | Y1230H | Damaging | Probably Damaging | Neutral | 0.692 | Effect 59% | Disease | Probably Damaging |
| rs45454696 | NP_000236 | 1390 | S1254N | Damaging | Probably Damaging | Neutral | 0.841 | Effect 59% | Disease | Probably Damaging |
| rs45532942 | NP_000236 | 1390 | D1265Y | Damaging | Probably Damaging | 0.7823 Pathological | 0.899 | Effect 95% | Disease | Probably Damaging |
| rs33917957 | NP_000236 | 1390 | N375S | Damaging | Benign | Neutral | 0.234 | Effect 59% | Neutral | Probably Damaging |
| rs34349517 | NP_000236 | 1390 | L238S | Damaging | Probably Damaging | Neutral | 0.258 | Effect 63% | Neutral | Probably Damaging |
| rs45607832 | NP_000236 | 1390 | R970H | Damaging | Probably Damaging | Neutral | 0.342 | Effect 53% | Neutral | Possibly Damaging |
| rs55985569 | NP_000236 | 1390 | E168D | Damaging | Possibly Damaging | Neutral | 0.326 | Effect 80% | Neutral | Possibly Damaging |
| rs56391007 | NP_000236 | 1390 | T992I | Damaging | Probably Damaging | 0.5577 Pathological | 0.123 | Effect 91% | Neutral | Probably Damaging |
| rs77523018 | NP_000236 | 1390 | M362T | Damaging | Benign | Neutral | 0.699 | Effect 80% | Neutral | Probably Benign |
| rs35225896 | NP_000236 | 1390 | I316M | Damaging | Possibly Damaging | Neutral | 0.255 | Effect 80% | Neutral | Possibly Damaging |
| rs45483396 | NP_000236 | 1390 | L211W | Damaging | Probably Damaging | Neutral | 0.537 | Effect 59% | Neutral | Possibly Damaging |
| rs45553236 | NP_000236 | 1390 | R739H | Damaging | Possibly Damaging | 0.5471 Pathological | 0.438 | Neutral | Neutral | Probably Benign |
| rs45578433 | NP_000236 | 1390 | A1363T | Damaging | Possibly Damaging | Neutral | 0.359 | Neutral | Neutral | Possibly Damaging |
| rs45585831 | NP_000236 | 1390 | T495I | Damaging | Benign | Neutral | 0.586 | Neutral | Neutral | Probably Benign |
| rs45586239 | NP_000236 | 1390 | H633L | Damaging | Benign | 0.8905 Pathological | 0.569 | Neutral | Neutral | Possibly Damaging |
| rs45587940 | NP_000236 | 1390 | R739C | Damaging | Probably Damaging | 0.7844 Pathological | 0.558 | Effect 63% | Neutral | Probably Benign |
| rs45602940 | NP_000236 | 1390 | R591W | Damaging | Probably Damaging | 0.9628 Pathological | 0.732 | Effect 75% | Neutral | Possibly Damaging |
| rs45612435 | NP_000236 | 1390 | R1022Q | Damaging | Probably Damaging | 0.5376 Pathological | 0.454 | Effect 59% | Neutral | Probably Damaging |
| rs45628136 | NP_000236 | 1390 | Q1067K | Damaging | Benign | Neutral | 0.656 | Neutral | Neutral | Possibly Damaging |
| rs56311081 | NP_000236 | 1390 | S156L | Damaging | Possibly Damaging | Neutral | 0.407 | Neutral | Neutral | Probably Damaging |
| rs1801762 | NP_003790 | 476 | K93E | Damaging | Possibly Damaging | Neutral | 0.177 | Neutral | Neutral | Probably Benign |
| rs35469582 | NP_000236 | 1390 | R143Q | Damaging | Probably Damaging | 0.5106 Pathological | 0.367 | Neutral | Neutral | Probably Damaging |
| rs35601148 | NP_000236 | 1390 | T309P | Damaging | Benign | Neutral | 0.468 | Neutral | Neutral | Probably Damaging |
| rs45440991 | NP_000236 | 1390 | R793C | Damaging | Probably Damaging | 0.7653 Pathological | 0.634 | Effect 66% | Neutral | Probably Damaging |
| rs45441497 | NP_000236 | 1390 | L402I | Damaging | Possibly Damaging | Neutral | 0.487 | Neutral | Neutral | Probably Benign |
| rs45446492 | NP_000236 | 1390 | R731Q | Damaging | Probably Damaging | 0.5187 Pathological | 0.511 | Effect 91% | Neutral | Probably Damaging |
| rs45450897 | NP_000236 | 1390 | I834M | Damaging | Benign | Neutral | 0.560 | Neutral | Neutral | Possibly Damaging |
| rs45460604 | NP_000236 | 1390 | Q559R | Damaging | Benign | Neutral | 0.664 | Neutral | Neutral | Probably Benign |
| rs45471794 | NP_000236 | 1390 | R982M | Damaging | Probably Damaging | 0.8849 Pathological | 0.722 | Neutral | Neutral | Probably Damaging |
| rs45531032 | NP_000236 | 1390 | I747V | Damaging | Benign | Neutral | 0.546 | Neutral | Neutral | Probably Damaging |
| rs45541232 | NP_000236 | 1390 | D1180N | Damaging | Probably Damaging | Neutral | 0.709 | Effect 75% | Neutral | Probably Damaging |
| rs45551737 | NP_000236 | 1390 | P239R | Damaging | Probably Damaging | Neutral | 0.880 | Effect 85% | Neutral | Probably Damaging |
| rs45561544 | NP_000236 | 1390 | D981E | Damaging | Probably Damaging | Neutral | 0.276 | Neutral | Neutral | Probably Damaging |
| rs45564937 | NP_000236 | 1390 | M1031V | Damaging | Benign | Neutral | 0.170 | Neutral | Neutral | Probably Benign |
| rs45571834 | NP_000236 | 1390 | I1053T | Damaging | Probably Damaging | 0.5446 Pathological | 0.587 | Effect 71% | Neutral | Probably Damaging |
| rs45575240 | NP_000236 | 1390 | G853C | Damaging | Probably Damaging | 0.7813 Pathological | 0.574 | Effect 53% | Neutral | Possibly Damaging |
| rs45583838 | NP_000236 | 1390 | L1097V | Damaging | Probably Damaging | Neutral | 0.899 | Effect 53% | Neutral | Probably Damaging |
| rs45592846 | NP_000236 | 1390 | T1096S | Damaging | Probably Damaging | Neutral | 0.627 | Neutral | Neutral | Probably Damaging |
| rs45595632 | NP_000236 | 1390 | T1261A | Damaging | Probably Damaging | Neutral | 0.726 | Effect 75% | Neutral | Probably Damaging |
| rs45604032 | NP_000236 | 1390 | G933R | Damaging | Possibly Damaging | 0.7236 Pathological | 0.741 | Effect 80% | Neutral | Probably Damaging |
| rs45605635 | NP_000236 | 1390 | Y649C | Damaging | Probably Damaging | 0.7959 Pathological | 0.512 | Effect 63% | Neutral | Probably Benign |
| rs56361366 | NP_000236 | 1390 | S661R | Damaging | Possibly Damaging | 0.6078 Pathological | 0.659 | Effect 75% | Neutral | Probably Benign |
| rs61730997 | NP_003790 | 476 | A20P | Damaging | Benign | Neutral | 0.057 | Effect 71% | Neutral | Possibly Damaging |
| rs77651398 | NP_000236 | 1390 | N45S | Damaging | Probably Damaging | Neutral | 0.296 | Neutral | Neutral | Possibly Damaging |
| rs79252026 | NP_003790 | 476 | V368G | Damaging | Probably Damaging | 0.8050 Pathological | 0.762 | Effect 80% | Neutral | Probably Damaging |
| rs112590484 | NP_003790 | 476 | S71P | Damaging | Possibly Damaging | 0.6639 Pathological | 0.101 | Neutral | Neutral | Probably Benign |
| rs115527371 | NP_003790 | 476 | Q193E | Damaging | Possibly Damaging | Neutral | 0.594 | Neutral | Neutral | Probably Damaging |
| rs115821778 | NP_003790 | 476 | V22M | Damaging | Benign | Neutral | 0.072 | Neutral | Neutral | Probably Benign |
| rs116111155 | NP_003790 | 476 | R247C | Damaging | Possibly Damaging | 0.8770 Pathological | 0.599 | Effect 66% | Neutral | Possibly Damaging |
| rs116730904 | NP_003790 | 476 | A297V | Damaging | Probably Damaging | 0.6440 Pathological | 0.841 | Effect 59% | Neutral | Probably Damaging |
| rs121913245 | NP_000236 | 1390 | M1250T | Damaging | Probably Damaging | 0.8460 Pathological | 0.837 | Effect 75% | Neutral | Probably Damaging |
