# Supplementary material for: Investigation of pathogenic germline variants in gastric cancer and development of “GasCanBase” database
Source: Cancer Rep (Hoboken). 2023 Oct 22;6(12):e1906. doi: 10.1002/cnr2.1906 (PMC10728505; doi:10.1002/cnr2.1906)
Supplement: Supplementary file 1 — Data S1 Supporting Information. [file CNR2-6-e1906-s001.zip › Supplementary File/Table S68. Prediction of damaging effect on MGMT.docx]

Table S68. Prediction of damaging effect on MGMT

| **SNP** | **Protein ID** | **Amino acid** | **Amino acid change** | **SIFT** | **PolyPhen2** | **PMut** | **MutPred** | **SNAP2** | **SNP&GO** | **PANTHER** |
| --- | --- | --- | --- | --- | --- | --- | --- | --- | --- | --- |
| rs2282164 | NP_002403 | 238 | W96C | Damaging | Probably Damaging | Neutral | 0.596 | Effect 75% | Disease | Probably Damaging |
| rs3750824 | NP_002403 | 238 | R159Q | Damaging | Probably Damaging | 0.6881 Pathological | 0.892 | Effect 91% | Disease | Probably Damaging |
| rs2308318 | NP_002403 | 238 | G191R | Damaging | Probably Damaging | 0.5824 Pathological | 0.788 | Effect 91% | Disease | Probably Damaging |
| rs2308320 | NP_002403 | 238 | E197D | Damaging | Benign | Neutral | 0.575 | Neutral | Neutral | Possibly Damaging |
| rs2308327 | NP_002403 | 238 | K209R | Damaging | Benign | Neutral | 0.266 | Neutral | Neutral | Probably Benign |
| rs2308322 | NP_002403 | 238 | P89S | Damaging | Probably Damaging | Neutral | 0.447 | Neutral | Neutral | Probably Damaging |
