# Supplementary material for: Investigation of pathogenic germline variants in gastric cancer and development of “GasCanBase” database
Source: Cancer Rep (Hoboken). 2023 Oct 22;6(12):e1906. doi: 10.1002/cnr2.1906 (PMC10728505; doi:10.1002/cnr2.1906)
Supplement: Supplementary file 1 — Data S1 Supporting Information. [file CNR2-6-e1906-s001.zip › Supplementary File/Table S69. Prediction of damaging effect on MMP2.docx]

Table S69. Prediction of damaging effect on MMP2

| **SNP** | **Protein ID** | **Amino acid** | **Amino acid change** | **SIFT** | **PolyPhen2** | **PMut** | **MutPred** | **SNAP2** | **SNP&GO** | **PANTHER** |
| --- | --- | --- | --- | --- | --- | --- | --- | --- | --- | --- |
| rs112710941 | NP_001121363 | 610 | R65H | Damaging | Benign | 0.5433 Pathological | 0.492 | Neutral | Disease | Probably Damaging |
| rs11542001 | NP_001121363 | 610 | F189L | Damaging | Possibly Damaging | Neutral | 0.581 | Effect 66% | Disease | Probably Damaging |
| rs111609606 | NP_001121363 | 610 | C282F | Damaging | Probably Damaging | 0.7765 Pathological | 0.579 | Effect 91% | Disease | Probably Damaging |
| rs121908741 | NP_001121363 | 610 | G356D | Damaging | Probably Damaging | 0.6269 Pathological | 0.811 | Effect 80% | Disease | Probably Damaging |
| rs28730814 | NP_001121363 | 610 | R450H | Damaging | Possibly Damaging | 0.6661 Pathological | 0.290 | Neutral | Disease | Probably Damaging |
| rs59727333 | NP_001121363 | 610 | M359I | Damaging | Benign | Neutral | 0.711 | Neutral | Neutral | Possibly Damaging |
