# Supplementary material for: Investigation of pathogenic germline variants in gastric cancer and development of “GasCanBase” database
Source: Cancer Rep (Hoboken). 2023 Oct 22;6(12):e1906. doi: 10.1002/cnr2.1906 (PMC10728505; doi:10.1002/cnr2.1906)
Supplement: Supplementary file 1 — Data S1 Supporting Information. [file CNR2-6-e1906-s001.zip › Supplementary File/Table S70. Prediction of damaging effect on MSH2.docx]

Table S70. Prediction of damaging effect on MSH2

| **SNP** | **Protein ID** | **Amino acid** | **Amino acid change** | **SIFT** | **PolyPhen2** | **PMut** | **MutPred** | **SNAP2** | **SNP&GO** | **PANTHER** |
| --- | --- | --- | --- | --- | --- | --- | --- | --- | --- | --- |
| rs4987188 | NP_000242 | 934 | G322D | Damaging | Benign | Neutral | 0.202 | Effect 85% | Neutral | Possibly Damaging |
| rs17217723 | NP_000242 | 934 | Y43C | Damaging | Probably Damaging | Neutral | 0.378 | Effect 85% | Disease | Probably Damaging |
| rs17217772 | NP_000242 | 934 | N127S | Damaging | Possibly Damaging | Neutral | 0.699 | Effect 91% | Neutral | Probably Damaging |
| rs17224367 | NP_000242 | 934 | L390F | Damaging | Benign | Neutral | 0.836 | Effect 91% | Neutral | Probably Damaging |
| rs33946261 | NP_000242 | 934 | H46Q | Damaging | Probably Damaging | Neutral | 0.932 | Effect 80% | Neutral | Probably Damaging |
| rs34136999 | NP_000242 | 934 | A272V | Damaging | Possibly Damaging | 0.6333 Pathological | 0.876 | Neutral | Neutral | Probably Damaging |
| rs34319539 | NP_000242 | 934 | K909I | Damaging | Benign | 0.7119 Pathological | 0.507 | Neutral | Neutral | Possibly Damaging |
| rs41294982 | NP_000242 | 934 | P670L | Damaging | Probably Damaging | Neutral | 0.393 | Effect 85% | Disease | Probably Damaging |
| rs41295182 | NP_000242 | 934 | L911R | Damaging | Probably Damaging | 0.6626 Pathological | 0.777 | Effect 66% | Disease | Probably Damaging |
| rs41295290 | NP_000242 | 934 | D646G | Damaging | Possibly Damaging | 0.6714 Pathological | 0.591 | Effect 66% | Neutral | Probably Damaging |
| rs1800152 | NP_000242 | 934 | H639Q | Damaging | Probably Damaging | Neutral | 0.977 | Effect 91% | Disease | Probably Damaging |
| rs2229061 | NP_000242 | 934 | I735V | Damaging | Probably Damaging | Neutral | 0.760 | Neutral | Neutral | Probably Damaging |
| rs28929483 | NP_000242 | 934 | P622L | Damaging | Probably Damaging | Neutral | 0.976 | Effect 95% | Disease | Probably Damaging |
| rs41295292 | NP_000242 | 934 | M779I | Damaging | Benign | Neutral | 0.283 | Neutral | Neutral | Possibly Damaging |
| rs56170584 | NP_000242 | 934 | P5Q | Damaging | Possibly Damaging | Neutral | 0.676 | Neutral | Neutral | Probably Damaging |
| rs63749946 | NP_000242 | 934 | C641G | Damaging | Probably Damaging | Neutral | 0.415 | Effect 80% | Disease | Probably Damaging |
| rs63749982 | NP_000242 | 934 | Y619C | Damaging | Probably Damaging | 0.5898 Pathological | 0.951 | Effect 80% | Neutral | Probably Damaging |
| rs63749993 | NP_000242 | 934 | M688R | Damaging | Probably Damaging | 0.6796 Pathological | 0.950 | Effect 85% | Disease | Probably Damaging |
| rs63750002 | NP_000242 | 934 | R96H | Damaging | Probably Damaging | 0.6823 Pathological | 0.356 | Neutral | Neutral | Probably Damaging |
| rs63750029 | NP_000242 | 934 | R534C | Damaging | Probably Damaging | 0.9153 Pathological | 0.572 | Effect 59% | Neutral | Probably Damaging |
| rs63750058 | NP_000242 | 934 | S269L | Damaging | Benign | Neutral | 0.758 | Effect 80% | Neutral | Possibly Damaging |
| rs63750070 | NP_000242 | 934 | L173P | Damaging | Probably Damaging | Neutral | 0.963 | Effect 85% | Disease | Probably Damaging |
| rs63750070 | NP_000242 | 934 | L173R | Damaging | Probably Damaging | 0.5535 Pathological | 0.924 | Effect 85% | Disease | Probably Damaging |
| rs63750108 | NP_000242 | 934 | I708N | Damaging | Probably Damaging | 0.5492 Pathological | 0.768 | Effect 75% | Disease | Probably Damaging |
| rs63750124 | NP_000242 | 934 | I145M | Damaging | Benign | Neutral | 0.886 | Effect 66% | Neutral | Probably Begign |
| rs63750126 | NP_000242 | 934 | V161D | Damaging | Probably Damaging | Neutral | 0.888 | Effect 80% | Neutral | Probably Damaging |
| rs63750214 | NP_000242 | 934 | V163D | Damaging | Probably Damaging | Neutral | 0.951 | Effect 80% | Neutral | Probably Damaging |
| rs63750232 | NP_000242 | 934 | G692R | Damaging | Probably Damaging | 0.6782 Pathological | 0.985 | Effect 95% | Neutral | Probably Damaging |
| rs63750255 | NP_000242 | 934 | D167H | Damaging | Probably Damaging | 0.5759 Pathological | 0.965 | Effect 91% | Neutral | Probably Damaging |
| rs63750280 | NP_000242 | 934 | P622T | Damaging | Probably Damaging | Neutral | 0.985 | Effect 75% | Neutral | Probably Damaging |
| rs63750327 | NP_000242 | 934 | E198G | Damaging | Probably Damaging | 0.5638 Pathological | 0.969 | Effect 85% | Neutral | Probably Damaging |
| rs63750335 | NP_000242 | 934 | D49V | Damaging | Probably Damaging | Neutral | 0.892 | Effect 85% | Disease | Probably Damaging |
| rs63750368 | NP_000242 | 934 | A765T | Damaging | Probably Damaging | Neutral | 0.843 | Neutral | Neutral | Probably Damaging |
| rs63750379 | NP_000242 | 934 | Y408C | Damaging | Probably Damaging | Neutral | 0.642 | Effect 59% | Disease | Probably Damaging |
| rs63750381 | NP_000242 | 934 | D283Y | Damaging | Probably Damaging | Neutral | 0.852 | Effect 71% | Neutral | Possibly Damaging |
| rs63750398 | NP_000242 | 934 | C697F | Damaging | Probably Damaging | 0.6062 Pathological | 0.963 | Effect 91% | Disease | Probably Damaging |
| rs63750466 | NP_000242 | 934 | A2T | Damaging | Probably Damaging | Neutral | 0.706 | Neutral | Neutral | Probably Damaging |
| rs63750571 | NP_000242 | 934 | K845E | Damaging | Benign | Neutral | 0.815 | Effect 91% | Neutral | Probably Damaging |
| rs63750582 | NP_000242 | 934 | G164R | Damaging | Probably Damaging | 0.5242 Pathological | 0.911 | Effect 85% | Neutral | Probably Damaging |
| rs63750582 | NP_000242 | 934 | G164W | Damaging | Probably Damaging | 0.5382 Pathological | 0.731 | Effect 80% | Neutral | Probably Damaging |
| rs63750602 | NP_000242 | 934 | T335I | Damaging | Probably Damaging | Neutral | 0.926 | Effect 91% | Disease | Probably Damaging |
| rs63750623 | NP_000242 | 934 | Q824E | Damaging | Probably Damaging | Neutral | 0.692 | Effect 75% | Neutral | Probably Damaging |
| rs63750624 | NP_000242 | 934 | G162R | Damaging | Probably Damaging | Neutral | 0.972 | Effect 91% | Disease | Probably Damaging |
| rs63750626 | NP_000242 | 934 | K627N | Damaging | Possibly Damaging | Neutral | 0.420 | Neutral | Neutral | Probably Damaging |
| rs63750630 | NP_000242 | 934 | L330P | Damaging | Probably Damaging | Neutral | 0.930 | Effect 91% | Disease | Probably Damaging |
| rs63750640 | NP_000242 | 934 | L310P | Damaging | Probably Damaging | 0.7326 Pathological | 0.940 | Effect 91% | Disease | Probably Damaging |
| rs63750657 | NP_000242 | 934 | D603N | Damaging | Probably Damaging | Neutral | 0.982 | Effect 91% | Disease | Probably Damaging |
| rs63750657 | NP_000242 | 934 | D603Y | Damaging | Probably Damaging | Neutral | 0.936 | Effect 91% | Disease | Probably Damaging |
| rs63750665 | NP_000242 | 934 | A609V | Damaging | Probably Damaging | Neutral | 0.921 | Effect 71% | Neutral | Probably Damaging |
| rs63750757 | NP_000242 | 934 | A834T | Damaging | Probably Damaging | 0.6471 Pathological | 0.970 | Neutral | Disease | Probably Damaging |
| rs63750773 | NP_000242 | 934 | G162A | Damaging | Probably Damaging | Neutral | 0.970 | Effect 75% | Neutral | Probably Damaging |
| rs63750790 | NP_000242 | 934 | M688I | Damaging | Probably Damaging | Neutral | 0.962 | Effect 91% | Disease | Probably Damaging |
| rs63750794 | NP_000242 | 934 | S723F | Damaging | Probably Damaging | 0.8088 Pathological | 0.991 | Effect 91% | Disease | Probably Damaging |
| rs63750797 | NP_000242 | 934 | E853A | Damaging | Benign | 0.7096 Pathological | 0.451 | Neutral | Neutral | Probably Damaging |
| rs63750881 | NP_000242 | 934 | K246Q | Damaging | Benign | Neutral | 0.785 | Effect 63% | Neutral | Probably Damaging |
| rs63750887 | NP_000242 | 934 | Y98C | Damaging | Probably Damaging | 0.5607 Pathological | 0.811 | Neutral | Neutral | Probably Damaging |
| rs63750961 | NP_000242 | 934 | C697R | Damaging | Probably Damaging | 0.7212 Pathological | 0.979 | Effect 91% | Neutral | Probably Damaging |
| rs63751107 | NP_000242 | 934 | T33A | Damaging | Probably Damaging | Neutral | 0.517 | Neutral | Neutral | Probably Damaging |
| rs63751107 | NP_000242 | 934 | T33P | Damaging | Probably Damaging | Neutral | 0.896 | Effect 85% | Neutral | Probably Damaging |
| rs63751110 | NP_000242 | 934 | C199R | Damaging | Probably Damaging | 0.5914 Pathological | 0.960 | Effect 80% | Neutral | Probably Damaging |
| rs63751119 | NP_000242 | 934 | G751R | Damaging | Probably Damaging | 0.8374 Pathological | 0.998 | Effect 91% | Disease | Probably Damaging |
| rs63751125 | NP_000242 | 934 | T724K | Damaging | Probably Damaging | 0.8625 Pathological | 0.946 | Effect 91% | Disease | Probably Damaging |
| rs63751136 | NP_000242 | 934 | C199Y | Damaging | Probably Damaging | 0.5894 Pathological | 0.602 | Effect 80% | Neutral | Probably Damaging |
| rs63751173 | NP_000242 | 934 | Y103C | Damaging | Probably Damaging | 0.5247 Pathological | 0.636 | Effect 71% | Neutral | Probably Damaging |
| rs63751207 | NP_000242 | 934 | R524P | Damaging | Probably Damaging | 0.5076 Pathological | 0.864 | Effect 95% | Disease | Probably Damaging |
| rs63751224 | NP_000242 | 934 | A714V | Damaging | Probably Damaging | Neutral | 0.957 | Effect 71% | Disease | Probably Damaging |
| rs63751236 | NP_000242 | 934 | A600V | Damaging | Probably Damaging | Neutral | 0.954 | Effect 71% | Disease | Probably Damaging |
| rs63751291 | NP_000242 | 934 | L175P | Damaging | Probably Damaging | Neutral | 0.951 | Effect 80% | Neutral | Probably Begign |
| rs63751429 | NP_000242 | 934 | L93F | Damaging | Probably Damaging | Neutral | 0.901 | Effect 63% | Neutral | Probably Damaging |
| rs63751432 | NP_000242 | 934 | G692V | Damaging | Probably Damaging | Neutral | 0.973 | Effect 91% | Disease | Probably Damaging |
| rs63751444 | NP_000242 | 934 | L187P | Damaging | Probably Damaging | 0.5041 Pathological | 0.952 | Effect 85% | Disease | Probably Damaging |
| rs63751444 | NP_000242 | 934 | L187R | Damaging | Probably Damaging | Neutral | 0.910 | Effect 80% | Disease | Probably Damaging |
| rs63751455 | NP_000242 | 934 | R243Q | Damaging | Probably Damaging | 0.5083 Pathological | 0.400 | Effect 63% | Neutral | Probably Damaging |
| rs35717997 | NP_000242 | 934 | P415S | Damaging | Possibly Damaging | Neutral | 0.576 | Effect 59% | Disease | Probably Damaging |
| rs35784190 | NP_000242 | 934 | T782A | Damaging | Probably Damaging | Neutral | 0.950 | Effect 85% | Disease | Probably Damaging |
| rs61756465 | NP_000242 | 934 | K392M | Damaging | Probably Damaging | Neutral | 0.573 | Effect 53% | Neutral | Probably Damaging |
| rs63750485 | NP_000242 | 934 | L387P | Damaging | Probably Damaging | Neutral | 0.871 | Effect 91% | Disease | Probably Damaging |
| rs63751409 | NP_000242 | 934 | F694L | Damaging | Probably Damaging | Neutral | 0.869 | Effect 85% | Neutral | Probably Damaging |
| rs63751424 | NP_000242 | 934 | V34E | Damaging | Possibly Damaging | Neutral | 0.895 | Effect 80% | Neutral | Possibly Damaging |
| rs63751456 | NP_000242 | 934 | L93P | Damaging | Probably Damaging | Neutral | 0.914 | Effect 80% | Disease | Probably Damaging |
