# Supplementary material for: Investigation of pathogenic germline variants in gastric cancer and development of “GasCanBase” database
Source: Cancer Rep (Hoboken). 2023 Oct 22;6(12):e1906. doi: 10.1002/cnr2.1906 (PMC10728505; doi:10.1002/cnr2.1906)
Supplement: Supplementary file 1 — Data S1 Supporting Information. [file CNR2-6-e1906-s001.zip › Supplementary File/Table S71. Prediction of damaging effect on MTHFR.docx]

Table S71. Prediction of damaging effect on MTHFR

| **SNP** | **Protein ID** | **Amino acid** | **Amino acid change** | **SIFT** | **PolyPhen2** | **PMut** | **MutPred** | **SNAP2** | **SNP&GO** | **PANTHER** |
| --- | --- | --- | --- | --- | --- | --- | --- | --- | --- | --- |
| rs121434295 | NP_005948 | 656 | R157Q | Damaging | Probably Damaging | 0.5841 Pathological | 0.987 | Effect 91% | Disease | No Panther Family For Input Sequence |
| rs45438591 | NP_005948 | 656 | G221R | Damaging | Probably Damaging | Neutral | 0.929 | Effect 91% | Disease | No Panther Family For Input Sequence |
| rs1801133 | NP_005948 | 656 | A222V | Damaging | Probably Damaging | Neutral | 0.329 | Effect 95% | Disease | No Panther Family For Input Sequence |
| rs121434297 | NP_005948 | 656 | L323P | Damaging | Probably Damaging | Neutral | 0.980 | Effect 95% | Disease | No Panther Family For Input Sequence |
| rs121434296 | NP_005948 | 656 | R377C | Damaging | Probably Damaging | 0.8467 Pathological | 0.943 | Effect 91% | Disease | No Panther Family For Input Sequence |
| rs116620395 | NP_005948 | 656 | W500C | Damaging | Probably Damaging | 0.8706 Pathological | 0.866 | Effect 80% | Disease | No Panther Family For Input Sequence |
| rs2274974 | NP_005948 | 656 | G566E | Damaging | Probably Damaging | 0.8596 Pathological | 0.305 | Effect 85% | Disease | No Panther Family For Input Sequence |
| rs45449298 | NP_005948 | 656 | R519H | Damaging | Benign | Neutral | 0.110 | Effect 71% | Neutral | No Panther Family For Input Sequence |
| rs2066472 | NP_005948 | 656 | R68Q | Damaging | Probably Damaging | Neutral | 0.268 | Effect 66% | Neutral | No Panther Family For Input Sequence |
| rs45496998 | NP_005948 | 656 | R519C | Damaging | Probably Damaging | 0.7962 Pathological | 0.176 | Effect 91% | Neutral | No Panther Family For Input Sequence |
| rs45550133 | NP_005948 | 656 | R134C | Damaging | Possibly Damaging | 0.5723 Pathological | 0.600 | Neutral | Neutral | No Panther Family For Input Sequence |
| rs45571736 | NP_005948 | 656 | G422R | Damaging | Probably Damaging | 0.7257 Pathological | 0.219 | Effect 91% | Neutral | No Panther Family For Input Sequence |
| rs45589033 | NP_005948 | 656 | D234N | Damaging | Benign | Neutral | 0.723 | Effect 59% | Neutral | No Panther Family For Input Sequence |
| rs56182143 | NP_005948 | 656 | R325H | Damaging | Probably Damaging | 0.6407 Pathological | 0.784 | Effect 63% | Neutral | No Panther Family For Input Sequence |
| rs72552099 | NP_005948 | 656 | S430R | Damaging | Possibly Damaging | 0.7443 Pathological | 0.535 | Effect 63% | Neutral | No Panther Family For Input Sequence |
