# Supplementary material for: Investigation of pathogenic germline variants in gastric cancer and development of “GasCanBase” database
Source: Cancer Rep (Hoboken). 2023 Oct 22;6(12):e1906. doi: 10.1002/cnr2.1906 (PMC10728505; doi:10.1002/cnr2.1906)
Supplement: Supplementary file 1 — Data S1 Supporting Information. [file CNR2-6-e1906-s001.zip › Supplementary File/Table S73. Prediction of damaging effect on MYC.docx]

Table S73. Prediction of damaging effect on MYC

| **SNP** | **Protein ID** | **Amino acid** | **Amino acid change** | **SIFT** | **PolyPhen2** | **PMut** | **MutPred** | **SNAP2** | **SNP&GO** | **PANTHER** |
| --- | --- | --- | --- | --- | --- | --- | --- | --- | --- | --- |
| rs28933407 | NP_002458 | 454 | P72S | Damaging | Possibly Damaging | Neutral | 0.630 | Effect 75% | Disease | Probably Damaging |
| rs121918683 | NP_002458 | 454 | N101T | Damaging | Benign | Neutral | 0.332 | Neutral | Neutral | Probably Damaging |
| rs121918684 | NP_002458 | 454 | E54D | Damaging | Benign | Neutral | 0.284 | Effect 59% | Neutral | Probably Damaging |
| rs121918685 | NP_002458 | 454 | P74A | Damaging | Probably Damaging | Neutral | 0.711 | Effect 71% | Disease | Probably Damaging |
| rs4645959 | NP_002458 | 454 | N26S | Damaging | Possibly Damaging | Neutral | 0.805 | Effect 59% | Disease | Probably Damaging |
| rs4645960 | NP_002458 | 454 | G175C | Damaging | Benign | Neutral | 0.486 | Effect 63% | Neutral | Probably Damaging |
| rs4645961 | NP_002458 | 454 | V185I | Damaging | Possibly Damaging | Neutral | 0.392 | Neutral | Neutral | Probably Damaging |
| rs4645968 | NP_002458 | 454 | A337V | Damaging | Benign | Neutral | 0.442 | Neutral | Neutral | Probably Damaging |
| rs61755060 | NP_002458 | 454 | Q168H | Damaging | Benign | Neutral | 0.543 | Neutral | Neutral | Probably Damaging |
| rs112602073 | NP_002458 | 454 | P220S | Damaging | Benign | Neutral | 0.290 | Neutral | Neutral | Probably Damaging |
