# Supplementary material for: Investigation of pathogenic germline variants in gastric cancer and development of “GasCanBase” database
Source: Cancer Rep (Hoboken). 2023 Oct 22;6(12):e1906. doi: 10.1002/cnr2.1906 (PMC10728505; doi:10.1002/cnr2.1906)
Supplement: Supplementary file 1 — Data S1 Supporting Information. [file CNR2-6-e1906-s001.zip › Supplementary File/Table S75. Prediction of damaging effect on PIK3CA.docx]

Table S75. Prediction of damaging effect on PIK3CA

| **SNP** | **Protein ID** | **Amino acid** | **Amino acid change** | **SIFT** | **PolyPhen2** | **PMut** | **MutPred** | **SNAP2** | **SNP&GO** | **PANTHER** |
| --- | --- | --- | --- | --- | --- | --- | --- | --- | --- | --- |
| rs3908109 | NP_006209 | 1068 | D626N | Damaging | Possibly Damaging | Neutral | 0.765 | Effect 66% | Neutral | Probably Damaging |
| rs104886000 | NP_006209 | 1068 | R555K | Damaging | Probably Damaging | Neutral | 0.811 | Effect 80% | Disease | Probably Damaging |
| rs104886003 | NP_006209 | 1068 | E545K | Damaging | Probably Damaging | 0.6723 Pathological | 0.730 | Effect 59% | Neutral | Probably Damaging |
| rs121913272 | NP_006209 | 1068 | C420R | Damaging | Probably Damaging | 0.8560 Pathological | 0.726 | Neutral | Neutral | Probably Damaging |
| rs121913273 | NP_006209 | 1068 | E542K | Damaging | Probably Damaging | 0.6860 Pathological | 0.719 | Neutral | Neutral | Probably Damaging |
| rs121913274 | NP_006209 | 1068 | E545A | Damaging | Probably Damaging | Neutral | 0.769 | Neutral | Neutral | Probably Damaging |
| rs121913283 | NP_006209 | 1068 | M1043I | Damaging | Benign | Neutral | 0.537 | Effect 63% | Neutral | Probably Damaging |
| rs121913284 | NP_006209 | 1068 | N345K | Damaging | Probably Damaging | Neutral | 0.604 | Effect 75% | Neutral | Probably Damaging |
| rs121913288 | NP_006209 | 1068 | Y1021C | Damaging | Probably Damaging | 0.9270 Pathological | 0.445 | Neutral | Disease | Probably Damaging |
| rs3729687 | NP_006209 | 1068 | E707K | Damaging | Probably Damaging | 0.8120 Pathological | 0.448 | Neutral | Neutral | Probably Damaging |
| rs3865687 | NP_006209 | 1068 | R617Q | Damaging | Possibly Damaging | 0.6381 Pathological | 0.563 | Neutral | Neutral | Probably Damaging |
| rs17849072 | NP_006209 | 1068 | Y644H | Damaging | Probably Damaging | Neutral | 0.608 | Effect 75% | Disease | Probably Damaging |
| rs71310379 | NP_006209 | 1068 | Q60K | Damaging | Benign | Neutral | 0.490 | Neutral | Neutral | Possibly Damaging |
| rs74427133 | NP_006209 | 1068 | N873T | Damaging | Probably Damaging | 0.6063 Pathological | 0.572 | Neutral | Neutral | Probably Damaging |
| rs3914675 | NP_001157149 | 173 | R152H | Damaging | Benign | 0.7715 Pathological | 0.532 | Effect 71% | Neutral | Not Scored |
| rs121434592 | NP_001014431 | 480 | E17K | Damaging | Probably Damaging | 0.6103 Pathological | 0.621 | Effect 95% | Neutral | Probably Benign |
