# Supplementary material for: Investigation of pathogenic germline variants in gastric cancer and development of “GasCanBase” database
Source: Cancer Rep (Hoboken). 2023 Oct 22;6(12):e1906. doi: 10.1002/cnr2.1906 (PMC10728505; doi:10.1002/cnr2.1906)
Supplement: Supplementary file 1 — Data S1 Supporting Information. [file CNR2-6-e1906-s001.zip › Supplementary File/Table S76. Prediction of damaging effect on PTEN.docx]

Table S76. Prediction of damaging effect on PTEN

| **SNP** | **Protein ID** | **Amino acid** | **Amino acid change** | **SIFT** | **PolyPhen2** | **PMut** | **MutPred** | **SNAP2** | **SNP&GO** | **PANTHER** |
| --- | --- | --- | --- | --- | --- | --- | --- | --- | --- | --- |
| rs121909233 | NP_000305 | 403 | D19N | Damaging | Probably Damaging | Neutral | 0.434 | Effect 85% | Disease | Probably Damaging |
| rs121909225 | NP_000305 | 403 | M35R | Damaging | Probably Damaging | 0.8955 Pathological | 0.969 | Effect  95% | Disease | Probably Damaging |
| rs121909236 | NP_000305 | 403 | H61D | Damaging | Probably Damaging | 0.7597 Pathological | 0.803 | Effect 85% | Disease | Probably Damaging |
| rs121909226 | NP_000305 | 403 | L70P | Damaging | Probably Damaging | Neutral | 0.833 | Effect 95% | Disease | Probably Damaging |
| rs121909238 | NP_000305 | 403 | H93R | Damaging | Probably Damaging | 0.7705 Pathological | 0.879 | Effect 91% | Disease | Probably Damaging |
| rs121909230 | NP_000305 | 403 | L112P | Damaging | Probably Damaging | Neutral | 0.915 | Effect 95% | Disease | Probably Damaging |
| rs121909222 | NP_000305 | 403 | H123R | Damaging | Probably Damaging | 0.7247  Pathological | 0.955 | Effect 95% | Disease | Probably Damaging |
| rs121909223 | NP_000305 | 403 | C124R | Damaging | Probably Damaging | 0.9310 Pathological | 0.941 | Effect 95% | Disease | Probably Damaging |
| rs121909218 | NP_000305 | 403 | G129E | Damaging | Probably Damaging | 0.9413 Pathological | 0.923 | Effect 95% | Disease | Probably Damaging |
| rs121909229 | NP_000305 | 403 | R130Q | Damaging | Probably Damaging | 0.5642 Pathological | 0.980 | Effect 95% | Disease | Probably Damaging |
| rs121909241 | NP_000305 | 403 | G132V | Damaging | Probably Damaging | 0.5759 Pathological | 0.977 | Effect 95% | Disease | Probably Damaging |
| rs121909221 | NP_000305 | 403 | S170R | Damaging | Probably Damaging | 0.7180 Pathological | 0.895 | Effect 95% | Disease | Probably Damaging |
| rs121913293 | NP_000305 | 403 | R173C | Damaging | Probably Damaging | 0.8956 Pathological | 0.964 | Effect 71% | Disease | Probably Damaging |
| rs121913294 | NP_000305 | 403 | R173H | Damaging | Probably Damaging | 0.7013 Pathological | 0.955 | Effect 75% | Disease | Probably Damaging |
| rs121909240 | NP_000305 | 403 | F241S | Damaging | Benign | 0.5184 Pathological | 0.721 | Effect 91% | Disease | Probably Damaging |
| rs121909239 | NP_000305 | 403 | D252G | Damaging | Probably Damaging | 0.8053 Pathological | 0.849 | Effect 95% | Disease | Probably Damaging |
| rs121909235 | NP_000305 | 403 | R234Q | Damaging | Probably Damaging | 0.6195 Pathological | 0.441 | Effect 80% | Neutral | Probably Damaging |
| rs121909237 | NP_000305 | 403 | A121G | Damaging | Probably Damaging | Neutral | 0.773 | Effect 95% | Neutral | Probably Damaging |
| rs57374291 | NP_000305 | 403 | D107N | Damaging | Probably Damaging | Neutral | 0.402 | Neutral | Neutral | Probably Damaging |
