# Supplementary material for: Investigation of pathogenic germline variants in gastric cancer and development of “GasCanBase” database
Source: Cancer Rep (Hoboken). 2023 Oct 22;6(12):e1906. doi: 10.1002/cnr2.1906 (PMC10728505; doi:10.1002/cnr2.1906)
Supplement: Supplementary file 1 — Data S1 Supporting Information. [file CNR2-6-e1906-s001.zip › Supplementary File/Table S77. Prediction of damaging effect on PTGS2.docx]

Table S77. Prediction of damaging effect on PTGS2

| **SNP** | **Protein ID** | **Amino acid** | **Amino acid change** | **SIFT** | **PolyPhen2** | **PMut** | **MutPred** | **SNAP2** | **SNP&GO** | **PANTHER** |
| --- | --- | --- | --- | --- | --- | --- | --- | --- | --- | --- |
| rs5272 | NP_000954 | 604 | E488G | Damaging | Probably Damaging | 0.5936 Pathological | 0.507 | Effect 91% | Disease | Probably Damaging |
| rs20426 | NP_000954 | 604 | M1I | Damaging | Benign | Neutral | 0.728 | Neutral | Neutral | Probably Damaging |
