# Supplementary material for: Investigation of pathogenic germline variants in gastric cancer and development of “GasCanBase” database
Source: Cancer Rep (Hoboken). 2023 Oct 22;6(12):e1906. doi: 10.1002/cnr2.1906 (PMC10728505; doi:10.1002/cnr2.1906)
Supplement: Supplementary file 1 — Data S1 Supporting Information. [file CNR2-6-e1906-s001.zip › Supplementary File/Table S79. Prediction of damaging effect on SDHA.docx]

Table S79. Prediction of damaging effect on SDHA

| **SNP** | **Protein ID** | **Amino acid** | **Amino acid change** | **SIFT** | **PolyPhen2** | **PMut** | **MutPred** | **SNAP2** | **SNP&GO** | **PANTHER** |
| --- | --- | --- | --- | --- | --- | --- | --- | --- | --- | --- |
| rs61754481 | NP_004159 | 664 | Y205H | Damaging | Probably Damaging | Neutral | 0.806 | Effect 85% | Neutral | Probably Damaging |
| rs6960 | NP_004159 | 664 | Y629F | Damaging | Benign | Neutral | 0.564 | Neutral | Neutral | Probably Damaging |
| rs1061517 | NP_004159 | 664 | M1V | Damaging | Benign | Neutral | 0.985 | Neutral | Neutral | Possibly Damaging |
| rs11557099 | NP_004159 | 664 | K128N | Damaging | Probably Damaging | Neutral | 0.776 | Effect 75% | Disease | Probably Damaging |
| rs41495051 | NP_004159 | 664 | G229R | Damaging | Possibly Damaging | 0.6482 Pathological | 0.643 | Effect 66% | Disease | Probably Damaging |
| rs76896145 | NP_004159 | 664 | S456L | Damaging | Probably Damaging | Neutral | 0.877 | Effect 59% | Disease | Probably Damaging |
| rs1126557 | NP_004159 | 664 | D596G | Damaging | Possibly Damaging | 0.7365 Pathological | 0.928 | Effect 71% | Disease | Probably Damaging |
| rs1126568 | NP_004159 | 664 | R600Q | Damaging | Probably Damaging | 0.7297 Pathological | 0.880 | Effect 91% | Disease | Probably Damaging |
| rs3211483 | NP_004159 | 664 | V644M | Damaging | Probably Damaging | 0.5084 Pathological | 0.906 | Effect 71% | Disease | Probably Damaging |
| rs80207011 | NP_004159 | 664 | D49G | Damaging | Benign | Neutral | 0.350 | Neutral | Neutral | Probably Damaging |
| rs13992 | NP_004159 | 664 | R623K | Damaging | Possibly Damaging | Neutral | 0.645 | Neutral | Neutral | Probably Damaging |
| rs1041946 | NP_004159 | 664 | E240Q | Damaging | Possibly Damaging | Neutral | 0.179 | Neutral | Neutral | Probably Damaging |
| rs1042052 | NP_004159 | 664 | I579V | Damaging | Benign | Neutral | 0.536 | Neutral | Neutral | Probably Damaging |
| rs1042457 | NP_004159 | 664 | R662H | Damaging | Probably Damaging | 0.8364 Pathological | 0.796 | Effect 91% | Neutral | Probably Damaging |
| rs1061518 | NP_004159 | 664 | F33V | Damaging | Benign | Neutral | 0.338 | Neutral | Neutral | Probably Damaging |
| rs1061520 | NP_004159 | 664 | K41E | Damaging | Benign | Neutral | 0.410 | Neutral | Neutral | Probably Damaging |
| rs1062468 | NP_004159 | 664 | V333I | Damaging | Benign | Neutral | 0.128 | Neutral | Neutral | Probably Damaging |
| rs1126697 | NP_004159 | 664 | L639V | Damaging | Benign | 0.5571 Pathological | 0.619 | Neutral | Neutral | Probably Damaging |
| rs60587941 | NP_004159 | 664 | C654G | Damaging | Possibly Damaging | 0.9490 Pathological | 0.555 | Effect 80% | Neutral | Probably Damaging |
| rs111387770 | NP_004159 | 664 | A466T | Damaging | Benign | Neutral | 0.491 | Neutral | Neutral | Probably Damaging |
| rs111540173 | NP_004159 | 664 | I661T | Damaging | Benign | 0.7338 Pathological | 0.432 | Neutral | Neutral | Probably Damaging |
| rs112937607 | NP_004159 | 664 | I661V | Damaging | Benign | Neutral | 0.388 | Neutral | Neutral | Probably Damaging |
