# Supplementary material for: Investigation of pathogenic germline variants in gastric cancer and development of “GasCanBase” database
Source: Cancer Rep (Hoboken). 2023 Oct 22;6(12):e1906. doi: 10.1002/cnr2.1906 (PMC10728505; doi:10.1002/cnr2.1906)
Supplement: Supplementary file 1 — Data S1 Supporting Information. [file CNR2-6-e1906-s001.zip › Supplementary File/Table S80. Prediction of damaging effect on SDHB.docx]

Table S80. Prediction of damaging effect on SDHB

| **SNP** | **Protein ID** | **Amino acid** | **Amino acid change** | **SIFT** | **PolyPhen2** | **PMut** | **MutPred** | **SNAP2** | **SNP&GO** | **PANTHER** |
| --- | --- | --- | --- | --- | --- | --- | --- | --- | --- | --- |
| rs11203289 | NP_002991 | 280 | A3G | Damaging | Benign | Neutral | 0.702 | Neutral | Neutral | Probably Damaging |
| rs33927012 | NP_002991 | 280 | S163P | Damaging | Benign | Neutral | 0.140 | Effect 71% | Neutral | Probably Damaging |
| rs34599281 | NP_002991 | 280 | T60A | Damaging | Benign | Neutral | 0.310 | Neutral | Neutral | Probably Damaging |
| rs34916635 | NP_002991 | 280 | G53E | Damaging | Benign | 0.5701 Pathological | 0.412 | Neutral | Neutral | Probably Damaging |
| rs74315370 | NP_002991 | 280 | R46G | Damaging | Probably Damaging | Neutral | 0.934 | Effect 95% | Disease | Probably Damaging |
| rs74315371 | NP_002991 | 280 | C101Y | Damaging | Probably Damaging | 0.5576 Pathological | 0.944 | Effect 95% | Disease | Probably Damaging |
| rs74315372 | NP_002991 | 280 | H132P | Damaging | Probably Damaging | Neutral | 0.874 | Effect 95% | Disease | Probably Damaging |
| rs74315367 | NP_002991 | 280 | P197R | Damaging | Probably Damaging | Neutral | 0.957 | Effect 95% | Disease | Probably Damaging |
| rs111430410 | NP_002991 | 280 | R11H | Damaging | Possibly Damaging | Neutral | 0.775 | Effect 53% | Neutral | Probably Damaging |
| rs11541234 | NP_002991 | 280 | T119I | Damaging | Benign | Neutral | 0.282 | Neutral | Neutral | Probably Damaging |
