# Supplementary material for: Investigation of pathogenic germline variants in gastric cancer and development of “GasCanBase” database
Source: Cancer Rep (Hoboken). 2023 Oct 22;6(12):e1906. doi: 10.1002/cnr2.1906 (PMC10728505; doi:10.1002/cnr2.1906)
Supplement: Supplementary file 1 — Data S1 Supporting Information. [file CNR2-6-e1906-s001.zip › Supplementary File/Table S81. Prediction of damaging effect on SDHD.docx]

Table S81. Prediction of damaging effect on SDHD

| **SNP** | **Protein ID** | **Amino acid** | **Amino acid change** | **SIFT** | **PolyPhen2** | **PMut** | **MutPred** | **SNAP2** | **SNP&GO** | **PANTHER** |
| --- | --- | --- | --- | --- | --- | --- | --- | --- | --- | --- |
| rs11214077 | NP_002993 | 159 | H50R | Damaging | Probably Damaging | 0.5434 Pathological | 0.220 | Effect 91% | Neutral | Probably Damaging |
| rs80338842 | NP_002993 | 159 | M1I | Damaging | Probably Damaging | Neutral | 0.978 | Effect 85% | Neutral | Probably Damaging |
| rs80338844 | NP_002993 | 159 | P81L | Damaging | Probably Damaging | 0.6465 Pathological | 0.937 | Effect 95% | Neutral | Probably Damaging |
| rs80338845 | NP_002993 | 159 | D92Y | Damaging | Probably Damaging | 0.9094 Pathological | 0.961 | Effect 95% | Disease | Probably Damaging |
| rs80338846 | NP_002993 | 159 | L95P | Damaging | Possibly Damaging | 0.8740 Pathological | 0.967 | Effect 91% | Disease | Probably Damaging |
| rs104894302 | NP_002993 | 159 | H102L | Damaging | Probably Damaging | 0.9045 Pathological | 0.988 | Effect 95% | Disease | Probably Damaging |
| rs104894304 | NP_002993 | 159 | Y114C | Damaging | Probably Damaging | 0.8810 Pathological | 0.983 | Effect 95% | Disease | Probably Damaging |
| rs80338847 | NP_002993 | 159 | L139P | Damaging | Probably Damaging | 0.9174 Pathological | 0.935 | Effect 95% | Disease | Probably Damaging |
| rs121908984 | NP_002993 | 159 | H145N | Damaging | Benign | Neutral | 0.810 | Effect 80% | Neutral | Possibly Damaging |
| rs11547889 | NP_002993 | 159 | R6G | Damaging | Possibly Damaging | 0.7901 Pathological | 0.700 | Effect 85% | Neutral | Probably Damaging |
| rs116405897 | NP_036591 | 98 | R62H | Damaging | Benign | 0.6547 Pathological | 0.429 | Effect 71% | Neutral | Possibly Damaging |
