# Supplementary material for: Investigation of pathogenic germline variants in gastric cancer and development of “GasCanBase” database
Source: Cancer Rep (Hoboken). 2023 Oct 22;6(12):e1906. doi: 10.1002/cnr2.1906 (PMC10728505; doi:10.1002/cnr2.1906)
Supplement: Supplementary file 1 — Data S1 Supporting Information. [file CNR2-6-e1906-s001.zip › Supplementary File/Table S82. Prediction of damaging effect on SMAD4.docx]

Table S82. Prediction of damaging effect on SMAD4

| **SNP** | **Protein ID** | **Amino acid** | **Amino acid change** | **SIFT** | **PolyPhen2** | **PMut** | **MutPred** | **SNAP2** | **SNP&GO** | **PANTHER** |
| --- | --- | --- | --- | --- | --- | --- | --- | --- | --- | --- |
| rs80338963 | NP_005350 | 552 | R361C | Damaging | Probably Damaging | 0.8711 Pathological | 0.994 | Effect 95% | Disease | Probably Damaging |
| rs61751988 | NP_005350 | 552 | A190P | Damaging | Benign | Neutral | 0.193 | Neutral | Neutral | Probably Damaging |
| rs75667697 | NP_005350 | 552 | L229R | Damaging | Possibly Damaging | 0.5277 Pathological | 0.287 | Effect 66% | Neutral | Probably Damaging |
