# Supplementary material for: Investigation of pathogenic germline variants in gastric cancer and development of “GasCanBase” database
Source: Cancer Rep (Hoboken). 2023 Oct 22;6(12):e1906. doi: 10.1002/cnr2.1906 (PMC10728505; doi:10.1002/cnr2.1906)
Supplement: Supplementary file 1 — Data S1 Supporting Information. [file CNR2-6-e1906-s001.zip › Supplementary File/Table S84. Prediction of damaging effect on TNF.docx]

Table S84. Prediction of damaging effect on TNF

| **SNP** | **Protein ID** | **Amino acid** | **Amino acid change** | **SIFT** | **PolyPhen2** | **PMut** | **MutPred** | **SNAP2** | **SNP&GO** | **PANTHER** |
| --- | --- | --- | --- | --- | --- | --- | --- | --- | --- | --- |
| rs1800620 | NP_000585 | 233 | A94T | Damaging | Benign | Neutral | 0.183 | Effect 63% | Neutral | Probably Benign |
| rs3179060 | NP_000585 | 233 | H52N | Damaging | Benign | Neutral | 0.730 | Neutral | Neutral | Probably Benign |
| rs4645843 | NP_000585 | 233 | P84L | Damaging | Benign | Neutral | 0.100 | Neutral | Neutral | Probably Benign |
| rs11574936 | NP_000585 | 233 | I194N | Damaging | Probably Damaging | 0.9720 Pathological | 0.861 | Effect 80% | Disease | Probably Benign |
| rs35131721 | NP_000585 | 233 | P64L | Damaging | Benign | Neutral | 0.341 | Neutral | Neutral | Probably Benign |
