# Supplementary material for: Investigation of pathogenic germline variants in gastric cancer and development of “GasCanBase” database
Source: Cancer Rep (Hoboken). 2023 Oct 22;6(12):e1906. doi: 10.1002/cnr2.1906 (PMC10728505; doi:10.1002/cnr2.1906)
Supplement: Supplementary file 1 — Data S1 Supporting Information. [file CNR2-6-e1906-s001.zip › Supplementary File/Table S85. Prediction of damaging effect on TP53.docx]

Table S85. Prediction of damaging effect on TP53

| **SNP** | **Protein ID** | **Amino acid** | **Amino acid change** | **SIFT** | **PolyPhen2** | **PMut** | **MutPred** | **SNAP2** | **SNP&GO** | **PANTHER** |
| --- | --- | --- | --- | --- | --- | --- | --- | --- | --- | --- |
| rs28934875 | NP_000537 | 393 | A138P | Damaging | Probably Damaging | 0.6104 Pathological | 0.952 | Effect 85% | Disease | Probably Damaging |
| rs28934874 | NP_000537 | 393 | P151T | Damaging | Possibly Damaging | 0.6315 Pathological | 0.959 | Effect 75% | Disease | Probably Damaging |
| rs28934578 | NP_000537 | 393 | R175H | Damaging | Possibly Damaging | 0.7544 Pathological | 0.986 | Effect 95% | Disease | Probably Damaging |
| rs28934573 | NP_000537 | 393 | S241F | Damaging | Probably Damaging | 0.8829 Pathological | 0.988 | Effect 95% | Disease | Probably Benign |
| rs28934575 | NP_000537 | 393 | G245S | Damaging | Probably Damaging | 0.8179 Pathological | 0.995 | Effect 95% | Disease | Probably Damaging |
| rs11540652 | NP_000537 | 393 | R248Q | Damaging | Probably Damaging | 0.7506 Pathological | 0.993 | Effect 95% | Disease | Probably Damaging |
| rs28934571 | NP_000537 | 393 | R249S | Damaging | Probably Damaging | 0.7552 Pathological | 0.954 | Effect 95% | Disease | Probably Damaging |
| rs28934577 | NP_000537 | 393 | L257Q | Damaging | Probably Damaging | 0.7712 Pathological | 0.966 | Effect 80% | Disease | Probably Benign |
| rs55832599 | NP_000537 | 393 | R267W | Damaging | Probably Damaging | 0.9586 Pathological | 0.911 | Effect 85% | Disease | Probably Damaging |
| rs121913343 | NP_000537 | 393 | R273C | Damaging | Probably Damaging | 0.7628 Pathological | 0.987 | Effect 95% | Disease | Probably Damaging |
| rs121913343 | NP_000537 | 393 | R273H | Damaging | Possibly Damaging | 0.7885 Pathological | 0.991 | Effect 95% | Disease | Probably Damaging |
| rs17849781 | NP_000537 | 393 | P278A | Damaging | Probably Damaging | Neutral | 0.956 | Effect 71% | Disease | Probably Damaging |
| rs28934574 | NP_000537 | 393 | R282W | Damaging | Probably Damaging | 0.9719 Pathological | 0.953 | Effect 95% | Disease | Probably Damaging |
| rs3021068 | NP_001119586 | 341 | C341S | Damaging | Benign | Neutral | 0.230 | Effect 53% | Neutral | Cannot Score Substitution |
| rs11540654 | NP_000537 | 393 | R110L | Damaging | Benign | 0.8057 Pathological | 0.837 | Effect 3% | Neutral | Probably Benign |
| rs11540654 | NP_000537 | 393 | R110P | Damaging | Possibly Damaging | 0.8391 Pathological | 0.650 | Effect 66% | Neutral | Probably Benign |
| rs17881470 | NP_000537 | 393 | S366A | Damaging | Benign | Neutral | 0.161 | Neutral | Neutral | Probably Benign |
| rs17882252 | NP_000537 | 393 | E339K | Damaging | Benign | 0.8973 Pathological | 0.677 | Neutral | Neutral | Possibly Damaging |
| rs28934873 | NP_000537 | 393 | M133T | Damaging | Benign | Neutral | 0.916 | Effect 66% | Neutral | Probably Benign |
| rs35163653 | NP_000537 | 393 | V217M | Damaging | Possibly Damaging | Neutral | 0.245 | Neutral | Neutral | Probably Benign |
| rs72661119 | NP_000537 | 393 | N263D | Damaging | Benign | Neutral | 0.463 | Neutral | Neutral | Probably Benign |
| rs1642789 | NP_001119585 | 346 | C339S | Damaging | Benign | Neutral | 0.668 | Neutral | Neutral | Cannot Score Substitution |
| rs17849781 | NP_000537 | 393 | P278A | Damaging | Probably Damaging | Neutral | 0.956 | Effect 71% | Neutral | Probably Damaging |
| rs17880282 | NP_001137462 | 548 | P11S | Damaging | Benign | Neutral | 0.064 | Effect 85% | Neutral | Probably Benign |
| rs72661117 | NP_000537 | 393 | D184N | Damaging | Benign | Neutral | 0.481 | Neutral | Neutral | Probably Benign |
| rs80184930 | NP_000537 | 393 | S378P | Damaging | Benign | 0.8992 Pathological | 0.127 | Neutral | Neutral | Probably Benign |
| rs111897235 | NP_001137462 | 548 | A59T | Damaging | Benign | Neutral | 0.224 | Neutral | Neutral | Probably Benign |
| rs112431538 | NP_000537 | 393 | E285K | Damaging | Probably Damaging | 0.6357 Pathological | 0.763 | Effect 91% | Neutral | Probably Damaging |
