# Supplementary material for: Investigation of pathogenic germline variants in gastric cancer and development of “GasCanBase” database
Source: Cancer Rep (Hoboken). 2023 Oct 22;6(12):e1906. doi: 10.1002/cnr2.1906 (PMC10728505; doi:10.1002/cnr2.1906)
Supplement: Supplementary file 1 — Data S1 Supporting Information. [file CNR2-6-e1906-s001.zip › Supplementary File/Table S86. Prediction of damaging effect on VEGFA.docx]

Table S86. Prediction of damaging effect on VEGFA

| **SNP** | **Protein ID** | **Amino acid** | **Amino acid change** | **SIFT** | **PolyPhen2** | **PMut** | **MutPred** | **SNAP2** | **SNP&GO** | **PANTHER** |
| --- | --- | --- | --- | --- | --- | --- | --- | --- | --- | --- |
| rs62401172 | NP_001020537 | 412 | G365V | Damaging | Probably Damaging | 0.5934 Pathological | 0.291 | Neutral | Neutral | Probably Benign |
| rs76869573 | NP_001020537 | 412 | P312T | Damaging | Probably Damaging | Neutral | 0.358 | Effect  66% | Neutral | Probably Damaging |
| rs114262569 | NP_001020537 | 412 | R288W | Damaging | Probably Damaging | 0.9757 Pathological | 0.603 | Effect  75% | Neutral | Possibly Damaging |
