# Supplementary material for: Investigation of pathogenic germline variants in gastric cancer and development of “GasCanBase” database
Source: Cancer Rep (Hoboken). 2023 Oct 22;6(12):e1906. doi: 10.1002/cnr2.1906 (PMC10728505; doi:10.1002/cnr2.1906)
Supplement: Supplementary file 1 — Data S1 Supporting Information. [file CNR2-6-e1906-s001.zip › Supplementary File/Table S87. List of domains of cancer Gene.docx]

Table: Domain association

| Gene Name | Protein ID | Name of Domain | Domain ID | Description | Location in Protein Sequence |
| --- | --- | --- | --- | --- | --- |
| ABCB1 | NP_000918.2 | **ABC_TM1F** | [PS50929](https://prosite.expasy.org/cgi-bin/prosite/nicedoc.pl?PS50929) | ABC transporter integral membrane type-1 fused domain | **52 – 357**  **712 - 1000** |
|  |  | **ABC_TRANSPORTER_2** | [PS50893](https://prosite.expasy.org/cgi-bin/prosite/nicedoc.pl?PS50893) | *ATP-binding cassette, ABC transporter-type domain* | **392 – 628**  **1035 - 1273** |
|  |  | **ABC_TRANSPORTER_1** | [PS00211](https://prosite.expasy.org/cgi-bin/prosite/nicedoc.pl?PS00211) | *ABC transporters family* | **531 – 545**  **1176 - 1190** |
| APC | NP_000029.2 | **ARM_REPEAT** | [PS50176](https://prosite.expasy.org/cgi-bin/prosite/nicedoc.pl?PS50176) | *Armadillo/plakoglobin ARM repeat* | **660 - 702** |
| BAX | NP_004315.1 | **BH3** | [PS01259](https://prosite.expasy.org/cgi-bin/prosite/nicedoc.pl?PS01259) | *Apoptosis regulator, Bcl-2 family BH3 motif* | **59 - 73** |
|  |  | **BH1** | [PS01080](https://prosite.expasy.org/cgi-bin/prosite/nicedoc.pl?PS01080) | *Apoptosis regulator, Bcl-2 family BH1 motif* | **99 - 118** |
|  |  | **BH2** | [PS01258](https://prosite.expasy.org/cgi-bin/prosite/nicedoc.pl?PS01258) | *Apoptosis regulator, Bcl-2 family BH2 motif* | **151 - 162** |
| BMPR1A | NP_004320.2 | **GS** | [PS51256](https://prosite.expasy.org/cgi-bin/prosite/nicedoc.pl?PS51256) | *GS domain* | **204 - 233** |
|  |  | **PROTEIN_KINASE_DOM** | [PS50011](https://prosite.expasy.org/cgi-bin/prosite/nicedoc.pl?PS50011) | *Protein kinase domain* | **234 - 525** |
|  |  | **PROTEIN_KINASE_ATP** | [PS00107](https://prosite.expasy.org/cgi-bin/prosite/nicedoc.pl?PS00107) | *Protein kinases ATP-binding region* | **240 - 261** |
|  |  | **PROTEIN_KINASE_ST** | [PS00108](https://prosite.expasy.org/cgi-bin/prosite/nicedoc.pl?PS00108) | *Serine/Threonine protein kinases active-site* | **358 - 370** |
| CASP3 | NP_004337.2 | **CASPASE_P20** | [PS50208](https://prosite.expasy.org/cgi-bin/prosite/nicedoc.pl?PS50208) | *Caspase family p20 domain* | **43 - 167** |
|  |  | **CASPASE_P10** | [PS50207](https://prosite.expasy.org/cgi-bin/prosite/nicedoc.pl?PS50207) | *Caspase family p10 domain* | **183 - 277** |
|  |  | **CASPASE_HIS** | [PS01121](https://prosite.expasy.org/cgi-bin/prosite/nicedoc.pl?PS01121) | *Caspase family histidine active site* | **108 - 122** |
|  |  | **CASPASE_CYS** | [PS01122](https://prosite.expasy.org/cgi-bin/prosite/nicedoc.pl?PS01122) | *Caspase family cysteine active site* | **154 - 165** |
| CD44 | NP_000601.3 | **LINK_2** | [PS50963](https://prosite.expasy.org/cgi-bin/prosite/nicedoc.pl?PS50963) | *Link domain* | **32 - 120** |
|  |  | **LINK_1** | [PS01241](https://prosite.expasy.org/cgi-bin/prosite/nicedoc.pl?PS01241) | *Link domain* | **53 – 97** |
| CDH1 | NP_004351.1 | **CADHERIN_2** | [PS50268](https://prosite.expasy.org/cgi-bin/prosite/nicedoc.pl?PS50268) | *Cadherins domain* | **180 – 262**  **263 – 375**  **376 – 486**  **487 - 595**  **594 - 702** |
|  |  | **CADHERIN_1** | [PS00232](https://prosite.expasy.org/cgi-bin/prosite/nicedoc.pl?PS00232) | *Cadherin domain* | **250 – 260**  **363 – 373**  **583 - 593** |
| CDKN1B | NP_004055.1 | No Hit | | | |
| CEACAM5 | NP_004354.3 | **IG_LIKE** | [PS50835](https://prosite.expasy.org/cgi-bin/prosite/nicedoc.pl?PS50835) | *Ig-like domain* | **145 – 232**  **240 - 315**  **323 – 410**  **418 – 495**  **501 - 588**  **593 - 675** |
| CTNNA1 | NP_001894.2 | **VINCULIN_1** | [PS00663](https://prosite.expasy.org/cgi-bin/prosite/nicedoc.pl?PS00663) | *Vinculin family talin-binding region* | **178 - 198** |
| CTNNB1 | NP_001091679.1 | **ARM_REPEAT** | [PS50176](https://prosite.expasy.org/cgi-bin/prosite/nicedoc.pl?PS50176) | *Armadillo/plakoglobin ARM repeat* | **151 – 191**  **193 – 236**  **235 – 277**  **277 – 319**  **319 – 362**  **400 – 442**  **442 – 484**  **489 – 532**  **594 - 636** |
| DCC | NP_005206.2 | **IG_LIKE** | [PS50835](https://prosite.expasy.org/cgi-bin/prosite/nicedoc.pl?PS50835) | *Ig-like domain* | **11 – 135**  **139 – 229**  **234 – 326**  **331 - 416** |
|  |  | **FN3** | [PS50853](https://prosite.expasy.org/cgi-bin/prosite/nicedoc.pl?PS50853) | *Fibronectin type-III domain* | **431 - 524**  **530 - 620**  **625 – 718**  **728 - 821**  **846 – 942**  **947 - 1044** |
| EPCAM | NP_002345.2 | **THYROGLOBULIN_1_2** | [PS51162](https://prosite.expasy.org/cgi-bin/prosite/nicedoc.pl?PS51162) | *Thyroglobulin type-1 domain* | **63 - 135** |
|  |  | **THYROGLOBULIN_1_1** | [PS00484](https://prosite.expasy.org/cgi-bin/prosite/nicedoc.pl?PS00484) | *Thyroglobulin type-1 repeat* | **95 - 123** |
| KIT | NP_000213.1 | **IG_LIKE** | [PS50835](https://prosite.expasy.org/cgi-bin/prosite/nicedoc.pl?PS50835) | *Ig-like domain* | **212 - 308** |
|  |  | **PROTEIN_KINASE_DOM** | [PS50011](https://prosite.expasy.org/cgi-bin/prosite/nicedoc.pl?PS50011) | *Protein kinase domain* | **589 - 937** |
|  |  | **PROTEIN_KINASE_ATP** | [PS00107](https://prosite.expasy.org/cgi-bin/prosite/nicedoc.pl?PS00107) | *Protein kinases ATP-binding region* | **595 - 623** |
|  |  | **RECEPTOR_TYR_KIN_III** | [PS00240](https://prosite.expasy.org/cgi-bin/prosite/nicedoc.pl?PS00240) | *Receptor tyrosine kinase class III* | **648 - 661** |
|  |  | **PROTEIN_KINASE_TYR** | [PS00109](https://prosite.expasy.org/cgi-bin/prosite/nicedoc.pl?PS00109) | *Tyrosine protein kinases specific active-site* | **788 - 800** |
| KITLG | NP_000890.1 | No Hit | | | |
| KRAS | NP_004976.2 | **RAS** | [PS51421](https://prosite.expasy.org/cgi-bin/prosite/nicedoc.pl?PS51421) | *small GTPase Ras family* | **1 - 188** |
| MALT1 | NP_006776.1 | **IG_LIKE** | [PS50835](https://prosite.expasy.org/cgi-bin/prosite/nicedoc.pl?PS50835) | *Ig-like domain* | **125 – 201**  **212 - 305** |
|  |  | **CASPASE_P20** | [PS50208](https://prosite.expasy.org/cgi-bin/prosite/nicedoc.pl?PS50208) | *Caspase family p20 domain* | **341 - 419** |
| MET | NP_000236.2 | **SEMA** | [PS51004](https://prosite.expasy.org/cgi-bin/prosite/nicedoc.pl?PS51004) | *Sema domain* | **27 - 515** |
|  |  | **PROTEIN_KINASE_DOM** | [PS50011](https://prosite.expasy.org/cgi-bin/prosite/nicedoc.pl?PS50011) | *Protein kinase domain* | **1078 - 1345** |
|  |  | **PROTEIN_KINASE_ATP** | [PS00107](https://prosite.expasy.org/cgi-bin/prosite/nicedoc.pl?PS00107) | *Protein kinases ATP-binding region* | **1084 - 1110** |
|  |  | **PROTEIN_KINASE_TYR** | [PS00109](https://prosite.expasy.org/cgi-bin/prosite/nicedoc.pl?PS00109) | *Tyrosine protein kinases specific active-site* | **1200 - 1212** |
| MGMT | NP_002403.2 | **THIOL_PROTEASE_HIS** | [PS00639](https://prosite.expasy.org/cgi-bin/prosite/nicedoc.pl?PS00639) | *Eukaryotic thiol (cysteine) proteases histidine active site* | **58 - 68** |
|  |  | **MGMT** | [PS00374](https://prosite.expasy.org/cgi-bin/prosite/nicedoc.pl?PS00374) | *Methylated-DNA--protein-cysteine methyltransferase active site* | **174 - 180** |
| MMP2 | NP_001121363.1 | **FN2_2** | PS51092 | *Fibronectin type-II collagen-binding domain* | **178 – 226**  **236 – 284**  **294 - 342** |
|  |  | **HEMOPEXIN_2** | PS51642 | *Hemopexin repeat* | **422 – 466**  **467 – 513**  **515 – 563**  **564 - 610** |
|  |  | **CYSTEINE_SWITCH** | [PS00546](https://prosite.expasy.org/cgi-bin/prosite/nicedoc.pl?PS00546) | *Matrixins cysteine switch* | **50 - 57** |
|  |  | **FN2_1** | [PS00023](https://prosite.expasy.org/cgi-bin/prosite/nicedoc.pl?PS00023) | *Fibronectin type-II collagen-binding domain* | **183 - 224**  **241 - 282**  **299 - 340** |
|  |  | **ZINC_PROTEASE** | [PS00142](https://prosite.expasy.org/cgi-bin/prosite/nicedoc.pl?PS00142) | *Neutral zinc metallopeptidases, zinc-binding region* | **350 - 359** |
|  |  | **HEMOPEXIN** | [PS00024](https://prosite.expasy.org/cgi-bin/prosite/nicedoc.pl?PS00024) | *Hemopexin domain* | **556 - 571** |
| MSH2 | NP_000242.1 | **DNA_MISMATCH_REPAIR_2** | [PS00486](https://prosite.expasy.org/cgi-bin/prosite/nicedoc.pl?PS00486) | *DNA mismatch repair proteins mutS family* | **743 - 759** |
| MTHFR | NP_005948.3 | No Hit | | | |
| MUC1 | NP_001018016.1 | **SEA** | [PS50024](https://prosite.expasy.org/cgi-bin/prosite/nicedoc.pl?PS50024) | *SEA domain* | **44 - 157** |
| MYC | NP_002458.2 | **BHLH** | [PS50888](https://prosite.expasy.org/cgi-bin/prosite/nicedoc.pl?PS50888) | *Myc-type, basic helix-loop-helix (bHLH) domain* | **369 - 421** |
| PCNA | NP_002583.1 | **PCNA_1** | [PS01251](https://prosite.expasy.org/cgi-bin/prosite/nicedoc.pl?PS01251) | *Proliferating cell nuclear antigen* | **34 - 57** |
|  |  | **PCNA_2** | [PS00293](https://prosite.expasy.org/cgi-bin/prosite/nicedoc.pl?PS00293) | *Proliferating cell nuclear antigen* | **61 - 79** |
| PTEN | NP_000305.3 | **PPASE_TENSIN** | [PS51181](https://prosite.expasy.org/cgi-bin/prosite/nicedoc.pl?PS51181) | *Phosphatase tensin-type domain* | **14 - 185** |
|  |  | **C2_TENSIN** | [PS51182](https://prosite.expasy.org/cgi-bin/prosite/nicedoc.pl?PS51182) | *C2 tensin-type domain* | **190 - 350** |
|  |  | **TYR_PHOSPHATASE_1** | [PS00383](https://prosite.expasy.org/cgi-bin/prosite/nicedoc.pl?PS00383) | *Tyrosine specific protein phosphatases active site* | **122 - 132** |
| PTGS2 | NP_000954.1 | **EGF_3** | [PS50026](https://prosite.expasy.org/cgi-bin/prosite/nicedoc.pl?PS50026) | *EGF-like domain* | **17 - 55** |
|  |  | **PEROXIDASE_3** *superfamily* | [PS50292](https://prosite.expasy.org/cgi-bin/prosite/nicedoc.pl?PS50292) | *Animal heme peroxidase superfamily* | **97 - 598** |
| RUNX3 | NP_001026850.1 | **RUNT** | [PS51062](https://prosite.expasy.org/cgi-bin/prosite/nicedoc.pl?PS51062) | *Runt domain* | **68 - 196** |
| SDHA | NP_004159.2 | **FRD_SDH_FAD_BINDING** | [PS00504](https://prosite.expasy.org/cgi-bin/prosite/nicedoc.pl?PS00504) | *Fumarate reductase / succinate dehydrogenase FAD-binding site* | **97 - 106** |
| SDHB | NP_002991.2 | **2FE2S_FER_2** | [PS51085](https://prosite.expasy.org/cgi-bin/prosite/nicedoc.pl?PS51085) | *2Fe-2S ferredoxin-type iron-sulfur binding domain* | **40 - 133** |
|  |  | **4FE4S_FER_2** | [PS51379](https://prosite.expasy.org/cgi-bin/prosite/nicedoc.pl?PS51379) | *4Fe-4S ferredoxin-type iron-sulfur binding domain* | **176 - 206** |
|  |  | **2FE2S_FER_1** | [PS00197](https://prosite.expasy.org/cgi-bin/prosite/nicedoc.pl?PS00197) | *2Fe-2S ferredoxin-type iron-sulfur binding region* | **93 - 101** |
|  |  | **4FE4S_FER_1** | [PS00198](https://prosite.expasy.org/cgi-bin/prosite/nicedoc.pl?PS00198) | *4Fe-4S ferredoxin-type iron-sulfur binding region* | **186 - 197** |
| SDHD | NP_002993.1 | No Hit | | | |
| SMAD4 | NP_005350.1 | **MH1** | [PS51075](https://prosite.expasy.org/cgi-bin/prosite/nicedoc.pl?PS51075) | *MAD homology domain 1 (MH1)* | **18 - 142** |
|  |  | **MH2** | [PS51076](https://prosite.expasy.org/cgi-bin/prosite/nicedoc.pl?PS51076) | *MAD homology domain 2 (MH2)* | **323 - 552** |
| STK11 | NP_000446.1 | **PROTEIN_KINASE_DOM** | [PS50011](https://prosite.expasy.org/cgi-bin/prosite/nicedoc.pl?PS50011) | *Protein kinase domain* | **49 - 309** |
|  |  | **PROTEIN_KINASE_ATP** | [PS00107](https://prosite.expasy.org/cgi-bin/prosite/nicedoc.pl?PS00107) | *Protein kinases ATP-binding region* | **55 - 78** |
|  |  | **PROTEIN_KINASE_ST** | [PS00108](https://prosite.expasy.org/cgi-bin/prosite/nicedoc.pl?PS00108) | *Serine/Threonine protein kinases active-site* | **172 - 184** |
| TNF | NP_000585.2 | **TNF_2** | [PS50049](https://prosite.expasy.org/cgi-bin/prosite/nicedoc.pl?PS50049) | *TNF family* | **89 - 233** |
|  |  | **TNF_1** | [PS00251](https://prosite.expasy.org/cgi-bin/prosite/nicedoc.pl?PS00251) | *TNF family* | **124 - 140** |
| TP53 | NP_000537.3 | **P53** | [PS00348](https://prosite.expasy.org/cgi-bin/prosite/nicedoc.pl?PS00348) | *p53 family* | **237 - 249** |
| VEGFA | NP_001020537.2 | **PDGF_2** | [PS50278](https://prosite.expasy.org/cgi-bin/prosite/nicedoc.pl?PS50278) | *Platelet-derived growth factor (PDGF) family* | **219 - 315** |
|  |  | **PDGF_1** | [PS00249](https://prosite.expasy.org/cgi-bin/prosite/nicedoc.pl?PS00249) | *Platelet-derived growth factor (PDGF) family* | **255 - 267** |
|  |  |  |  |  |  |
