# Supplementary material for: Investigation of pathogenic germline variants in gastric cancer and development of “GasCanBase” database
Source: Cancer Rep (Hoboken). 2023 Oct 22;6(12):e1906. doi: 10.1002/cnr2.1906 (PMC10728505; doi:10.1002/cnr2.1906)
Supplement: Supplementary file 1 — Data S1 Supporting Information. [file CNR2-6-e1906-s001.zip › Supplementary File/Table S88. Free energy of the wild and mutant model..docx]

| **Gene** |  | **Start** | **End** |
| --- | --- | --- | --- |
| ABCB1 | Wild | Energy: -228932.3 kJ/mol  Score: -3.15 | Energy: -693195.6 kJ/mol  Score: -0.26 |
|  | Mutant (I736K) | Energy: - 219546.0 kJ/mol  Score: -3.14 | Energy: - 704350.8 kJ/mol  Score: -0.14 |
| APC | Wild | Energy: 253676.3 kJ/mol  Score: -5.98 | Energy: - 185679.0 kJ/mol  Score: -2.77 |
|  | Mutant | Energy: - kJ/mol  Score: - | Energy: - kJ/mol  Score: - |
| AURKA | Wild | Energy: -110509.2 kJ/mol  Score: -1.76 | Energy: - 211760.4 kJ/mol  Score: 0.49 |
|  | Mutant | Energy: - 109075.3 kJ/mol  Score: -1.74 | Energy: - 211152.7 kJ/mol  Score: 0.47 |
| BAX | Wild | Energy: -44201.6 kJ/mol  Score: -2.28 | Energy: -111968.6 kJ/mol  Score: 0.21 |
|  | Mutant | Energy: 1246837295249.2 kJ/mol  Score: -2.04 | Energy: - 115451.8kJ/mol  Score: 0.22 |
| BMPR1A | Wild | Energy: - 124063.6 kJ/mol  Score: -1.81 | Energy: - 263193.2 kJ/mol  Score: 0.45 |
|  | Mutant | Energy: - 113106.0 kJ/mol  Score: -1.80 | Energy: - 259633.6 kJ/mol  Score: 0.30 |
| CASP3 | Wild | Energy: - 80825.4 kJ/mol  Score: -1.66 | Energy: - 149067.7 kJ/mol  Score: 0.02 |
|  | Mutant | Energy: - 75396.2 kJ/mol  Score: -1.71 | Energy: - 149381.7 kJ/mol  Score: 0.26 |
| CD44 | Wild | Energy: - kJ/mol  Score: - | Energy: - kJ/mol  Score: - |
|  | Mutant | Energy: - kJ/mol  Score: - | Energy: - kJ/mol  Score: - |
| CDH1 | Wild | Energy: - kJ/mol  Score: - | Energy: - kJ/mol  Score: - |
|  | Mutant | Energy: - kJ/mol  Score: - | Energy: - kJ/mol  Score: - |
| CDKN1B | Wild | Energy: - 46391.7 kJ/mol  Score: -2.96 | Energy: - 90998.3 kJ/mol  Score: 0.04 |
|  | Mutant | Energy: - 45608.7 kJ/mol  Score: -2.57 | Energy: - 88968.4 kJ/mol  Score: 0.19 |
| CEACAM5 | Wild | Energy: 40371.6 kJ/mol  Score: -5.11 | Energy: - 287479.2 kJ/mol  Score: -2.38 |
|  | Mutant | Energy: 23517.3 kJ/mol  Score: -5.05 | Energy: -290976.9 kJ/mol  Score: -2.15 |
| CTNNA1 | Wild | Energy: - 143970.4 kJ/mol  Score: -2.38 | Energy: - 516486.2 kJ/mol  Score: -0.12 |
|  | Mutant | Energy: - 174856.9 kJ/mol  Score: -2.46 | Energy: - 517807.2 kJ/mol  Score: -0.14 |
| CTNNB1 | Wild | Energy: 29895068367856.8 kJ/mol  Score: -2.88 | Energy: -405827.6 kJ/mol  Score: 0.11 |
|  | Mutant | Energy: -87118.3 kJ/mol  Score: -3.13 | Energy: - 405297.0 kJ/mol  Score: 0.14 |
| DCC | Wild | Energy: - kJ/mol  Score: - | Energy: - kJ/mol  Score: - |
|  | Mutant | Energy: - kJ/mol  Score: - | Energy: - kJ/mol  Score: - |
| EPCAM | Wild | Energy: - 86693.8 kJ/mol  Score: -1.42 | Energy: -157223.6 kJ/mol  Score: 0.25 |
|  | Mutant | Energy: - 84185.7 kJ/mol  Score: -1.55 | Energy: - 155508.1 kJ/mol  Score: 0.24 |
| FOS | Wild | Energy: - kJ/mol  Score: - | Energy: - kJ/mol  Score: - |
|  | Mutant | Energy: - kJ/mol  Score: - | Energy: - kJ/mol  Score: - |
| KIT | Wild | Energy: - 110979.8 kJ/mol  Score: -3.19 | Energy: - 463245.9 kJ/mol  Score: -0.73 |
|  | Mutant | Energy: - 84653.1 kJ/mol  Score: -3.33 | Energy: - 461750.0 kJ/mol  Score: -0.68 |
| KITLG | Wild | Energy: -35046.3 kJ/mol  Score: -2.74 | Energy: - 128468.4 kJ/mol  Score: -0.17 |
|  | Mutant | Energy: - 49403.0 kJ/mol  Score: -2.53 | Energy: - 127426.4 kJ/mol  Score: -0.05 |
| KRAS | Wild | Energy: - 62065.8 kJ/mol  Score: -1.60 | Energy: - 107992.9 kJ/mol  Score: 0.29 |
|  | Mutant | Energy: -60282.7 kJ/mol  Score: -1.66 | Energy: - 109671.5 kJ/mol  Score: 0.14 |
| KRT20 | Wild | Energy: -88743.8 kJ/mol  Score: -2.93 | Energy: - 230116.6 kJ/mol  Score: 0.14 |
|  | Mutant | Energy: - kJ/mol  Score: - | Energy: - kJ/mol  Score: - |
| MATL1 | Wild | Energy: - 49027.8 kJ/mol  Score: -3.43 | Energy: - 384417.3 kJ/mol  Score: -1.01 |
|  | Mutant | Energy: 33955.7 kJ/mol  Score: -3.87 | Energy: - 374740.8 kJ/mol  Score: -1.40 |
| MET | Wild | Energy: - kJ/mol  Score: - | Energy: - kJ/mol  Score: - |
|  | Mutant | Energy: - kJ/mol  Score: - | Energy: - kJ/mol  Score: - |
| MGMT | Wild | Energy: - 38352.5 kJ/mol  Score: -2.07 | Energy: - 109071.1 kJ/mol  Score: -0.06 |
|  | Mutant | Energy: -48219.3 kJ/mol  Score: -2.35 | Energy: - 106194.0 kJ/mol  Score: -0.12 |
| MMP2 | Wild | Energy: -119461.6 kJ/mol  Score: -2.75 | Energy: - 310840.3 kJ/mol  Score: -0.47 |
|  | Mutant | Energy: - 101866.3 kJ/mol  Score: -2.66 | Energy: - 310822.3 kJ/mol  Score: -0.39 |
| MSH2 | Wild | Energy: - 246346.6 kJ/mol  Score: -2.38 | Energy: - 525451.1 kJ/mol  Score: 0.10 |
|  | Mutant | Energy: -222339.3 kJ/mol  Score: -2.37 | Energy: - 525004.8 kJ/mol  Score: 0.04 |
| MTHFR | Wild | Energy: - kJ/mol  Score: - | Energy: - kJ/mol  Score: - |
|  | Mutant | Energy: - kJ/mol  Score: - | Energy: - kJ/mol  Score: - |
| MUC1 | Wild | Energy: 7828.7 kJ/mol  Score: -3.73 | Energy: - 101724.3 kJ/mol  Score: -0.91 |
|  | Mutant | Energy: - 16196.9 kJ/mol  Score: -3.55 | Energy: - 106957.6 kJ/mol  Score: -0.92 |
| MYC | Wild | Energy: - kJ/mol  Score: - | Energy: - kJ/mol  Score: - |
|  | Mutant | Energy: - kJ/mol  Score: - | Energy: - kJ/mol  Score: - |
| PCNA | Wild | Energy: - 77404..5 kJ/mol  Score: -1.81 | Energy: - 142749.6 kJ/mol  Score: -0.02 |
|  | Mutant | Energy: - 76677.2 kJ/mol  Score: -1.59 | Energy: -142645.2 kJ/mol  Score: 0.02 |
| PIK3CA | Wild | Energy: -329740.5 kJ/mol  Score: -1.75 | Energy: -618215.0 kJ/mol  Score: 0.15 |
|  | Mutant | Energy: - 332214.3 kJ/mol  Score: -1.78 | Energy: -620732.6 kJ/mol  Score: 0.05 |
| PTEN | Wild | Energy: - 80325.4 kJ/mol  Score: -2.80 | Energy: -219402.7 kJ/mol  Score: -0.26 |
|  | Mutant | Energy: - 47165.1kJ/mol  Score: -3.14 | Energy: - 220498.4 kJ/mol  Score: -0.20 |
| PTGS2 | Wild | Energy: - 166483.6 kJ/mol  Score: -1.74 | Energy: - 324119.9 kJ/mol  Score: -0.04 |
|  | Mutant | Energy: - 169081.9 kJ/mol  Score: -1.77 | Energy: - 322080.1 kJ/mol  Score: -0.14 |
| RUNX3 | Wild | Energy: -77749.1 kJ/mol  Score: -1.95 | Energy: -168915.0 kJ/mol  Score: 0.31 |
|  | Mutant | Energy: - 76651.8 kJ/mol  Score: -1.86 | Energy: - 165932.2 kJ/mol  Score: 0.15 |
| SDHA | Wild | Energy: - 188494.4 kJ/mol  Score: -1.77 | Energy: - 344652.8 kJ/mol  Score: -0.33 |
|  | Mutant | Energy: - 186667.9 kJ/mol  Score: -1.83 | Energy: - 351801.6 kJ/mol  Score: -0.18 |
| SDHB | Wild | Energy: - 53753.8 kJ/mol  Score: -2.43 | Energy: - 143156.0 kJ/mol  Score: -0.14 |
|  | Mutant | Energy: - 56514.4 kJ/mol  Score: -2.37 | Energy: - 144346.3 kJ/mol  Score: -0.36 |
| SDHD | Wild | Energy: - 25207.6 kJ/mol  Score: -1.77 | Energy: -67087.6 kJ/mol  Score: 0.62 |
|  | Mutant | Energy: - 19209.0 kJ/mol  Score: -1.45 | Energy: - 68212.6 kJ/mol  Score: 0.78 |
| SMAD4 | Wild | Energy: 246739.3 kJ/mol  Score: -4.22 | Energy: - 216491.4 kJ/mol  Score: -1.16 |
|  | Mutant | Energy: 1331955736389.8 kJ/mol  Score: -3.69 | Energy: -218659.6 kJ/mol  Score: -1.08 |
| STK11 | Wild | Energy: - 113822.9 kJ/mol  Score: -2.13 | Energy: - 226195.4 kJ/mol  Score: 0.03 |
|  | Mutant | Energy: - 107619.6 kJ/mol  Score: -2.21 | Energy: -228077.4 kJ/mol  Score: 0.06 |
| TNF | Wild | Energy: - 38161.2 kJ/mol  Score: -2.34 | Energy: -105006.6 kJ/mol  Score: -0.17 |
|  | Mutant | Energy: - 38240.2 kJ/mol  Score: -2.34 | Energy: -103875.3 kJ/mol  Score: -0.17 |
| TP53 | Wild | Energy: - 26166.1 kJ/mol  Score: -4.17 | Energy: - 181787.1 kJ/mol  Score: -1.33 |
|  | Mutant | Energy: 11681.3 kJ/mol  Score: -4.35 | Energy: - 171401.7 kJ/mol  Score: -1.72 |
| VEGFA | Wild | Energy: - kJ/mol  Score: - | Energy: - kJ/mol  Score: - |
|  | Mutant | Energy: - kJ/mol  Score: - | Energy: - kJ/mol  Score: - |
