# Supplementary figures and images for: Investigation of pathogenic germline variants in gastric cancer and development of “GasCanBase” database
Source: Cancer Rep (Hoboken). 2023 Oct 22;6(12):e1906. doi: 10.1002/cnr2.1906 (PMC10728505; doi:10.1002/cnr2.1906)

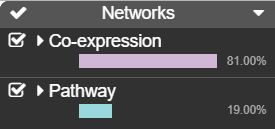

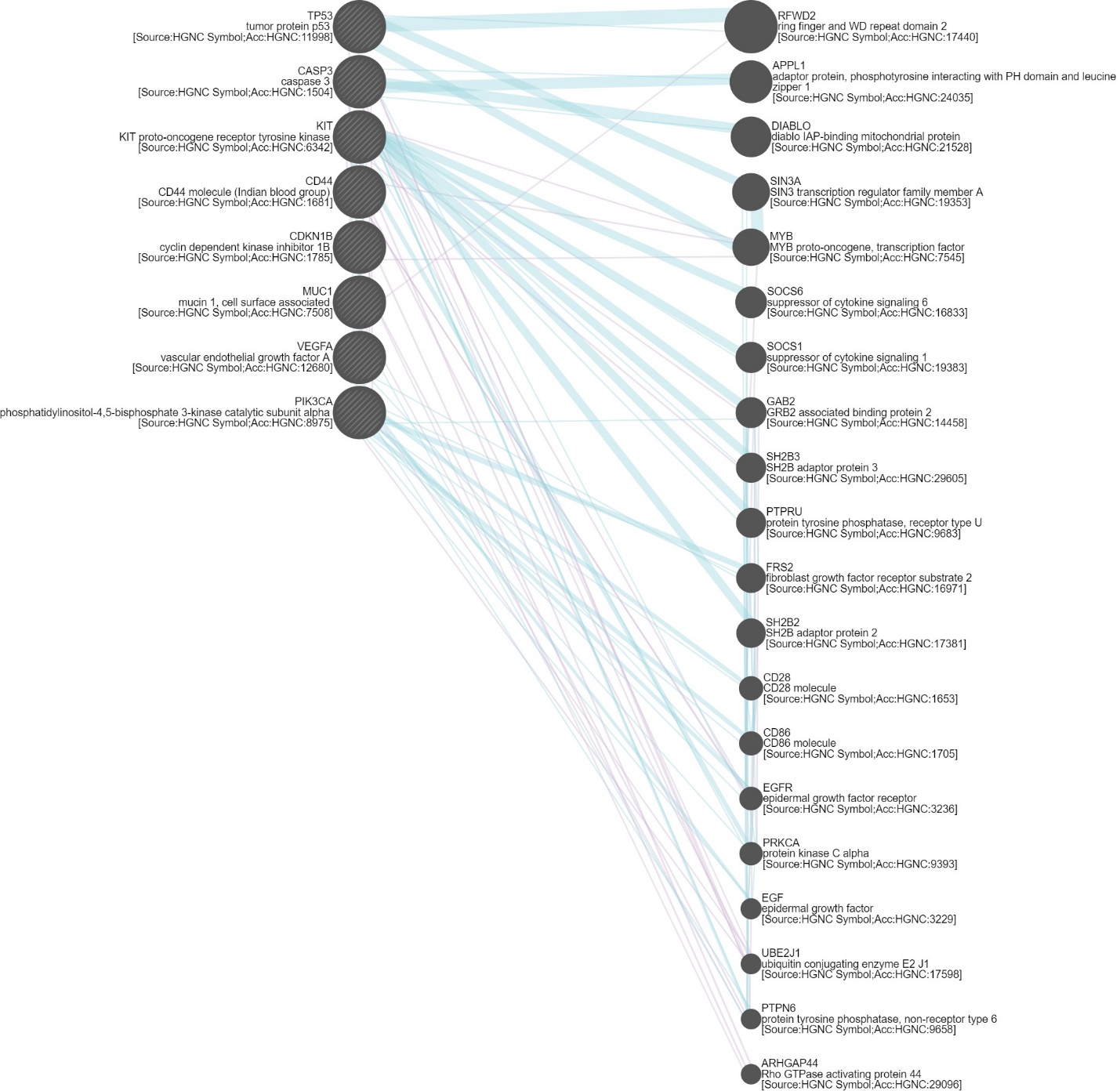


**Figure S1: Gene networking of Gastric and Bladder Cancer genes**

Supplement: Supplementary file 1 — Data S1 Supporting Information. [file CNR2-6-e1906-s001.zip › Supplementary File/Figure S1. Gene networking of Gastric and Bladder Cancer genes.docx]

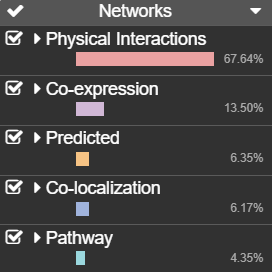


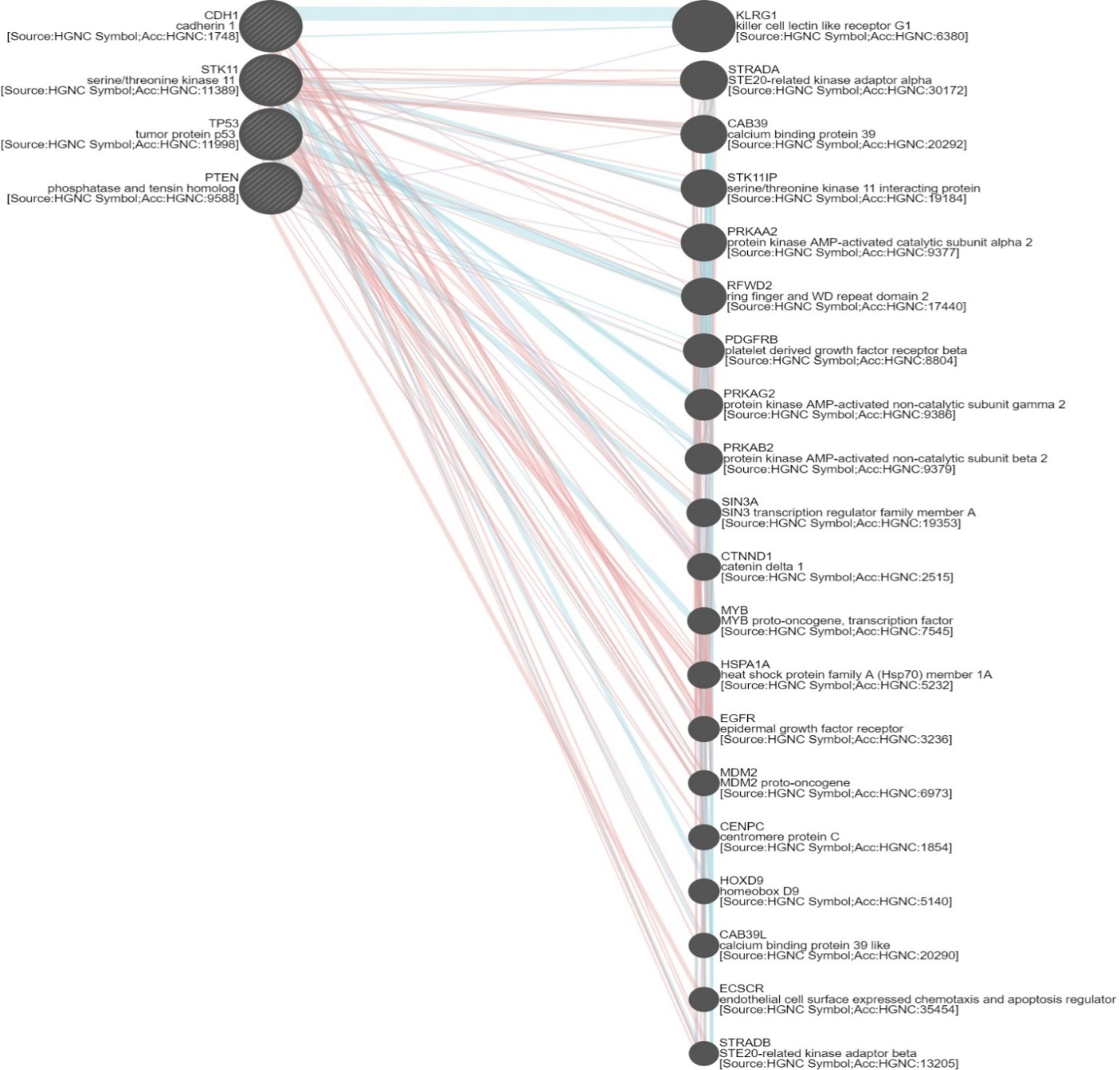


**Figure S1.1. Gene networking of Gastric and Breast Cancer genes**

Supplement: Supplementary file 1 — Data S1 Supporting Information. [file CNR2-6-e1906-s001.zip › Supplementary File/Figure S2. Gene networking of Gastric and Breast Cancer genes.docx]

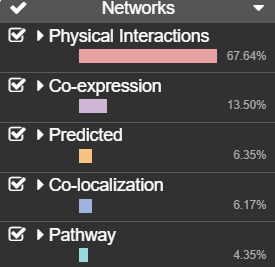

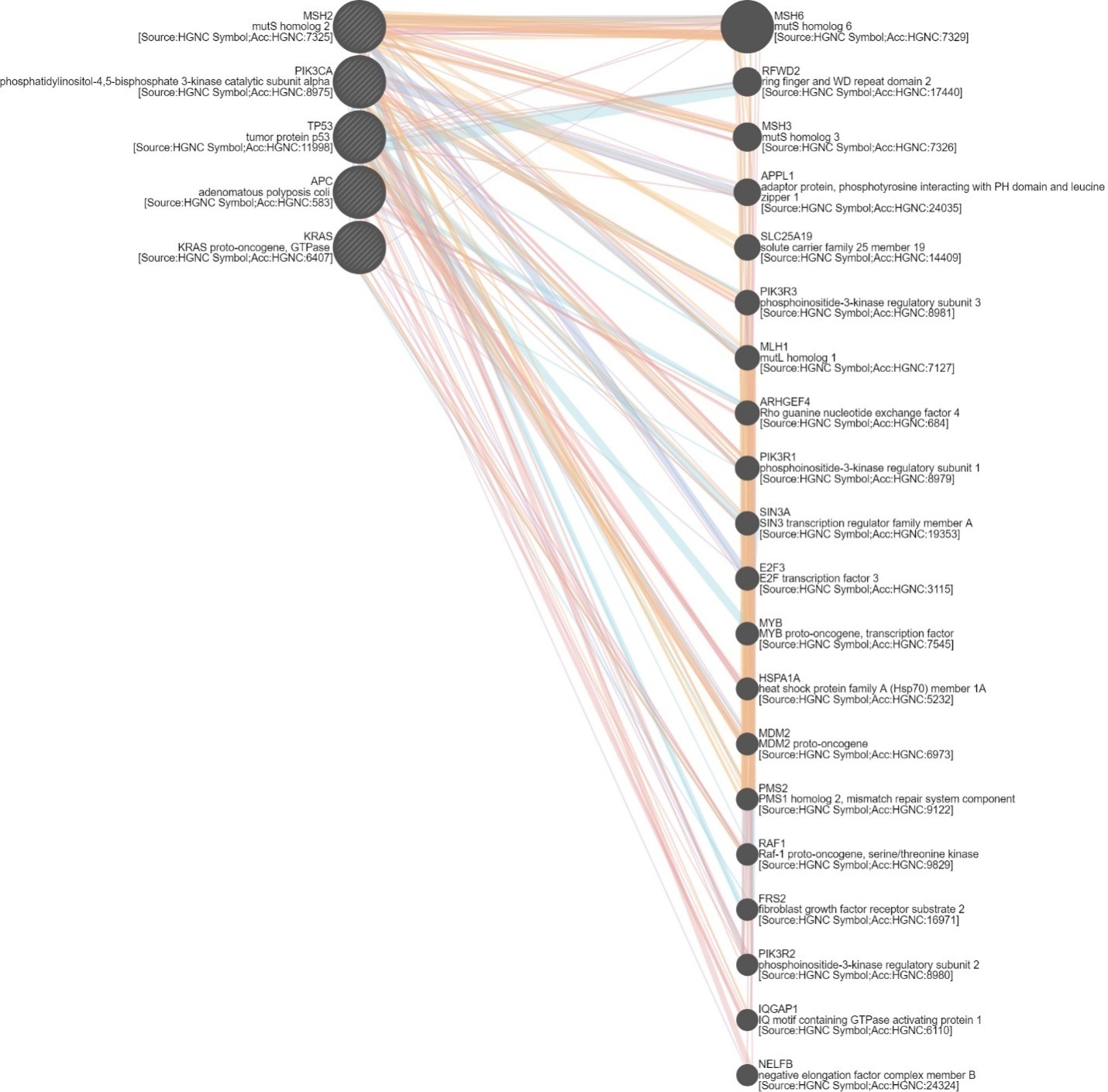


**Figure S1.1. Gene networking of Gastric and Colon Cancer genes**

Supplement: Supplementary file 1 — Data S1 Supporting Information. [file CNR2-6-e1906-s001.zip › Supplementary File/Figure S3. Gene networking of Gastric and Colon Cancer genes.docx]

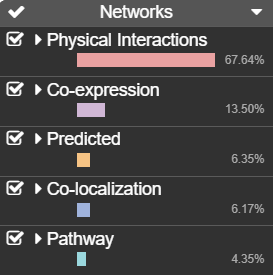

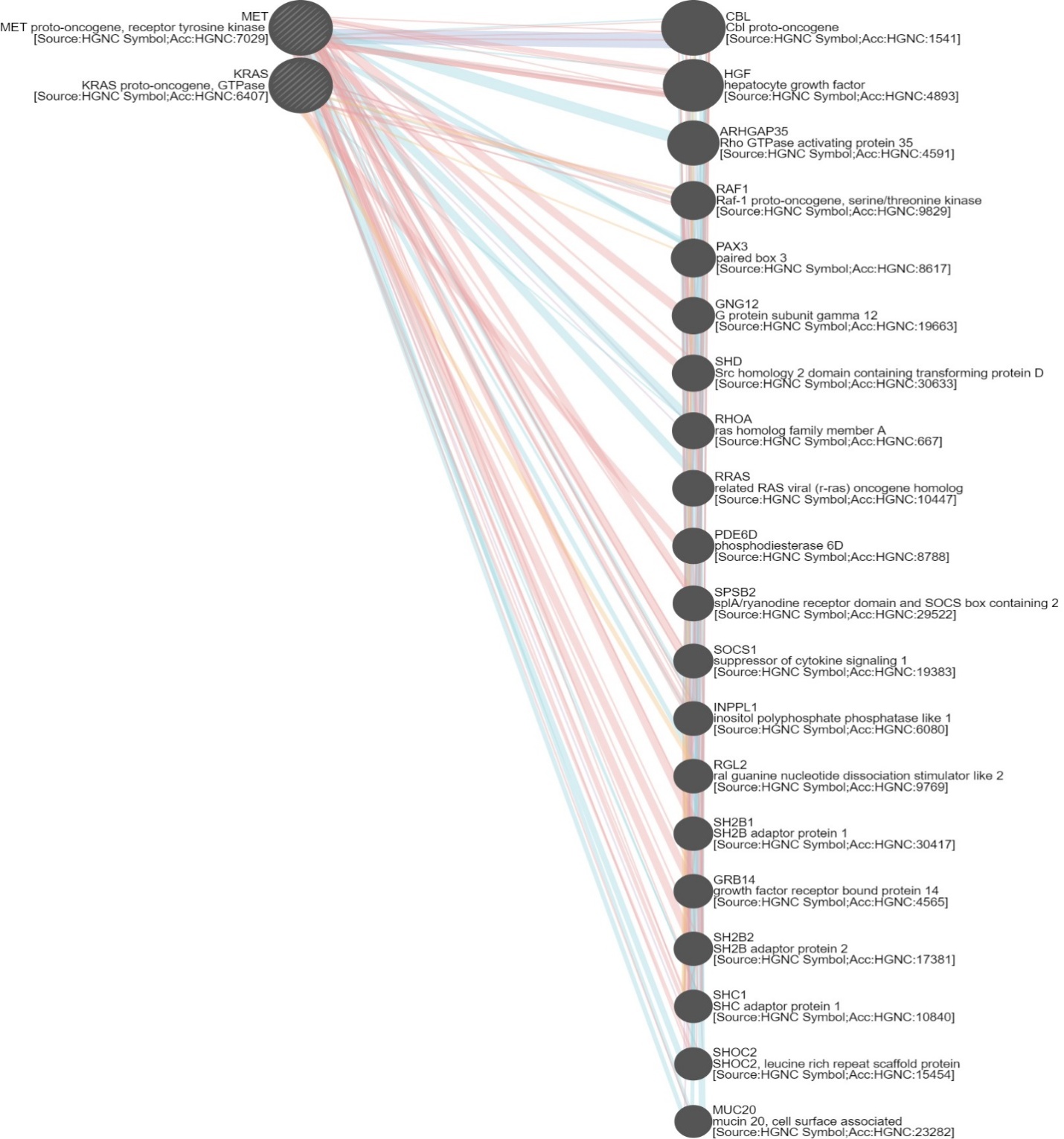


**Figure S1.3. Gene networking of Gastric and Lung Cancer genes**

Supplement: Supplementary file 1 — Data S1 Supporting Information. [file CNR2-6-e1906-s001.zip › Supplementary File/Figure S4. Gene networking of Gastric and Lung Cancer genes.docx]

## Slide 1
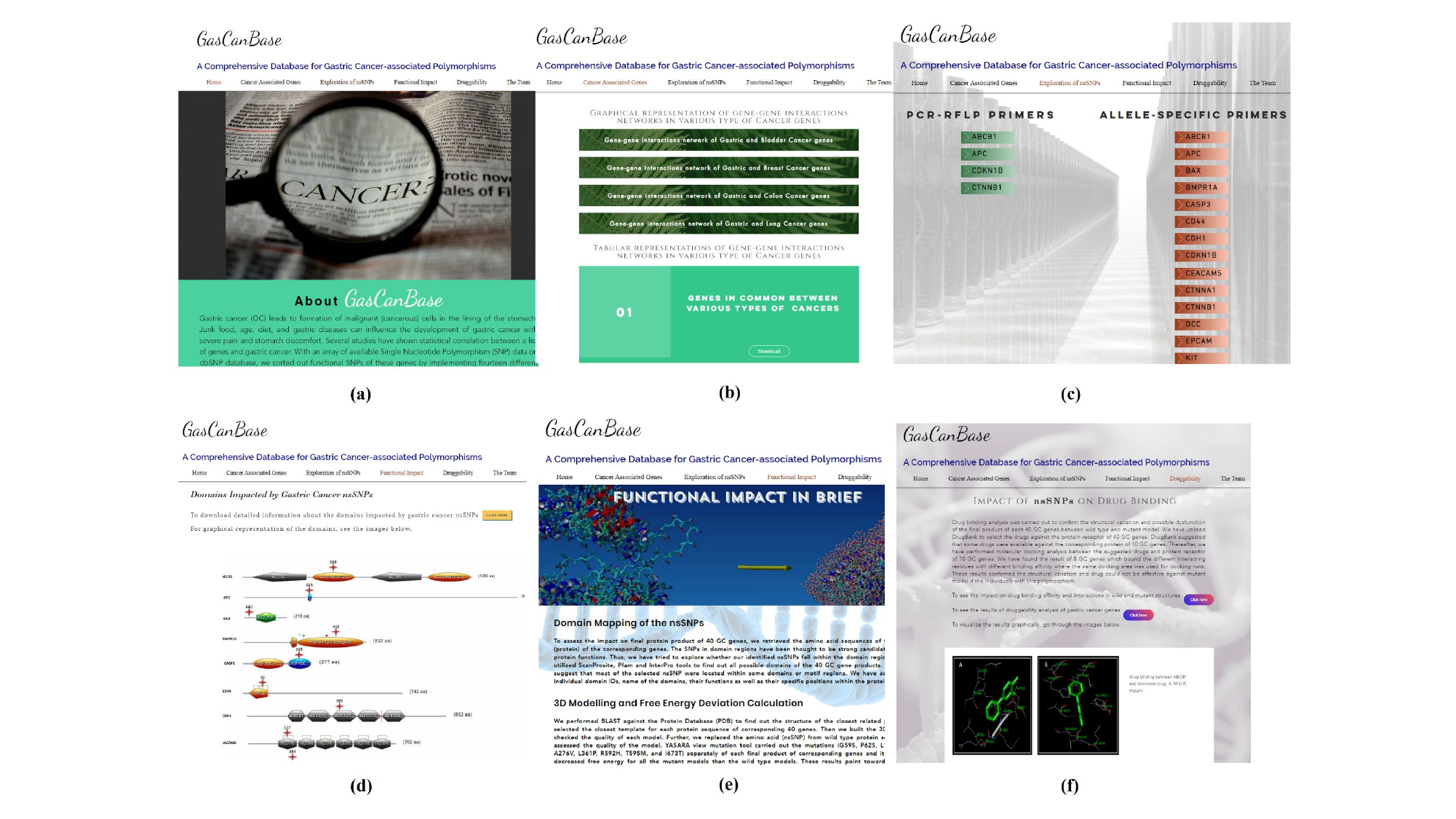

#

Supplement: Supplementary file 1 — Data S1 Supporting Information. [file CNR2-6-e1906-s001.zip › Supplementary File/Figure S46. The snapshot of the ‘GasCanBase’ database.pptx]
